# Stability of an HTLV-HIV coinfection model with multiple delays and CTL-mediated immunity

**DOI:** 10.1186/s13662-021-03416-7

**Published:** 2021-05-25

**Authors:** N. H. AlShamrani

**Affiliations:** 1 Department of Mathematics, Faculty of Science, King Abdulaziz University, P.O. Box 80203, Jeddah, 21589 Saudi Arabia; 2 Department of Mathematics, Faculty of Science, University of Jeddah, P.O. Box 80327, Jeddah, 21589 Saudi Arabia

**Keywords:** 34D20, 34D23, 37N25, 92B05, HTLV-HIV coinfection, CTL immune response, Intracellular delay, Mitotic transmission, Global stability, Lyapunov function

## Abstract

In the literature, several mathematical models have been formulated and developed to describe the within-host dynamics of either human immunodeficiency virus (HIV) or human T-lymphotropic virus type I (HTLV-I) monoinfections. In this paper, we formulate and analyze a novel within-host dynamics model of HTLV-HIV coinfection taking into consideration the response of cytotoxic T lymphocytes (CTLs). The uninfected $\mathrm{CD} 4^{+}\mathrm{T}$ cells can be infected via HIV by two mechanisms, free-to-cell and infected-to-cell. On the other hand, the HTLV-I has two modes for transmission, (i) horizontal, via direct infected-to-cell touch, and (ii) vertical, by mitotic division of active HTLV-infected cells. It is well known that the intracellular time delays play an important role in within-host virus dynamics. In this work, we consider six types of distributed-time delays. We investigate the fundamental properties of solutions. Then, we calculate the steady states of the model in terms of threshold parameters. Moreover, we study the global stability of the steady states by using the Lyapunov method. We conduct numerical simulations to illustrate and support our theoretical results. In addition, we discuss the effect of multiple time delays on stability of the steady states of the system.

## Introduction

During the past several decades many human viruses and their associated diseases, such as human immunodeficiency virus (HIV), hepatitis C virus (HCV), hepatitis B virus (HBV), dengue virus, human T-lymphotropic virus type I (HTLV-I), and recently coronavirus, have been recognized. Human body can be infected by more that one virus at the same time such as HTLV-HIV, coronavirus/influenza, HCV-HIV, HBV-HIV, HCV-HBV, and malaria-HIV. HTLV and HIV are universal public health matters. HTLV and HIV are two viruses which infect most effective immune cells, $\mathrm{CD} 4^{+}\mathrm{T}$ cells. Adult T-cell leukemia (ATL) and HTLV-I-associated myelopathy/tropical spastic paraparesis (HAM/TSP) are the last stage of HTLV-I infection. Chronic HIV infection leads to acquired immunodeficiency syndrome (AIDS). Both HTLV and HIV have the same ways of transmission such as sharing contaminated needles and unprotected sexual contact with infected partners. Over the last 10 years HTLV-HIV coinfection has been widely documented (see e.g. [1–3], and [4]).

### Mathematical models

Mathematical models of HIV and HTLV-I dynamics have become efficient tools to biological and medical scientists. These models can provide a deeper understanding of within-host virus dynamics and assist in predicting *the impact of antiviral drug efficacy on viral infection progression* (see e.g. [5–16]).

- *HIV monoinfection model*: The standard HIV dynamics model under the effect of cytotoxic T lymphocytes (CTLs) has been formulated by Nowak and Bangham [17] as follows:
  1 $$ \textstyle\begin{cases}\frac{dS(t)}{dt}=\eta -\varrho S(t)-\vartheta _{1}S(t)V(t), \\ \frac{dI(t)}{dt}=\vartheta _{1}S(t)V(t)-aI(t)-\mu _{1}C^{I}(t)I(t), \\ \frac{dV(t)}{dt}=bI(t)-\varepsilon V(t), \\ \frac{dC^{I}(t)}{dt}=\sigma _{1}C^{I}(t)I(t)-\pi _{1}C^{I}(t), \end{cases} $$
  where $S(t)$, $I(t)$, $V(t)$, and $C^{I}(t)$ are the concentrations of uninfected $\mathrm{CD} 4^{+}\mathrm{T}$ cells, active HIV-infected cells, free HIV particles, and HIV-specific CTLs, respectively, and *t* is the time. *η* refers to the generation rate of the uninfected $\mathrm{CD} 4^{+}\mathrm{T}$ cells. The uninfected $\mathrm{CD} 4^{+}\mathrm{T}$ cells are infected via HIV particles (free-to-cell infection) at rate $\vartheta _{1}SV$. The HIV-infected cells produce HIV particles at rate *bI*. The stimulation rate of effective HIV-specific CTLs due to the presence of HIV-infected cells is defined by $\sigma _{1}C^{I}I$. The term $\mu _{1}C^{I}I$ accounts for the killing rate of HIV-infected cells due to its specific CTLs. The four compartments *S*, *I*, *V*, and $C^{I}$ have normal death rates *ϱS*, *aI*, *εV*, and $\pi _{1}C^{I}$, respectively. Several extensions on model (1) have been accomplished (see e.g. [18–20]).
- *HTLV-I monoinfection model*: The within-host dynamics of HTLV-I has been mathematically modeled in several papers [21–24]. CTL immunity has been included into the HTLV-I dynamics models in many works (see e.g. [25–33]). Lim and Maini [28] have formulated a model for HTLV-I dynamics under the consideration of CTL immunity and mitotic division of active HTLV-infected cells as follows:
  2 $$ \textstyle\begin{cases}\frac{dS(t)}{dt}=\eta -\varrho S(t)-\vartheta _{3}S(t)Y(t), \\ \frac{dE(t)}{dt}=\vartheta _{3}S(t)Y(t)+\mathcal{K}r^{\ast }Y(t)- ( \psi +\omega ) E(t), \\ \frac{dY(t)}{dt}=\psi E(t)-\delta ^{\ast }Y(t)-\mu _{2}C^{Y}(t)Y(t), \\ \frac{dC^{Y}(t)}{dt}=\sigma _{2}Y(t)-\pi _{2}C^{Y}(t), \end{cases} $$
  where $E(t)$, $Y(t)$, and $C^{Y}(t)$ are the concentrations of latent HTLV-infected cells, active HTLV-infected cells, and HTLV-specific CTLs at time *t*, respectively. The term $\vartheta _{3}SY$ denotes the infected-to-cell contact rate between HTLV-infected cells and uninfected $\mathrm{CD} 4^{+}\mathrm{T}$ cells (horizontal transmission). The active HTLV-infected cells transmit vertically to latent compartment at rate $\mathcal{K}r^{\ast }Y$ (mitotic transmission), where $\mathcal{K}\in (0,1)$. The HTLV-specific CTLs kill the active HTLV-infected cells at rate $\mu _{2}C^{Y}Y$ and are stimulated at rate $\sigma _{2}Y$. The term *ψE* denotes the activation rate of latent HTLV-infected cells. The death rates of *E*, *Y*, and $C^{Y}$ are given by *ωE*, $\delta ^{\ast }Y$, and $\pi _{2}C^{Y}$, respectively.
- *HTLV-HIV coinfection model*: Elaiw and AlShamrani [34] have recently formulated an HTLV-HIV coinfection model as follows:
  3 $$ \textstyle\begin{cases}\frac{dS(t)}{dt}=\eta -\varrho S(t)-\vartheta _{1}S(t)V(t)-\vartheta _{2}S(t)I(t)- \vartheta _{3}S(t)Y(t), \\ \frac{dL(t)}{dt}= ( 1-\beta ) (\vartheta _{1}S(t)V(t)+ \vartheta _{2}S(t)I(t))- ( \lambda +\gamma ) L(t), \\ \frac{dI(t)}{dt}=\beta (\vartheta _{1}S(t)V(t)+\vartheta _{2}S(t)I(t))+ \lambda L(t)-aI(t)-\mu _{1}C^{I}(t)I(t), \\ \frac{dE(t)}{dt}=\varphi \vartheta _{3}S(t)Y(t)- ( \psi +\omega ) E(t), \\ \frac{dY(t)}{dt}=\psi E(t)-\delta ^{\ast }Y(t)-\mu _{2}C^{Y}(t)Y(t), \\ \frac{dV(t)}{dt}=bI(t)-\varepsilon V(t), \\ \frac{dC^{I}(t)}{dt}=\sigma _{1}C^{I}(t)I(t)-\pi _{1}C^{I}(t), \\ \frac{dC^{Y}(t)}{dt}=\sigma _{2}C^{Y}(t)Y(t)-\pi _{2}C^{Y}(t), \end{cases} $$
  where $L(t)$ is the concentration of latent HIV-infected cells. The term $\vartheta _{2}SI$ describes the infection rate of uninfected $\mathrm{CD} 4^{+}\mathrm{T}$ cells by HIV-infected cells. *λL* and *γL* are the activation and death rates of latent HIV-infected cells. The parameter $\beta \in ( 0,1 ) $ represents the part of newly HIV-infected cells that becomes active, and the other part $1-\beta $ enters a latent stage. The parameter $\varphi \in ( 0,1 ) $ refers to the part of newly HTLV-infected cells that become latent.

Intracellular delay plays a crucial role in within-host virus dynamics and is defined as the time lapse between viral entry a cell and its production. In case of HIV, it has been estimated that the time between the HIV enters a target cell until producing new HIV particles is about 0.9 days [35]. Time delay has also an important effect in HTLV-I infection. Several works have been devoted to developing mathematical models with time delays to describe the dynamics of HIV (see e.g. [36–43]) and HTLV (see e.g. [44–53]).

Our aim is to take model (3) to further destination by incorporating multiple intracellular time delays and mitotic transmission. We study the fundamental and global properties of the system, then we present numerical simulation. The outcomes of this paper will help clinicians to estimate the suitable time to start the treatment. Our model may be helpful to study different coinfections such as influenza-coronavirus, HCV-HIV, HBV-HIV, and malaria-HIV. *It is interesting to note that fractional-order differential equations (FODEs) have been widely studied in several works (see e.g. [*54*–*57*]). Modeling and analysis of HIV dynamics with FODEs have been investigated in many papers (see e.g. [*58*–*60*]). Clinicians can use the information (in terms of behavior predictions) of fractional-order systems to fit patients’ data with the most appropriate noninteger-order index. As a future work, our coinfection model can be formulated as a system of FODEs.*

## The multiple delays model

In this section, we extend system (3) by taking under consideration multiple types of distributed-time delays and mitosis of active HTLV-infected cells. We achieve this goal by considering the following system of delay differential equations (DDEs):

4 $$ \textstyle\begin{cases}\frac{dS(t)}{dt}=\eta -\varrho S(t)-\vartheta _{1}S(t)V(t)-\vartheta _{2}S(t)I(t)- \vartheta _{3}S(t)Y(t), \\ \frac{dL(t)}{dt}= ( 1-\beta ) \int _{0}^{\kappa _{1}}\Lambda _{1}(\boldsymbol{\ell })e^{-\hslash _{1}\boldsymbol{\ell }}S(t- \boldsymbol{\ell }) [ \vartheta _{1}V(t-\boldsymbol{\ell })+\vartheta _{2}I(t- \boldsymbol{\ell }) ] \,d\boldsymbol{\ell }- ( \lambda +\gamma ) L(t), \\ \frac{dI(t)}{dt}=\beta \int _{0}^{\kappa _{2}}\Lambda _{2}( \boldsymbol{\ell })e^{-\hslash _{2}\boldsymbol{\ell }}S(t-\boldsymbol{\ell }) [ \vartheta _{1}V(t-\boldsymbol{\ell })+\vartheta _{2}I(t-\boldsymbol{\ell }) ] \,d\boldsymbol{\ell } \\ \hphantom{\frac{dI(t)}{dt}=}{} +\lambda \int _{0}^{\kappa _{3}}\Lambda _{3}(\boldsymbol{\ell })e^{-\hslash _{3}\boldsymbol{\ell }}L(t-\boldsymbol{\ell })\,d\boldsymbol{\ell }-aI(t)-\mu _{1}C^{I}(t)I(t), \\ \frac{dE(t)}{dt}=\varphi \vartheta _{3}\int _{0}^{\kappa _{4}} \Lambda _{4}(\boldsymbol{\ell })e^{-\hslash _{4}\boldsymbol{\ell }}S(t- \boldsymbol{\ell })Y(t-\boldsymbol{\ell })\,d\boldsymbol{\ell }+\mathcal{K}r^{\ast }Y(t)- ( \psi +\omega ) E(t), \\ \frac{dY(t)}{dt}=\psi \int _{0}^{\kappa _{5}}\Lambda _{5}( \boldsymbol{\ell })e^{-\hslash _{5}\boldsymbol{\ell }}E(t-\boldsymbol{\ell })\,d \boldsymbol{\ell }+ ( 1-\mathcal{K} ) r^{\ast }Y(t)-\delta ^{ \ast }Y(t)-\mu _{2}C^{Y}(t)Y(t), \\ \frac{dV(t)}{dt}=b\int _{0}^{\kappa _{6}}\Lambda _{6}( \boldsymbol{\ell })e^{-\hslash _{6}\boldsymbol{\ell }}I(t-\boldsymbol{\ell })\,d \boldsymbol{\ell }-\varepsilon V(t), \\ \frac{dC^{I}(t)}{dt}=\sigma _{1}C^{I}(t)I(t)-\pi _{1}C^{I}(t), \\ \frac{dC^{Y}(t)}{dt}=\sigma _{2}C^{Y}(t)Y(t)-\pi _{2}C^{Y}(t). \end{cases} $$

The factor $\Lambda _{1}(\boldsymbol{\ell })e^{-\hslash _{1} \boldsymbol{\ell }}$ represents the probability that uninfected $\mathrm{CD} 4^{+}\mathrm{T}$ cells contacted by HIV particles or active HIV-infected cells at time $t- \boldsymbol{\ell }$ survived ***ℓ*** time units and become latent infected at time *t*. The term $\Lambda _{2}(\boldsymbol{\ell })e^{-\hslash _{2} \boldsymbol{\ell }}$ is the probability that uninfected $\mathrm{CD} 4^{+}\mathrm{T}$ cells contacted by HIV particles or active HIV-infected cells at time $t-\boldsymbol{\ell }$ survived ***ℓ*** time units and become actively infected at time *t*. The term $\Lambda _{3}(\boldsymbol{\ell })e^{-\hslash _{3}\boldsymbol{\ell }}$ is the probability that latent HIV-infected $\mathrm{CD} 4^{+}\mathrm{T}$ cells survived ***ℓ*** time units before transmitted to be active at time *t*. Moreover, the factor $\Lambda _{4}(\boldsymbol{\ell })e^{-\hslash _{4}\boldsymbol{\ell }}$ demonstrates the probability that the initial infection of uninfected $\mathrm{CD} 4^{+}\mathrm{T}$ cells and the HTLV-infected cells at time $t-\boldsymbol{\ell }$ completing all the intracellular processes that are required for it to become latent HTLV-infected $\mathrm{CD} 4^{+}\mathrm{T}$ cells at time *t*. Further, the probability that latent HTLV-infected $\mathrm{CD} 4^{+}\mathrm{T}$ cells survived ***ℓ*** time units before transmitted to active HTLV-infected cells at time *t* is given by the factor $\Lambda _{5}(\boldsymbol{\ell })e^{-\hslash _{5}\boldsymbol{\ell }}$. Furthermore, the term $\Lambda _{6}(\boldsymbol{\ell })e^{-\hslash _{6} \boldsymbol{\ell }}$ refers to the probability that new immature HIV particles at time $t-\boldsymbol{\ell }$ lost ***ℓ*** time units and become mature at time *t*. Here $\hslash _{i}$, $i=1,2,\ldots,6$, are positive constants. The delay parameter ***ℓ*** is randomly taken from a probability distribution function $\Lambda _{i}(\boldsymbol{\ell })$ over the time interval $[ 0,\kappa _{i} ] $, $i=1,2,\ldots,6$, where $\kappa _{i}$ is the limit superior of this delay period. The function $\Lambda _{i}(\boldsymbol{\ell })$, $i=1,2,\ldots,6$ satisfies $\Lambda _{i}(\boldsymbol{\ell })>0$ and

$$ \int _{0}^{\kappa _{i}}\Lambda _{i}(\boldsymbol{ \ell })\,d \boldsymbol{\ell }=1\quad \text{and}\quad \int _{0}^{\kappa _{i}}\Lambda _{i}( \boldsymbol{ \ell })e^{-u\boldsymbol{\ell }}\,d\boldsymbol{\ell }< \infty , $$

where $u>0$. Let us denote

$$ \bar{\mathcal{H}}_{i}(\boldsymbol{\ell })=\Lambda _{i}( \boldsymbol{\ell })e^{- \hslash _{i}\boldsymbol{\ell }}\quad \text{and}\quad \mathcal{H}_{i}= \int _{0}^{ \kappa _{i}}\bar{\mathcal{H}}_{i}( \boldsymbol{\ell })\,d\boldsymbol{\ell }, $$

where $i=1,2,\ldots,6$. Thus $0<\mathcal{H}_{i}\leq 1$, $i=1,2,\ldots6$.

According to [28], we assume that $r^{\ast }<\min \{ \varrho ,\omega ,\delta ^{\ast } \} $. This yields $\delta ^{\ast }- ( 1-\mathcal{K} ) r^{\ast }>0$. Let $r=\mathcal{K}r^{\ast }$ and $\delta =\delta ^{\ast }- ( 1-\mathcal{K} ) r^{\ast }$. Then system (4) becomes

5 $$ \textstyle\begin{cases}\frac{dS(t)}{dt}=\eta -\varrho S(t)-\vartheta _{1}S(t)V(t)-\vartheta _{2}S(t)I(t)- \vartheta _{3}S(t)Y(t), \\ \frac{dL(t)}{dt}= ( 1-\beta ) \int _{0}^{\kappa _{1}}\bar{\mathcal{H}}_{1}(\boldsymbol{\ell })S(t-\boldsymbol{\ell }) [ \vartheta _{1}V(t-\boldsymbol{\ell })+\vartheta _{2}I(t-\boldsymbol{\ell }) ] \,d\boldsymbol{\ell }- ( \lambda +\gamma ) L(t), \\ \frac{dI(t)}{dt}=\beta \int _{0}^{\kappa _{2}} \bar{\mathcal{H}}_{2}(\boldsymbol{\ell })S(t-\boldsymbol{\ell }) [ \vartheta _{1}V(t- \boldsymbol{\ell })+\vartheta _{2}I(t-\boldsymbol{\ell }) ] \,d \boldsymbol{\ell } \\ \hphantom{\frac{dI(t)}{dt}=}{} +\lambda \int _{0}^{\kappa _{3}}\bar{\mathcal{H}}_{3}(\boldsymbol{\ell })L(t-\boldsymbol{\ell })\,d\boldsymbol{\ell }-aI(t)-\mu _{1}C^{I}(t)I(t), \\ \frac{dE(t)}{dt}=\varphi \vartheta _{3}\int _{0}^{\kappa _{4}}\bar{\mathcal{H}}_{4}(\boldsymbol{\ell })S(t-\boldsymbol{\ell })Y(t- \boldsymbol{\ell })\,d\boldsymbol{\ell }+rY(t)- ( \psi +\omega ) E(t), \\ \frac{dY(t)}{dt}=\psi \int _{0}^{\kappa _{5}} \bar{\mathcal{H}}_{5}(\boldsymbol{\ell })E(t-\boldsymbol{\ell })\,d\boldsymbol{\ell }-\delta Y(t)-\mu _{2}C^{Y}(t)Y(t), \\ \frac{dV(t)}{dt}=b\int _{0}^{\kappa _{6}}\bar{\mathcal{H}}_{6}(\boldsymbol{\ell })I(t-\boldsymbol{\ell })\,d\boldsymbol{\ell }-\varepsilon V(t), \\ \frac{dC^{I}(t)}{dt}=\sigma _{1}C^{I}(t)I(t)-\pi _{1}C^{I}(t), \\ \frac{dC^{Y}(t)}{dt}=\sigma _{2}C^{Y}(t)Y(t)-\pi _{2}C^{Y}(t). \end{cases} $$

The initial conditions of system (5) are given by:

6 $$\begin{aligned} &S(x) =\epsilon _{1}(x), \qquad L(x)=\epsilon _{2}(x), \qquad I(x)= \epsilon _{3}(x), \qquad E(x)=\epsilon _{4}(x), \\ &Y(x) =\epsilon _{5}(x), \qquad V(x)=\epsilon _{6}(x), \qquad C^{I}(x)=\epsilon _{7}(x), \qquad C^{Y}(x)=\epsilon _{8}(x), \\ &\epsilon _{j}(x) \geq 0, \quad x\in {}[ -\kappa ,0], j=1,2,\ldots,8, \kappa =\max \{ \kappa _{1},\kappa _{2},\kappa _{3}, \kappa _{4},\kappa _{5},\kappa _{6}\}, \end{aligned}$$

where $\epsilon _{j}(x)\in \mathcal{C}([-\kappa ,0],\mathbb{R}_{\geq 0})$, $j=1,2,\ldots,8$, and $\mathcal{C}=\mathcal{C}([-\kappa ,0],\mathbb{R}_{\geq 0})$ is the Banach space of continuous functions mapping the interval $[-\kappa ,0]$ into $\mathbb{R}_{\geq 0}$ with norm $\Vert \epsilon _{j}\Vert = [4]\sup _{-\kappa \leq q\leq 0} \vert \epsilon _{j}(q) \vert $ for $\epsilon _{j}\in \mathcal{C}$. Therefore, system (5) with initial conditions (6) has a unique solution by using the standard theory of functional differential equations [61, 62].

## Well-posedness of solutions

### Proposition 1

*All solutions of system* (5) *with initial conditions* (6) *are nonnegative and ultimately bounded*.

### Proof

From the first equation of system (5), we have $\frac{dS(t)}{dt}| _{S=0}=\eta >0$, then $S(t)>0$ for all $t\geq 0$. Moreover, the rest of equations of system (5) give us the following:

$$\begin{aligned} & \begin{aligned} L(t) &=\epsilon _{2}(0)e^{- ( \lambda +\gamma ) t}\\ &\quad {}+ ( 1-\beta ) \int _{0}^{t}e^{- ( \lambda + \gamma ) (t-\varkappa )} \int _{0}^{\kappa _{1}} \bar{\mathcal{H}}_{1}( \boldsymbol{\ell })S(\varkappa -\boldsymbol{\ell }) \bigl[ \vartheta _{1}V(\varkappa -\boldsymbol{\ell })+\vartheta _{2}I( \varkappa -\boldsymbol{\ell }) \bigr] \,d\boldsymbol{\ell }\,d\varkappa , \end{aligned} \\ &\begin{aligned} I(t) & =\epsilon _{3}(0)e^{-\int _{0}^{t} ( a+\mu _{1}C^{I}(y) ) \,dy}\\ &\quad {}+ \int _{0}^{t}e^{-\int _{\varkappa }^{t} ( a+ \mu _{1}C^{I}(y) ) \,dy} \biggl[ \beta \int _{0}^{\kappa _{2}} \bar{\mathcal{H}}_{2}( \boldsymbol{\ell })S(\varkappa -\boldsymbol{\ell }) \bigl[ \vartheta _{1}V(\varkappa -\boldsymbol{\ell })+\vartheta _{2}I(\varkappa -\boldsymbol{\ell }) \bigr] \,d\boldsymbol{\ell } \\ &\quad {} +\lambda \int _{0}^{\kappa _{3}} \bar{\mathcal{H}}_{3}( \boldsymbol{\ell })L(\varkappa -\boldsymbol{\ell })\,d\boldsymbol{\ell } \biggr] \,d \varkappa , \end{aligned} \\ &E(t) =\epsilon _{4}(0)e^{- ( \psi +\omega ) t}+ \int _{0}^{t}e^{- ( \psi +\omega ) (t-\varkappa )} \biggl[ \varphi \vartheta _{3} \int _{0}^{\kappa _{4}} \bar{\mathcal{H}}_{4}( \boldsymbol{\ell })S(\varkappa -\boldsymbol{\ell })Y(\varkappa - \boldsymbol{\ell })\,d\boldsymbol{\ell }+rY(\varkappa ) \biggr] \,d\varkappa , \\ &Y(t) =\epsilon _{5}(0)e^{-\int _{0}^{t} ( \delta +\mu _{2}C^{Y}(y) ) \,dy}+\psi \int _{0}^{t}e^{-\int _{\varkappa }^{t} ( \delta +\mu _{2}C^{Y}(y) ) \,dy} \int _{0}^{ \kappa _{5}}\bar{\mathcal{H}}_{5}( \boldsymbol{\ell })E(\varkappa - \boldsymbol{\ell })\,d\boldsymbol{\ell }\,d\varkappa , \\ &V(t) =\epsilon _{6}(0)e^{-\varepsilon t}+b \int _{0}^{t}e^{-\varepsilon (t-\varkappa )} \int _{0}^{\kappa _{6}} \bar{\mathcal{H}}_{6}( \boldsymbol{\ell })I(\varkappa -\boldsymbol{\ell })\,d\boldsymbol{\ell }\,d \varkappa , \\ &C^{I}(t) =\epsilon _{7}(0)e^{-\int _{0}^{t} ( \pi _{1}-\sigma _{1}I(y) ) \,dy}, \\ &C^{Y}(t) =\epsilon _{8}(0)e^{-\int _{0}^{t} ( \pi _{2}-\sigma _{2}Y(y) ) \,dy}. \end{aligned}$$

Therefore, $L(t),I(t),E(t),Y(t),V(t),C^{I}(t),C^{Y}(t)\geq 0$ for all $t\in [ 0,\kappa ] $. Thus, by a recursive argument, we get $S(t),L(t),I(t),E(t),Y(t),V(t),C^{I}(t),C^{Y}(t)\geq 0$ for all $t\geq 0$. Hence, the solutions of system (5) with initial conditions (6) satisfy $(S(t),L(t),I(t),E(t),Y(t),V(t),C^{I}(t), C^{Y}(t))\in \mathbb{R}_{\geq 0}^{8}$ for all $t\geq 0$. Next, we establish the boundedness of the model’s solutions. The nonnegativity of the model’s solution implies that $\limsup_{t\rightarrow \infty }S(t)\leq \frac{\eta }{\varrho }$. To show the ultimate boundedness of $L(t)$, we let

$$ \Psi _{1}(t)= ( 1-\beta ) \int _{0}^{\kappa _{1}}\bar{\mathcal{H}}_{1}(\boldsymbol{\ell })S(t-\boldsymbol{\ell })\,d\boldsymbol{ \ell }+L(t). $$

Then

$$\begin{aligned} \frac{d\Psi _{1}(t)}{dt} & = ( 1-\beta ) \biggl[ \int _{0}^{\kappa _{1}}\bar{\mathcal{H}}_{1}( \boldsymbol{\ell }) \bigl\{ \eta -\varrho S(t-\boldsymbol{\ell }) \bigr\} \,d \boldsymbol{\ell }- \vartheta _{3}\int _{0}^{\kappa _{1}}\bar{\mathcal{H}}_{1}( \boldsymbol{\ell })S(t-\boldsymbol{\ell })Y(t-\boldsymbol{\ell })\,d \boldsymbol{\ell } \biggr] \\ &\quad {}- ( \lambda +\gamma ) L(t) \\ & = ( 1-\beta ) \biggl[ \eta \mathcal{H}_{1}-\varrho \int _{0}^{\kappa _{1}}\bar{\mathcal{H}}_{1}( \boldsymbol{\ell })S(t- \boldsymbol{\ell })\,d\boldsymbol{\ell }-\vartheta _{3} \int _{0}^{ \kappa _{1}}\bar{\mathcal{H}}_{1}( \boldsymbol{\ell })S(t-\boldsymbol{\ell })Y(t-\boldsymbol{\ell })\,d \boldsymbol{ \ell } \biggr]\\ &\quad {} - ( \lambda +\gamma ) L(t) \\ & \leq \eta \mathcal{H}_{1} ( 1-\beta ) -\varrho ( 1- \beta ) \int _{0}^{\kappa _{1}}\bar{\mathcal{H}}_{1}( \boldsymbol{\ell })S(t-\boldsymbol{\ell })\,d\boldsymbol{\ell }- ( \lambda + \gamma ) L(t) \\ & \leq \eta ( 1-\beta ) -\phi _{1} \biggl[ ( 1- \beta ) \int _{0}^{\kappa _{1}}\bar{\mathcal{H}}_{1}( \boldsymbol{\ell })S(t-\boldsymbol{\ell })\,d\boldsymbol{\ell }+L(t) \biggr] =\eta ( 1-\beta ) -\phi _{1}\Psi _{1}(t), \end{aligned}$$

where $\phi _{1}=\min \{ \varrho ,\lambda +\gamma \}$. It follows that $\limsup_{t\rightarrow \infty }\Psi _{1}(t)\leq \Omega _{1}$, where $\Omega _{1}=\frac{\eta ( 1-\beta ) }{\phi _{1}}$. Since $\int _{0}^{\kappa _{1}}\bar{\mathcal{H}}_{1}(\boldsymbol{\ell })S(t-\boldsymbol{\ell })\,d\boldsymbol{\ell }$ and $L(t)$ are nonnegative, then $\limsup_{t\rightarrow \infty }L(t)\leq \Omega _{1}$. Further, we let

$$ \Psi _{2}(t)=\beta \int _{0}^{\kappa _{2}}\bar{\mathcal{H}}_{2}( \boldsymbol{\ell })S(t-\boldsymbol{\ell })\,d\boldsymbol{\ell }+I(t)+ \frac{\mu _{1}}{\sigma _{1}}C^{I}(t). $$

Then we obtain

$$\begin{aligned} \frac{d\Psi _{2}(t)}{dt} & =\beta \biggl[ \int _{0}^{\kappa _{2}}\bar{\mathcal{H}}_{2}( \boldsymbol{\ell }) \bigl\{ \eta -\varrho S(t- \boldsymbol{\ell }) \bigr\} \,d \boldsymbol{\ell }-\vartheta _{3} \int _{0}^{ \kappa _{2}}\bar{\mathcal{H}}_{2}( \boldsymbol{\ell })S(t-\boldsymbol{\ell })Y(t- \boldsymbol{\ell })\,d\boldsymbol{ \ell } \biggr] \\ &\quad{}+\lambda \int _{0}^{\kappa _{3}}\bar{\mathcal{H}}_{3}( \boldsymbol{\ell })L(t-\boldsymbol{\ell })\,d\boldsymbol{\ell }-aI(t)- \frac{\mu _{1}\pi _{1}}{\sigma _{1}}C^{I}(t) \\ & =\beta \biggl[ \eta \mathcal{H}_{2}-\varrho \int _{0}^{ \kappa _{2}}\bar{\mathcal{H}}_{2}( \boldsymbol{\ell })S(t-\boldsymbol{\ell })\,d \boldsymbol{\ell }-\vartheta _{3} \int _{0}^{\kappa _{2}} \bar{\mathcal{H}}_{2}( \boldsymbol{\ell })S(t-\boldsymbol{\ell })Y(t- \boldsymbol{\ell })\,d\boldsymbol{ \ell } \biggr] \\ &\quad{}+\lambda \int _{0}^{\kappa _{3}}\bar{\mathcal{H}}_{3}( \boldsymbol{\ell })L(t-\boldsymbol{\ell })\,d\boldsymbol{\ell }-aI(t)- \frac{\mu _{1}\pi _{1}}{\sigma _{1}}C^{I}(t) \\ & \leq \eta \beta \mathcal{H}_{2}+\lambda \Omega _{1} \mathcal{H}_{3}- \varrho \beta \int _{0}^{\kappa _{2}}\bar{\mathcal{H}}_{2}( \boldsymbol{\ell })S(t-\boldsymbol{\ell })\,d\boldsymbol{\ell }-aI(t)- \frac{\mu _{1}\pi _{1}}{\sigma _{1}}C^{I}(t) \\ & \leq \eta \beta +\lambda \Omega _{1}-\phi _{2} \biggl[ \beta \int _{0}^{\kappa _{2}}\bar{\mathcal{H}}_{2}(\boldsymbol{\ell })S(t-\boldsymbol{\ell })\,d \boldsymbol{ \ell }+I(t)+\frac{\mu _{1}}{\sigma _{1}}C^{I}(t) \biggr]\\ & = \eta \beta +\lambda \Omega _{1}-\phi _{2}\Psi _{2}(t), \end{aligned}$$

where $\phi _{2}=\min \{ \varrho ,a,\pi _{1}\}$. It follows that $\limsup_{t\rightarrow \infty }\Psi _{2}(t)\leq \Omega _{2}$, where $\Omega _{2}=\frac{\eta \beta +\lambda \Omega _{1}}{\phi _{2}}$. Since $\int _{0}^{\kappa _{2}}\bar{\mathcal{H}}_{2}(\boldsymbol{\ell })S(t-\boldsymbol{\ell })\,d\boldsymbol{\ell }$, $I(t)$ and $C^{I}(t)$ are nonnegative, then $\limsup_{t\rightarrow \infty }I(t)\leq \Omega _{2}$ and $\limsup_{t\rightarrow \infty }C^{I}(t)\leq \Omega _{3}$, where $\Omega _{3}=\frac{\sigma _{1}\Omega _{2}}{\mu _{1}}$. Furthermore, we let

$$ \begin{aligned} \Psi _{3}(t)&= \int _{0}^{\kappa _{4}}\bar{\mathcal{H}}_{4}( \boldsymbol{\ell })S(t-\boldsymbol{\ell })\,d\boldsymbol{\ell }+\frac{1}{\varphi } \bigl[ E(t)+Y(t) \bigr] \\ &\quad {}+\frac{\psi }{\varphi } \int _{0}^{ \kappa _{5}}\Lambda _{5}(\boldsymbol{ \ell }) \int _{t- \boldsymbol{\ell }}^{t}e^{-\hslash _{5} ( t-\varkappa ) }E( \varkappa )\,d \varkappa \,d\boldsymbol{\ell }+ \frac{\mu _{2}}{\sigma _{2}\varphi }C^{Y}(t). \end{aligned} $$

Then

$$\begin{aligned} \frac{d\Psi _{3}(t)}{dt} & = \int _{0}^{\kappa _{4}} \bar{\mathcal{H}}_{4}( \boldsymbol{\ell }) \bigl[ \eta -\varrho S(t-\boldsymbol{\ell })-S(t- \boldsymbol{ \ell }) \bigl\{ \vartheta _{1}V(t-\boldsymbol{\ell })+\vartheta _{2}I(t- \boldsymbol{\ell })+\vartheta _{3}Y(t-\boldsymbol{ \ell }) \bigr\} \bigr] \,d \boldsymbol{\ell } \\ &\quad{}+\vartheta _{3} \int _{0}^{\kappa _{4}}\bar{\mathcal{H}}_{4}( \boldsymbol{\ell })S(t-\boldsymbol{\ell })Y(t-\boldsymbol{\ell })\,d\boldsymbol{\ell }+ \frac{r}{\varphi }Y(t)-\frac{\psi +\omega }{\varphi }E(t) \\ &\quad{}+\frac{\psi }{\varphi } \int _{0}^{\kappa _{5}} \bar{\mathcal{H}}_{5}( \boldsymbol{\ell })E(t-\boldsymbol{\ell })\,d\boldsymbol{\ell }- \frac{\delta }{\varphi }Y(t)\\ &\quad {}-\hslash _{5}\frac{\psi }{\varphi } \int _{0}^{\kappa _{5}}\Lambda _{5}(\boldsymbol{ \ell }) \int _{t- \boldsymbol{\ell }}^{t}e^{-\hslash _{5} ( t-\varkappa ) }E( \varkappa )\,d \varkappa \,d\boldsymbol{\ell } \\ &\quad{}+\frac{\psi }{\varphi }E(t) \int _{0}^{\kappa _{5}}\Lambda _{5}( \boldsymbol{\ell })\,d\boldsymbol{\ell }-\frac{\psi }{\varphi } \int _{0}^{ \kappa _{5}}\Lambda _{5}(\boldsymbol{ \ell })e^{-\hslash _{5}\boldsymbol{\ell }}E(t- \boldsymbol{\ell })\,d\boldsymbol{\ell }- \frac{\mu _{2}\pi _{2}}{\sigma _{2}\varphi }C^{Y}(t) \\ & \leq \int _{0}^{\kappa _{4}}\bar{\mathcal{H}}_{4}( \boldsymbol{\ell }) \bigl[ \eta -\varrho S(t-\boldsymbol{\ell }) \bigr] \,d \boldsymbol{\ell }-\frac{\omega }{\varphi }E(t)+ \biggl( \frac{r}{\varphi }- \frac{\delta }{\varphi } \biggr) Y(t) \\ &\quad{}-\hslash _{5}\frac{\psi }{\varphi } \int _{0}^{\kappa _{5}} \Lambda _{5}(\boldsymbol{ \ell }) \int _{t-\boldsymbol{\ell }}^{t}e^{- \hslash _{5} ( t-\varkappa ) }E(\varkappa )\,d \varkappa \,d \boldsymbol{\ell }-\frac{\mu _{2}\pi _{2}}{\sigma _{2}\varphi }C^{Y}(t) \\ & \leq \eta -\varrho \int _{0}^{\kappa _{4}} \bar{\mathcal{H}}_{4}( \boldsymbol{\ell })S(t-\boldsymbol{\ell })\,d\boldsymbol{\ell }- \frac{\omega }{\varphi }E(t)-\frac{\delta -r}{\varphi }Y(t) \\ &\quad{}-\hslash _{5}\frac{\psi }{\varphi } \int _{0}^{\kappa _{5}} \Lambda _{5}(\boldsymbol{ \ell }) \int _{t-\boldsymbol{\ell }}^{t}e^{- \hslash _{5} ( t-\varkappa ) }E(\varkappa )\,d \varkappa \,d \boldsymbol{\ell }-\frac{\mu _{2}\pi _{2}}{\sigma _{2}\varphi }C^{Y}(t). \end{aligned}$$

Since $\delta -r=\delta ^{\ast }-r^{\ast }>0$, then

$$\begin{aligned} \frac{d\Psi _{3}(t)}{dt} & \leq \eta -\varrho \int _{0}^{ \kappa _{4}}\bar{\mathcal{H}}_{4}( \boldsymbol{\ell })S(t-\boldsymbol{\ell })\,d \boldsymbol{\ell }- \frac{\omega }{\varphi }E(t)-\frac{\delta ^{\ast }-r^{\ast }}{\varphi }Y(t) \\ &\quad{}-\hslash _{5}\frac{\psi }{\varphi } \int _{0}^{\kappa _{5}} \Lambda _{5}(\boldsymbol{ \ell }) \int _{t-\boldsymbol{\ell }}^{t}e^{- \hslash _{5} ( t-\varkappa ) }E(\varkappa )\,d \varkappa \,d \boldsymbol{\ell }-\frac{\mu _{2}\pi _{2}}{\sigma _{2}\varphi }C^{Y}(t) \\ & \leq \eta -\phi _{3} \biggl[ \int _{0}^{\kappa _{4}} \bar{\mathcal{H}}_{4}( \boldsymbol{\ell })S(t-\boldsymbol{\ell })\,d\boldsymbol{\ell }+\frac{1}{\varphi } \bigl\{ E(t)+Y(t) \bigr\} \\ & \quad {} +\frac{\psi }{\varphi } \int _{0}^{\kappa _{5}}\Lambda _{5}( \boldsymbol{\ell }) \int _{t-\boldsymbol{\ell }}^{t}e^{- \hslash _{5} ( t-\varkappa ) }E(\varkappa )\,d \varkappa \,d \boldsymbol{\ell }+\frac{\mu _{2}}{\sigma _{2}\varphi }C^{Y}(t) \biggr] \\ &=\eta -\phi _{3} \Psi _{3}(t), \end{aligned}$$

where $\phi _{3}=\min \{ \varrho ,\omega ,\delta ^{\ast }-r^{\ast },\hslash _{5},\pi _{2}\}$. It follows that $\limsup_{t\rightarrow \infty }\Psi _{3}(t)\leq \frac{\eta }{\phi _{3}}$. Since $\int _{0}^{\kappa _{4}}\bar{\mathcal{H}}_{4}(\boldsymbol{\ell })S(t-\boldsymbol{\ell })\,d\boldsymbol{\ell }\geq 0$, $E(t)\geq 0$, $Y(t)\geq 0$, and $C^{Y}(t)\geq 0$, then $\limsup_{t\rightarrow \infty }E(t)\leq \Omega _{4}$, $\limsup_{t\rightarrow \infty }Y(t)\leq \Omega _{4}$, and $\limsup_{t\rightarrow \infty }C^{Y}(t)\leq \Omega _{5}$, where $\Omega _{4}=\frac{\eta \varphi }{\phi _{3}}$ and $\Omega _{5}=\frac{\eta \sigma _{2}\varphi }{\mu _{2}\phi _{3}}$. Finally, from the sixth equation of system (5), we have

$$ \frac{dV(t)}{dt}=b \int _{0}^{\kappa _{6}}\bar{\mathcal{H}}_{6}( \boldsymbol{\ell })I(t-\boldsymbol{\ell })\,d\boldsymbol{\ell }-\varepsilon V(t)\leq b \mathcal{H}_{6}\Omega _{2}-\varepsilon V(t)\leq b\Omega _{2}- \varepsilon V(t). $$

This implies that $\limsup_{t\rightarrow \infty }V(t)\leq \Omega _{6}$, where $\Omega _{6}=\frac{b\Omega _{2}}{\varepsilon }$. □

According to Proposition 1, we can show that the region

$$\begin{aligned} \Theta & = \bigl\{ \bigl(S,L,I,E,Y,V,C^{I},C^{Y}\bigr)\in \mathcal{C}_{\geq 0}^{8}: \Vert S \Vert \leq \Omega _{1}, \Vert L \Vert \leq \Omega _{1}, \Vert I \Vert \leq \Omega _{2}, \\ &\quad \Vert E \Vert \leq \Omega _{4}, \Vert Y \Vert \leq \Omega _{4}, \Vert V \Vert \leq \Omega _{6}, \bigl\Vert C^{I} \bigr\Vert \leq \Omega _{3}, \bigl\Vert C^{Y} \bigr\Vert \leq \Omega _{5} \bigr\} \end{aligned}$$

is positively invariant with respect to system (5).

## Steady states analysis

In this section, we calculate all possible steady states of the model and derive the threshold parameters which guarantee the existence of the steady states. Let us define

7 $$ \mathcal{P}=\lambda \mathcal{H}_{1}\mathcal{H}_{3} ( 1- \beta ) +\beta \mathcal{H}_{2} ( \gamma +\lambda ) , $$

which will be used throughout the paper. Let $(S,L,I,E,Y,V,C^{I},C^{Y})$ be any steady state of system (5) satisfying the following equations:

$$\begin{aligned} &0 =\eta -\varrho S-\vartheta _{1}SV-\vartheta _{2}SI- \vartheta _{3}SY, \\ &0 =\mathcal{H}_{1} ( 1-\beta ) ( \vartheta _{1}SV+ \vartheta _{2}SI ) - ( \lambda +\gamma ) L, \\ &0 =\beta \mathcal{H}_{2} ( \vartheta _{1}SV+\vartheta _{2}SI ) +\lambda \mathcal{H}_{3}L-aI-\mu _{1}C^{I}I, \\ &0 =\varphi \vartheta _{3}\mathcal{H}_{4}SY+rY- ( \psi + \omega ) E, \\ &0 =\psi \mathcal{H}_{5}E-\delta Y-\mu _{2}C^{Y}Y, \\ &0 =b\mathcal{H}_{6}I-\varepsilon V, \\ &0 = ( \sigma _{1}I-\pi _{1} ) C^{I}, \\ &0 = ( \sigma _{2}Y-\pi _{2} ) C^{Y}. \end{aligned}$$

We find that system (5) has eight possible steady states.

(i) Infection-free steady state, , where $S_{0}=\eta /\varrho $. In this case, the body is free from HTLV and HIV.

(ii) Persistent HIV monoinfection steady state with ineffective immune response, , where

8 $$\begin{aligned} &S_{1} =\frac{S_{0}}{\Re _{1}}, \qquad L_{1}= \frac{a\varepsilon \varrho \mathcal{H}_{1} ( 1-\beta ) }{\mathcal{P} ( b\vartheta _{1}\mathcal{H}_{6}+\varepsilon \vartheta _{2} ) } ( \Re _{1}-1 ) , \\ &I_{1} = \frac{\varepsilon \varrho }{b\vartheta _{1}\mathcal{H}_{6}+\varepsilon \vartheta _{2}} ( \Re _{1}-1 ) , \qquad V_{1}= \frac{\varrho b\mathcal{H}_{6}}{b\vartheta _{1}\mathcal{H}_{6}+\varepsilon \vartheta _{2}} ( \Re _{1}-1 ) , \end{aligned}$$

and $\Re _{1}$ is the basic HIV monoinfection reproduction number for system (5) and is defined as follows:

$$ \Re _{1}= \frac{\mathcal{P}S_{0} ( b\vartheta _{1}\mathcal{H}_{6}+\varepsilon \vartheta _{2} ) }{a\varepsilon ( \gamma +\lambda ) }=\Re _{11}+\Re _{12}, $$

where

$$ \Re _{11}= \frac{\mathcal{P}S_{0}b\vartheta _{1}\mathcal{H}_{6}}{a\varepsilon ( \gamma +\lambda ) }, \qquad \Re _{12}= \frac{\mathcal{P}S_{0}\vartheta _{2}}{a ( \gamma +\lambda ) }. $$

The parameter $\Re _{1}$ determines whether or not a persistent HIV infection can be established. In fact, $\Re _{11}$ measures the average number of secondary HIV infected generation caused by an existing free HIV particle due to free-to-cell transmission, while $\Re _{12}$ measures the average numbers of secondary HIV infected generation caused by living active HIV-infected cells due to infected-to-cell transmission. The steady state  describes the case of persistent HIV monoinfection without immune response.

(iii) Persistent HTLV monoinfection steady state with ineffective immune response, , where

$$ S_{2}=\frac{S_{0}}{\Re _{2}}, \qquad E_{2}= \frac{\varrho \delta }{\vartheta _{3}\psi \mathcal{H}_{5}} ( \Re _{2}-1 ) , \qquad Y_{2}=\frac{\varrho }{\vartheta _{3}} ( \Re _{2}-1 ) , $$

and $\Re _{2}$ is the basic HTLV monoinfection reproduction number for system (5) and is defined as follows:

$$ \Re _{2}= \frac{\varphi \vartheta _{3}\psi \mathcal{H}_{4}\mathcal{H}_{5}S_{0}}{ ( \delta -r\mathcal{H}_{5} ) \psi +\delta \omega }. $$

The parameter $\Re _{2}$ decides whether or not a persistent HTLV infection can be established. The steady state  describes a persistent HTLV monoinfection without immune response.

*We mention that* $\Re _{1}$ *and* $\Re _{2}$ *state the threshold dynamics of infection-free equilibrium*  *and can be calculated by different methods such as (a) the next-generation matrix method of van den Driessche and Watmough [*63*], (b) local stability of the infection-free equilibrium* *, and (c) the existence of the chronic HIV and HTLV monoinfection equilibria with inactive immune response. In the present paper, we derive* $\Re _{1}$ *and* $\Re _{2}$ *by method (c).*

(iv) Persistent HIV monoinfection steady state with only effective HIV-specific CTL, , where

$$\begin{aligned} &S_{3} = \frac{\varepsilon \sigma _{1}\eta }{\pi _{1} ( b\vartheta _{1}\mathcal{H}_{6}+\varepsilon \vartheta _{2} ) +\varrho \varepsilon \sigma _{1}}, \qquad L_{3}= \frac{\eta \pi _{1}\mathcal{H}_{1} ( 1-\beta ) ( b\vartheta _{1}\mathcal{H}_{6}+\varepsilon \vartheta _{2} ) }{ ( \gamma +\lambda ) [ \pi _{1} ( b\vartheta _{1}\mathcal{H}_{6}+\varepsilon \vartheta _{2} ) +\varrho \varepsilon \sigma _{1} ] }, \\ &I_{3} =\frac{\pi _{1}}{\sigma _{1}}, \qquad V_{3}= \frac{b\mathcal{H}_{6}}{\varepsilon }I_{3}= \frac{b\pi _{1}\mathcal{H}_{6}}{\varepsilon \sigma _{1}}, \qquad C_{3}^{I}=\frac{a}{\mu _{1}}(\Re _{3}-1), \end{aligned}$$

and

$$ \Re _{3}= \frac{\sigma _{1}\eta \mathcal{P} ( b\vartheta _{1}\mathcal{H}_{6}+\varepsilon \vartheta _{2} ) }{a ( \gamma +\lambda ) [ \pi _{1} ( b\vartheta _{1}\mathcal{H}_{6}+\varepsilon \vartheta _{2} ) +\varrho \varepsilon \sigma _{1} ] }, $$

is the HIV-specific CTL immunity reproduction number in case of HIV monoinfection. The parameter $\Re _{3}$ determines whether or not the HIV-specific CTL immune response is effective in the absence of HTLV.

(v) Persistent HTLV monoinfection steady state with only effective HTLV-specific CTL, , where

$$\begin{aligned} &S_{4} = \frac{\sigma _{2}\eta }{\pi _{2}\vartheta _{3}+\varrho \sigma _{2}}, \\ &Y_{4} =\frac{\pi _{2}}{\sigma _{2}}, \\ &E_{4} = \frac{\pi _{2} [ r ( \pi _{2}\vartheta _{3}+\varrho \sigma _{2} ) +\vartheta _{3}\eta \varphi \sigma _{2}\mathcal{H}_{4} ] }{\sigma _{2} ( \psi +\omega ) ( \pi _{2}\vartheta _{3}+\varrho \sigma _{2} ) }, \\ &C_{4}^{Y} = \frac{(\delta -r\mathcal{H}_{5})\psi +\delta \omega }{\mu _{2}(\psi +\omega )}(\Re _{4}-1), \end{aligned}$$

and $\Re _{4}$ is the HTLV-specific CTL immunity reproduction number in case of HTLV monoinfection and is stated as follows:

$$ \Re _{4}= \frac{\sigma _{2}\eta \varphi \vartheta _{3}\psi \mathcal{H}_{4}\mathcal{H}_{5}}{ ( \pi _{2}\vartheta _{3}+\varrho \sigma _{2} ) [ (\delta -r\mathcal{H}_{5})\psi +\delta \omega ] }. $$

The parameter $\Re _{4}$ determines whether or not the HTLV-specific CTL immune response is effective in the absence of HIV.

(vi) Persistent HTLV-HIV coinfection steady state with only effective HIV-specific CTL, , where

$$\begin{aligned} &S_{5} = \frac{ ( \delta -r\mathcal{H}_{5} ) \psi +\delta \omega }{\varphi \vartheta _{3}\psi \mathcal{H}_{4}\mathcal{H}_{5}}=S_{2}, \\ &I_{5} =\frac{\pi _{1}}{\sigma _{1}}=I_{3}, \\ &V_{5} =\frac{b\pi _{1}\mathcal{H}_{6}}{\varepsilon \sigma _{1}}=V_{3}, \\ &L_{5} = \frac{\pi _{1}\mathcal{H}_{1} ( 1-\beta ) ( b\vartheta _{1}\mathcal{H}_{6}+\varepsilon \vartheta _{2} ) [ ( \delta -r\mathcal{H}_{5} ) \psi +\delta \omega ] }{\varepsilon \vartheta _{3}\sigma _{1}\varphi \psi \mathcal{H}_{4}\mathcal{H}_{5} ( \gamma +\lambda ) }, \\ &E_{5} = \frac{\delta [ \pi _{1} ( b\vartheta _{1}\mathcal{H}_{6}+\varepsilon \vartheta _{2} ) +\varrho \varepsilon \sigma _{1} ] }{\varepsilon \vartheta _{3}\sigma _{1}\psi \mathcal{H}_{5}} ( \Re _{5}-1 ) , \\ &Y_{5} = \frac{\pi _{1} ( b\vartheta _{1}\mathcal{H}_{6}+\varepsilon \vartheta _{2} ) +\varrho \varepsilon \sigma _{1}}{\varepsilon \vartheta _{3}\sigma _{1}} ( \Re _{5}-1 ) , \\ &C_{5}^{I} =\frac{a}{\mu _{1}} \biggl( \frac{\Re _{1}}{\Re _{2}}-1 \biggr) , \end{aligned}$$

where

$$ \Re _{5}= \frac{\eta \varphi \varepsilon \vartheta _{3}\sigma _{1}\psi \mathcal{H}_{4}\mathcal{H}_{5}}{ [ ( \delta -r\mathcal{H}_{5} ) \psi +\delta \omega ] [ \pi _{1} ( b\vartheta _{1}\mathcal{H}_{6}+\varepsilon \vartheta _{2} ) +\varrho \varepsilon \sigma _{1} ] }. $$

Here, the parameter $\Re _{5}$ is the HTLV infection reproduction number in the presence of HIV infection and determines whether or not HIV-infected patients could be dually infected with HTLV.

(vii) Persistent HTLV-HIV coinfection steady state with only effective HTLV-specific CTL, , where

$$\begin{aligned} &S_{6} = \frac{a\varepsilon ( \gamma +\lambda ) }{\mathcal{P} ( b\vartheta _{1}\mathcal{H}_{6}+\varepsilon \vartheta _{2} ) }=S_{1}, \\ &Y_{6} =\frac{\pi _{2}}{\sigma _{2}}=Y_{4}, \\ &L_{6} = \frac{a\varepsilon \mathcal{H}_{1} ( 1-\beta ) (\pi _{2}\vartheta _{3}+\varrho \sigma _{2})}{\sigma _{2}\mathcal{P} ( b\vartheta _{1}\mathcal{H}_{6}+\varepsilon \vartheta _{2} ) } ( \Re _{6}-1 ) , \\ &I_{6} = \frac{\varepsilon (\pi _{2}\vartheta _{3}+\varrho \sigma _{2})}{\sigma _{2} ( b\vartheta _{1}\mathcal{H}_{6}+\varepsilon \vartheta _{2} ) } ( \Re _{6}-1 ) , \\ &E_{6} = \frac{\pi _{2} [ r\mathcal{P} ( b\vartheta _{1}\mathcal{H}_{6}+\varepsilon \vartheta _{2} ) +a\varepsilon \vartheta _{3}\varphi \mathcal{H}_{4}(\gamma +\lambda ) ] }{\sigma _{2}\mathcal{P}(\psi +\omega ) ( b\vartheta _{1}\mathcal{H}_{6}+\varepsilon \vartheta _{2} ) }, \\ &V_{6} = \frac{b\mathcal{H}_{6}(\pi _{2}\vartheta _{3}+\varrho \sigma _{2})}{\sigma _{2} ( b\vartheta _{1}\mathcal{H}_{6}+\varepsilon \vartheta _{2} ) } ( \Re _{6}-1 ) , \\ &C_{6}^{Y} = \frac{(\delta -r\mathcal{H}_{5})\psi +\delta \omega }{\mu _{2} ( \psi +\omega ) } \biggl( \frac{\Re _{2}}{\Re _{1}}-1 \biggr) , \end{aligned}$$

and

$$ \Re _{6}= \frac{\eta \sigma _{2}\mathcal{P} ( b\vartheta _{1}\mathcal{H}_{6}+\varepsilon \vartheta _{2} ) }{a\varepsilon (\gamma +\lambda )(\pi _{2}\vartheta _{3}+\varrho \sigma _{2})}, $$

is the HIV infection reproduction number in the presence of HTLV infection. It is clear that $\Re _{6}$ determines whether or not HTLV-infected patients could be dually infected with HIV.

(viii) Persistent HTLV-HIV coinfection steady state with effective HIV-specific CTL and HTLV-specific CTL, , where

$$\begin{aligned} &S_{7} = \frac{\varepsilon \sigma _{1}\sigma _{2}\eta }{\pi _{1}\sigma _{2} ( b\vartheta _{1}\mathcal{H}_{6}+\varepsilon \vartheta _{2} ) +\varepsilon \sigma _{1} ( \pi _{2}\vartheta _{3}+\varrho \sigma _{2} ) }, \\ &L_{7} = \frac{\pi _{1}\sigma _{2}\eta \mathcal{H}_{1} ( 1-\beta ) ( b\vartheta _{1}\mathcal{H}_{6}+\varepsilon \vartheta _{2} ) }{ ( \gamma +\lambda ) [ \pi _{1}\sigma _{2} ( b\vartheta _{1}\mathcal{H}_{6}+\varepsilon \vartheta _{2} ) +\varepsilon \sigma _{1} ( \pi _{2}\vartheta _{3}+\varrho \sigma _{2} ) ] }, \\ &E_{7} = \frac{\pi _{2} [ \vartheta _{3}\varepsilon \sigma _{1}\sigma _{2}\eta \varphi \mathcal{H}_{4}+r \{ \pi _{1}\sigma _{2} ( b\vartheta _{1}\mathcal{H}_{6}+\varepsilon \vartheta _{2} ) +\varepsilon \sigma _{1} ( \pi _{2}\vartheta _{3}+\varrho \sigma _{2} ) \} ] }{\sigma _{2} ( \psi +\omega ) [ \pi _{1}\sigma _{2} ( b\vartheta _{1}\mathcal{H}_{6}+\varepsilon \vartheta _{2} ) +\varepsilon \sigma _{1} ( \pi _{2}\vartheta _{3}+\varrho \sigma _{2} ) ] }, \\ &I_{7} =\frac{\pi _{1}}{\sigma _{1}}=I_{3}=I_{5}, \\ &Y_{7} =\frac{\pi _{2}}{\sigma _{2}}=Y_{4}=Y_{6}, \\ &V_{7} =\frac{b\pi _{1}\mathcal{H}_{6}}{\varepsilon \sigma _{1}}=V_{3}=V_{5}, \\ &C_{7}^{I} =\frac{a}{\mu _{1}} ( \Re _{7}-1 ) , \\ &C_{7}^{Y} = \frac{(\delta -r\mathcal{H}_{5})\psi +\delta \omega }{\mu _{2}(\psi +\omega )} ( \Re _{8}-1 ) , \end{aligned}$$

and

$$\begin{aligned} &\Re _{7} = \frac{\sigma _{1}\sigma _{2}\eta \mathcal{P} ( b\vartheta _{1}\mathcal{H}_{6}+\varepsilon \vartheta _{2} ) }{a ( \gamma +\lambda ) [ \pi _{1}\sigma _{2} ( b\vartheta _{1}\mathcal{H}_{6}+\varepsilon \vartheta _{2} ) +\varepsilon \sigma _{1} ( \pi _{2}\vartheta _{3}+\varrho \sigma _{2} ) ] }, \\ &\Re _{8} = \frac{\varepsilon \vartheta _{3}\eta \sigma _{1}\sigma _{2}\varphi \psi \mathcal{H}_{4}\mathcal{H}_{5}}{ [ \pi _{1}\sigma _{2} ( b\vartheta _{1}\mathcal{H}_{6}+\varepsilon \vartheta _{2} ) +\varepsilon \sigma _{1} ( \pi _{2}\vartheta _{3}+\varrho \sigma _{2} ) ] [ (\delta -r\mathcal{H}_{5})\psi +\delta \omega ] }. \end{aligned}$$

The parameter $\Re _{7}$ is the competed HIV-specific CTL immunity reproduction number in case of HTLV-HIV coinfection. The parameter $\Re _{8}$ is the competed HTLV-specific CTL immunity reproduction number in case of HTLV-HIV coinfection. Clearly,  exists when $\Re _{7}>1$ and $\Re _{8}>1$.

## Global stability analysis

In this section, we use the Lyapunov method to show the global asymptotic stability of the model’s steady states. For formation of Lyapunov functionals, we follow the works [64, 65]. Denote $U=U(t)$, where $U\in (S,L,I,E,Y,V,C^{I},C^{Y})$.

Let a function $\Phi _{j}(S,L,I,E,Y,V,C^{I},C^{Y})$ and $\Upsilon _{j}^{{\prime }}$ be the largest invariant subset of

$$ \Upsilon _{j}= \biggl\{ \bigl(S,L,I,E,Y,V,C^{I},C^{Y} \bigr): \frac{d\Phi _{j}}{dt}=0 \biggr\} , \quad j=0,1,2,\ldots,7. $$

We define a function $\digamma (x)=x-1-\ln x$.

### Theorem 1

*If* $\Re _{1}\leq 1$ *and* $\Re _{2}\leq 1$, *then*  *is globally asymptotically stable* (*GAS*).

### Proof

We define a Lyapunov functional as follows:

$$\begin{aligned} \Phi _{0} & =\mathcal{P}S_{0}\digamma \biggl( \frac{S}{S_{0}} \biggr) + \lambda \mathcal{H}_{3}L+ ( \gamma + \lambda ) I+ \frac{\mathcal{P}}{\varphi \mathcal{H}_{4}}E+ \frac{\mathcal{P} ( \psi +\omega ) }{\varphi \psi \mathcal{H}_{4}\mathcal{H}_{5}}Y+ \frac{\mathcal{P}\vartheta _{1}S_{0}}{\varepsilon }V \\ &\quad{}+\frac{\mu _{1} ( \gamma +\lambda ) }{\sigma _{1}}C^{I}+ \frac{\mu _{2}\mathcal{P} ( \psi +\omega ) }{\sigma _{2}\varphi \psi \mathcal{H}_{4}\mathcal{H}_{5}}C^{Y}+ \lambda \mathcal{H}_{3} ( 1-\beta ) \int _{0}^{ \kappa _{1}}\bar{\mathcal{H}}_{1}( \boldsymbol{\ell }) \int _{t-\boldsymbol{\ell }}^{t}S(\varkappa ) \\ &\quad{}\times \bigl[ \vartheta _{1}V(\varkappa )+\vartheta _{2}I( \varkappa ) \bigr] \,d\varkappa \,d\boldsymbol{\ell }\\ &\quad {}+\beta ( \gamma + \lambda ) \int _{0}^{\kappa _{2}}\bar{\mathcal{H}}_{2}( \boldsymbol{\ell }) \int _{t-\boldsymbol{\ell }}^{t}S(\varkappa ) \bigl[ \vartheta _{1}V(\varkappa )+\vartheta _{2}I(\varkappa ) \bigr] \,d \varkappa \,d\boldsymbol{\ell } \\ &\quad{}+\lambda ( \gamma +\lambda ) \int _{0}^{ \kappa _{3}}\bar{\mathcal{H}}_{3}( \boldsymbol{\ell }) \int _{t-\boldsymbol{\ell }}^{t}L(\varkappa )\,d\varkappa \,d \boldsymbol{\ell }+ \frac{\mathcal{P}\vartheta _{3}}{\mathcal{H}_{4}} \int _{0}^{\kappa _{4}}\bar{\mathcal{H}}_{4}( \boldsymbol{\ell }) \int _{t-\boldsymbol{\ell }}^{t}S(\varkappa )Y( \varkappa )\,d\varkappa \,d\boldsymbol{\ell } \\ &\quad{}+ \frac{\mathcal{P} ( \psi +\omega ) }{\varphi \mathcal{H}_{4}\mathcal{H}_{5}} \int _{0}^{\kappa _{5}} \bar{\mathcal{H}}_{5}( \boldsymbol{\ell }) \int _{t-\boldsymbol{\ell }}^{t}E(\varkappa )\,d \varkappa \,d\boldsymbol{ \ell }+ \frac{b\mathcal{P}\vartheta _{1}S_{0}}{\varepsilon }\int _{0}^{\kappa _{6}}\bar{\mathcal{H}}_{6}( \boldsymbol{\ell }) \int _{t-\boldsymbol{\ell }}^{t}I(\varkappa )\,d\varkappa \,d \boldsymbol{ \ell }. \end{aligned}$$

Clearly, $\Phi _{0}(S,L,I,E,Y,V,C^{I},C^{Y})>0$ for all $S,L,I,E,Y,V,C^{I},C^{Y}>0$, and $\Phi _{0}(S_{0},0,0,0,0,0, 0,0)=0$. We calculate $\frac{d\Phi _{0}}{dt}$ along the solutions of model (5) as follows:

9 $$\begin{aligned} \frac{d\Phi _{0}}{dt} & =\mathcal{P} \biggl( 1-\frac{S_{0}}{S} \biggr) ( \eta - \varrho S-\vartheta _{1}SV-\vartheta _{2}SI-\vartheta _{3}SY ) +\lambda \mathcal{H}_{3} \biggl[ ( 1-\beta ) \int _{0}^{\kappa _{1}}\bar{\mathcal{H}}_{1}(\boldsymbol{\ell })S(t-\boldsymbol{\ell }) \\ &\quad {} \times \bigl\{ \vartheta _{1}V(t-\boldsymbol{\ell })+ \vartheta _{2}I(t- \boldsymbol{\ell }) \bigr\} \,d\boldsymbol{\ell }- ( \lambda +\gamma ) L \biggr] + ( \gamma +\lambda ) \biggl[ \beta \int _{0}^{\kappa _{2}}\bar{\mathcal{H}}_{2}(\boldsymbol{\ell })S(t-\boldsymbol{\ell }) \\ & \quad {}\times \bigl\{ \vartheta _{1}V(t-\boldsymbol{\ell })+ \vartheta _{2}I(t- \boldsymbol{\ell }) \bigr\} \,d\boldsymbol{\ell }+ \lambda \int _{0}^{\kappa _{3}}\bar{\mathcal{H}}_{3}(\boldsymbol{\ell })L(t-\boldsymbol{\ell })\,d \boldsymbol{ \ell }-aI-\mu _{1}C^{I}I \biggr] \\ &\quad{}+\frac{\mathcal{P}}{\varphi \mathcal{H}_{4}} \biggl[ \varphi \vartheta _{3}\int _{0}^{\kappa _{4}}\bar{\mathcal{H}}_{4}( \boldsymbol{\ell })S(t-\boldsymbol{\ell })Y(t-\boldsymbol{\ell })\,d \boldsymbol{\ell }+rY- ( \psi + \omega ) E \biggr] \\ &\quad{}+ \frac{\mathcal{P} ( \psi +\omega ) }{\varphi \psi \mathcal{H}_{4}\mathcal{H}_{5}} \biggl[ \psi \int _{0}^{\kappa _{5}} \bar{\mathcal{H}}_{5}( \boldsymbol{\ell })E(t-\boldsymbol{\ell })\,d\boldsymbol{\ell }-\delta Y-\mu _{2}C^{Y}Y \biggr] \\ &\quad{}+\frac{\mathcal{P}\vartheta _{1}S_{0}}{\varepsilon } \biggl[ b \int _{0}^{\kappa _{6}}\bar{\mathcal{H}}_{6}( \boldsymbol{\ell })I(t- \boldsymbol{\ell })\,d\boldsymbol{\ell }-\varepsilon V \biggr] + \frac{\mu _{1} ( \gamma +\lambda ) }{\sigma _{1}} \bigl( \sigma _{1}C^{I}I-\pi _{1}C^{I} \bigr) \\ &\quad{}+ \frac{\mu _{2}\mathcal{P} ( \psi +\omega ) }{\sigma _{2}\varphi \psi \mathcal{H}_{4}\mathcal{H}_{5}} \bigl( \sigma _{2}C^{Y}Y- \pi _{2}C^{Y} \bigr) +\mathcal{P} ( \vartheta _{1}SV+ \vartheta _{2}SI ) -\lambda \mathcal{H}_{3} ( 1-\beta ) \int _{0}^{\kappa _{1}}\bar{\mathcal{H}}_{1}( \boldsymbol{\ell }) \\ &\quad{}\times S(t-\boldsymbol{\ell }) \bigl[ \vartheta _{1}V(t- \boldsymbol{\ell })+\vartheta _{2}I(t-\boldsymbol{\ell }) \bigr] \,d\boldsymbol{\ell }-\beta ( \gamma +\lambda ) \int _{0}^{\kappa _{2}} \bar{\mathcal{H}}_{2}( \boldsymbol{\ell })S(t-\boldsymbol{\ell }) \\ &\quad{}\times \bigl[ \vartheta _{1}V(t-\boldsymbol{\ell })+\vartheta _{2}I(t- \boldsymbol{\ell }) \bigr] \,d\boldsymbol{\ell }+\lambda ( \gamma + \lambda ) \int _{0}^{\kappa _{3}}\bar{\mathcal{H}}_{3}( \boldsymbol{\ell }) \bigl[ L-L(t-\boldsymbol{\ell }) \bigr] \,d\boldsymbol{\ell } \\ &\quad{}+\frac{\mathcal{P}\vartheta _{3}}{\mathcal{H}_{4}} \int _{0}^{\kappa _{4}}\bar{\mathcal{H}}_{4}(\boldsymbol{\ell }) \bigl[ SY-S(t- \boldsymbol{\ell })Y(t- \boldsymbol{\ell }) \bigr] \,d\boldsymbol{\ell }+ \frac{\mathcal{P} ( \psi +\omega ) }{\varphi \mathcal{H}_{4}\mathcal{H}_{5}} \int _{0}^{\kappa _{5}}\bar{\mathcal{H}}_{5}( \boldsymbol{\ell }) \\ &\quad{}\times \bigl[ E-E(t-\boldsymbol{\ell }) \bigr] \,d\boldsymbol{\ell }+ \frac{b\mathcal{P}\vartheta _{1}S_{0}}{\varepsilon } \int _{0}^{ \kappa _{6}}\bar{\mathcal{H}}_{6}( \boldsymbol{\ell }) \bigl[ I-I(t-\boldsymbol{\ell }) \bigr] \,d\boldsymbol{\ell }. \end{aligned}$$

Summing the terms of Eq. (9), we obtain

$$\begin{aligned} \frac{d\Phi _{0}}{dt} & =\mathcal{P} \biggl( 1-\frac{S_{0}}{S} \biggr) ( \eta - \varrho S ) +\mathcal{P}\vartheta _{2}S_{0}I-a ( \lambda + \gamma ) I+ \frac{b\mathcal{P}\vartheta _{1}\mathcal{H}_{6}S_{0}}{\varepsilon }I+\mathcal{P}\vartheta _{3}S_{0}Y \\ &\quad{}- \frac{\mathcal{P} [ ( \delta -r\mathcal{H}_{5} ) \psi +\delta \omega ] }{\varphi \psi \mathcal{H}_{4}\mathcal{H}_{5}}Y- \frac{\mu _{1}\pi _{1} ( \gamma +\lambda ) }{\sigma _{1}}C^{I}- \frac{\mu _{2}\pi _{2}\mathcal{P} ( \psi +\omega ) }{\sigma _{2}\varphi \psi \mathcal{H}_{4}\mathcal{H}_{5}}C^{Y}. \end{aligned}$$

Using $S_{0}=\eta /\varrho $, we obtain

$$\begin{aligned} \frac{d\Phi _{0}}{dt} & =-\varrho \mathcal{P}\frac{(S-S_{0})^{2}}{S}+a ( \lambda +\gamma ) ( \Re _{1}-1 ) I+ \frac{\mathcal{P} [ ( \delta -r\mathcal{H}_{5} ) \psi +\delta \omega ] }{\varphi \psi \mathcal{H}_{4}\mathcal{H}_{5}} ( \Re _{2}-1 ) Y \\ &\quad{}- \frac{\mu _{1}\pi _{1} ( \gamma +\lambda ) }{\sigma _{1}}C^{I}- \frac{\mu _{2}\pi _{2}\mathcal{P} ( \psi +\omega ) }{\sigma _{2}\varphi \psi \mathcal{H}_{4}\mathcal{H}_{5}}C^{Y}. \end{aligned}$$

Since $r<\delta $ and $0<\mathcal{H}_{5}\leq 1$, then $\delta -r\mathcal{H}_{5}>0$. Therefore, $\frac{d\Phi _{0}}{dt}\leq 0$ for all $S,I,Y,C^{I},C^{Y}>0$; moreover, $\frac{d\Phi _{0}}{dt}=0$ when $(S(t),I(t),Y(t),C^{I}(t),C^{Y}(t))=(S_{0},0,0,0,0)$. The solutions of system (5) converge to $\Upsilon _{0}^{{\prime }}$. The set $\Upsilon _{0}^{{\prime }}$ includes elements with $(S(t),I(t),Y(t),C^{I}(t),C^{Y}(t))=(S_{0},0,0,0,0)$. Then $\frac{dS(t)}{dt}=\frac{dY(t)}{dt}=0$ and the first and fifth equations of system (5) become

$$\begin{aligned} &0 =\frac{dS(t)}{dt}=\eta -\varrho S_{0}-\vartheta _{1}S_{0}V(t), \\ &0 =\frac{dY(t)}{dt}=\psi \int _{0}^{\kappa _{5}} \bar{\mathcal{H}}_{5}( \boldsymbol{\ell })E(t-\boldsymbol{\ell })\,d\boldsymbol{\ell }, \end{aligned}$$

which give $V(t)=E(t)=0$ for all *t*. In addition, we have $\frac{dI(t)}{dt}=0$, and from the third equation of system (5) we have

$$ 0=\frac{dI(t)}{dt}=\lambda \int _{0}^{\kappa _{3}} \bar{\mathcal{H}}_{3}( \boldsymbol{\ell })L(t-\boldsymbol{\ell })\,d\boldsymbol{\ell }, $$

which yields $L(t)=0$ for all *t* and hence . Applying Lyapunov–LaSalle asymptotic stability (LLAS) theorem [66–68], we get that  is GAS. □

The following equalities are needed in the next theorems:

10 $$\begin{aligned} & \ln \biggl( \frac{S(t-\boldsymbol{\ell })V(t-\boldsymbol{\ell })}{SV} \biggr) = \biggl[ \ln \biggl( \frac{S(t-\boldsymbol{\ell })V(t-\boldsymbol{\ell })L_{n}}{S_{n}V_{n}L} \biggr) +\ln \biggl( \frac{S_{n}}{S} \biggr) +\ln \biggl( \frac{V_{n}L}{VL_{n}} \biggr) \biggr] , \\ & \ln \biggl( \frac{S(t-\boldsymbol{\ell })V(t-\boldsymbol{\ell })}{SV} \biggr) = \biggl[ \ln \biggl( \frac{S(t-\boldsymbol{\ell })V(t-\boldsymbol{\ell })I_{n}}{S_{n}V_{n}I} \biggr) +\ln \biggl( \frac{S_{n}}{S} \biggr) +\ln \biggl( \frac{V_{n}I}{VI_{n}} \biggr) \biggr] , \\ & \ln \biggl( \frac{S(t-\boldsymbol{\ell })I(t-\boldsymbol{\ell })}{SI} \biggr) = \biggl[ \ln \biggl( \frac{S(t-\boldsymbol{\ell })I(t-\boldsymbol{\ell })L_{n}}{S_{n}I_{n}L} \biggr) +\ln \biggl( \frac{S_{n}}{S} \biggr) +\ln \biggl( \frac{I_{n}L}{IL_{n}} \biggr) \biggr] , \\ & \ln \biggl( \frac{S(t-\boldsymbol{\ell })I(t-\boldsymbol{\ell })}{SI} \biggr) = \biggl[ \ln \biggl( \frac{S(t-\boldsymbol{\ell })I(t-\boldsymbol{\ell })}{S_{n}I} \biggr) +\ln \biggl( \frac{S_{n}}{S} \biggr) \biggr] , \\ & \ln \biggl( \frac{L(t-\boldsymbol{\ell })}{L} \biggr) =\ln \biggl( \frac{L(t-\boldsymbol{\ell })I_{n}}{L_{n}I} \biggr) +\ln \biggl( \frac{L_{n}I}{LI_{n}} \biggr) , \\ & \ln \biggl( \frac{I(t-\boldsymbol{\ell })}{I} \biggr) =\ln \biggl( \frac{I(t-\boldsymbol{\ell })V_{n}}{I_{n}V} \biggr) +\ln \biggl( \frac{I_{n}V}{IV_{n}} \biggr) ,\quad \mbox{where } n=1,3,5,6,7. \end{aligned}$$

Further,

11 $$\begin{aligned} & \ln \biggl( \frac{S(t-\boldsymbol{\ell })Y(t-\boldsymbol{\ell })}{SY} \biggr) =\ln \biggl( \frac{S(t-\boldsymbol{\ell })Y(t-\boldsymbol{\ell })E_{m}}{S_{m}Y_{m}E} \biggr) +\ln \biggl( \frac{S_{m}}{S} \biggr) +\ln \biggl( \frac{Y_{m}E}{YE_{m}} \biggr) , \\ & \ln \biggl( \frac{E(t-\boldsymbol{\ell })}{E} \biggr) =\ln \biggl( \frac{E(t-\boldsymbol{\ell })Y_{m}}{E_{m}Y} \biggr) +\ln \biggl( \frac{YE_{m}}{Y_{m}E} \biggr) ,\quad \text{where }m=2,4,5,6,7. \end{aligned}$$

### Theorem 2

*Let* $\Re _{1}>1$, $\Re _{2}/\Re _{1}\leq 1$, *and* $\Re _{3}\leq 1$, *then*  *is GAS*.

### Proof

Define a functional as follows:

$$\begin{aligned} \Phi _{1} & =\mathcal{P}S_{1}\digamma \biggl( \frac{S}{S_{1}} \biggr) + \lambda \mathcal{H}_{3}L_{1} \digamma \biggl( \frac{L}{L_{1}} \biggr) + ( \gamma +\lambda ) I_{1}\digamma \biggl( \frac{I}{I_{1}} \biggr) + \frac{\mathcal{P}}{\varphi \mathcal{H}_{4}}E+ \frac{\mathcal{P} ( \psi +\omega ) }{\varphi \psi \mathcal{H}_{4}\mathcal{H}_{5}}Y \\ &\quad{}+\frac{\mathcal{P}\vartheta _{1}S_{1}}{\varepsilon }V_{1}\digamma \biggl( \frac{V}{V_{1}} \biggr) + \frac{\mu _{1} ( \gamma +\lambda ) }{\sigma _{1}}C^{I}+ \frac{\mu _{2}\mathcal{P} ( \psi +\omega ) }{\sigma _{2}\varphi \psi \mathcal{H}_{4}\mathcal{H}_{5}}C^{Y} \\ &\quad{}+\vartheta _{1}\lambda \mathcal{H}_{3} ( 1-\beta ) S_{1}V_{1}\int _{0}^{\kappa _{1}}\bar{\mathcal{H}}_{1}( \boldsymbol{\ell }) \int _{t-\boldsymbol{\ell }}^{t}\digamma \biggl( \frac{S(\varkappa )V(\varkappa )}{S_{1}V_{1}} \biggr) \,d\varkappa \,d \boldsymbol{\ell }\\ &\quad {}+\vartheta _{2}\lambda \mathcal{H}_{3} ( 1-\beta ) S_{1}I_{1} \int _{0}^{\kappa _{1}}\bar{\mathcal{H}}_{1}( \boldsymbol{\ell }) \int _{t-\boldsymbol{\ell }}^{t}\digamma \biggl( \frac{S(\varkappa )I(\varkappa )}{S_{1}I_{1}} \biggr) \,d\varkappa \,d \boldsymbol{\ell }\\ &\quad {}+\vartheta _{1}\beta ( \gamma + \lambda ) S_{1}V_{1} \int _{0}^{\kappa _{2}}\bar{\mathcal{H}}_{2}( \boldsymbol{\ell }) \int _{t-\boldsymbol{\ell }}^{t}\digamma \biggl( \frac{S(\varkappa )V(\varkappa )}{S_{1}V_{1}} \biggr) \,d\varkappa \,d \boldsymbol{\ell }\\ &\quad {}+\vartheta _{2}\beta ( \gamma + \lambda ) S_{1}I_{1} \int _{0}^{\kappa _{2}}\bar{\mathcal{H}}_{2}( \boldsymbol{\ell }) \int _{t-\boldsymbol{\ell }}^{t}\digamma \biggl( \frac{S(\varkappa )I(\varkappa )}{S_{1}I_{1}} \biggr) \,d\varkappa \,d\boldsymbol{\ell } \\ &\quad{}+\lambda ( \gamma +\lambda ) L_{1} \int _{0}^{ \kappa _{3}}\bar{\mathcal{H}}_{3}( \boldsymbol{\ell }) \int _{t-\boldsymbol{\ell }}^{t}\digamma \biggl( \frac{L(\varkappa )}{L_{1}} \biggr) \,d\varkappa \,d \boldsymbol{\ell }\\ &\quad {}+\frac{\mathcal{P}\vartheta _{3}}{\mathcal{H}_{4}} \int _{0}^{\kappa _{4}}\bar{\mathcal{H}}_{4}( \boldsymbol{\ell }) \int _{t-\boldsymbol{\ell }}^{t}S(\varkappa )Y(\varkappa )\,d \varkappa \,d\boldsymbol{\ell } \\ &\quad{}+ \frac{\mathcal{P} ( \psi +\omega ) }{\varphi \mathcal{H}_{4}\mathcal{H}_{5}} \int _{0}^{\kappa _{5}} \bar{\mathcal{H}}_{5}( \boldsymbol{\ell }) \int _{t-\boldsymbol{\ell }}^{t}E(\varkappa )\,d \varkappa \,d\boldsymbol{ \ell }\\ &\quad {}+ \frac{b\mathcal{P}\vartheta _{1}S_{1}I_{1}}{\varepsilon }\int _{0}^{\kappa _{6}}\bar{\mathcal{H}}_{6}( \boldsymbol{\ell }) \int _{t-\boldsymbol{\ell }}^{t}\digamma \biggl( \frac{I(\varkappa )}{I_{1}} \biggr) \,d\varkappa \,d\boldsymbol{\ell }. \end{aligned}$$

Calculate $\frac{d\Phi _{1}}{dt}$ as follows:

12 $$\begin{aligned} \frac{d\Phi _{1}}{dt} & =\mathcal{P} \biggl( 1-\frac{S_{1}}{S} \biggr) ( \eta -\varrho S-\vartheta _{1}SV-\vartheta _{2}SI-\vartheta _{3}SY ) +\lambda \mathcal{H}_{3} \biggl( 1- \frac{L_{1}}{L} \biggr) \\ &\quad{}\times \biggl[ ( 1-\beta ) \int _{0}^{\kappa _{1}}\bar{\mathcal{H}}_{1}( \boldsymbol{\ell })S(t-\boldsymbol{\ell }) \bigl\{ \vartheta _{1}V(t- \boldsymbol{\ell })+\vartheta _{2}I(t-\boldsymbol{\ell }) \bigr\} \,d \boldsymbol{\ell }- ( \lambda +\gamma ) L \biggr] \\ &\quad{}+ ( \gamma +\lambda ) \biggl( 1-\frac{I_{1}}{I} \biggr) \biggl[ \beta \int _{0}^{\kappa _{2}}\bar{\mathcal{H}}_{2}( \boldsymbol{\ell })S(t-\boldsymbol{\ell }) \bigl\{ \vartheta _{1}V(t- \boldsymbol{\ell })+\vartheta _{2}I(t-\boldsymbol{\ell }) \bigr\} \,d \boldsymbol{\ell } \\ & \quad{} +\lambda \int _{0}^{\kappa _{3}}\bar{\mathcal{H}}_{3}( \boldsymbol{\ell })L(t-\boldsymbol{\ell })\,d\boldsymbol{\ell }-aI-\mu _{1}C^{I}I \biggr] \\ &\quad {} +\frac{\mathcal{P}}{\varphi \mathcal{H}_{4}} \biggl[ \varphi \vartheta _{3}\int _{0}^{\kappa _{4}}\bar{\mathcal{H}}_{4}( \boldsymbol{\ell })S(t-\boldsymbol{\ell })Y(t-\boldsymbol{\ell })\,d \boldsymbol{\ell } \\ & \quad{} +rY- ( \psi +\omega ) E \biggr] + \frac{\mathcal{P} ( \psi +\omega ) }{\varphi \psi \mathcal{H}_{4}\mathcal{H}_{5}} \biggl[ \psi \int _{0}^{\kappa _{5}}\bar{\mathcal{H}}_{5}( \boldsymbol{\ell })E(t-\boldsymbol{\ell })\,d\boldsymbol{\ell }-\delta Y-\mu _{2}C^{Y}Y \biggr] \\ &\quad{}+\frac{\mathcal{P}\vartheta _{1}S_{1}}{\varepsilon } \biggl( 1- \frac{V_{1}}{V} \biggr) \biggl[ b \int _{0}^{\kappa _{6}} \bar{\mathcal{H}}_{6}( \boldsymbol{\ell })I(t-\boldsymbol{\ell })\,d\boldsymbol{\ell }-\varepsilon V \biggr] \\ &\quad{}+\frac{\mu _{1} ( \gamma +\lambda ) }{\sigma _{1}} \bigl( \sigma _{1}C^{I}I-\pi _{1}C^{I} \bigr) + \frac{\mu _{2}\mathcal{P} ( \psi +\omega ) }{\sigma _{2}\varphi \psi \mathcal{H}_{4}\mathcal{H}_{5}} \bigl( \sigma _{2}C^{Y}Y-\pi _{2}C^{Y} \bigr) \\ &\quad{}+\vartheta _{1}\lambda \mathcal{H}_{3} ( 1-\beta ) S_{1}V_{1} \\ &\quad {}\times \int _{0}^{\kappa _{1}}\bar{\mathcal{H}}_{1}( \boldsymbol{\ell }) \biggl[ \frac{SV}{S_{1}V_{1}}- \frac{S(t-\boldsymbol{\ell })V(t-\boldsymbol{\ell })}{S_{1}V_{1}}+\ln \biggl( \frac{S(t-\boldsymbol{\ell })V(t-\boldsymbol{\ell })}{SV} \biggr) \biggr] \,d\boldsymbol{\ell } \\ &\quad{}+\vartheta _{2}\lambda \mathcal{H}_{3} ( 1-\beta ) S_{1}I_{1} \\ &\quad {}\times\int _{0}^{\kappa _{1}}\bar{\mathcal{H}}_{1}( \boldsymbol{\ell }) \biggl[ \frac{SI}{S_{1}I_{1}}- \frac{S(t-\boldsymbol{\ell })I(t-\boldsymbol{\ell })}{S_{1}I_{1}}+\ln \biggl( \frac{S(t-\boldsymbol{\ell })I(t-\boldsymbol{\ell })}{SI} \biggr) \biggr] \,d\boldsymbol{\ell } \\ &\quad{}+\vartheta _{1}\beta ( \gamma +\lambda ) S_{1}V_{1} \\ &\quad {}\times\int _{0}^{\kappa _{2}}\bar{\mathcal{H}}_{2}( \boldsymbol{\ell }) \biggl[ \frac{SV}{S_{1}V_{1}}- \frac{S(t-\boldsymbol{\ell })V(t-\boldsymbol{\ell })}{S_{1}V_{1}}+ \ln \biggl( \frac{S(t-\boldsymbol{\ell })V(t-\boldsymbol{\ell })}{SV} \biggr) \biggr] \,d\boldsymbol{\ell } \\ &\quad{}+\vartheta _{2}\beta ( \gamma +\lambda ) S_{1}I_{1} \\ &\quad {}\times\int _{0}^{\kappa _{2}}\bar{\mathcal{H}}_{2}( \boldsymbol{\ell }) \biggl[ \frac{SI}{S_{1}I_{1}}- \frac{S(t-\boldsymbol{\ell })I(t-\boldsymbol{\ell })}{S_{1}I_{1}}+ \ln \biggl( \frac{S(t-\boldsymbol{\ell })I(t-\boldsymbol{\ell })}{SI} \biggr) \biggr] \,d\boldsymbol{\ell } \\ &\quad{}+\lambda ( \gamma +\lambda ) L_{1} \int _{0}^{ \kappa _{3}}\bar{\mathcal{H}}_{3}( \boldsymbol{\ell }) \biggl[ \frac{L}{L_{1}}- \frac{L(t-\boldsymbol{\ell })}{L_{1}}+\ln \biggl( \frac{L(t-\boldsymbol{\ell })}{L} \biggr) \biggr] \,d\boldsymbol{\ell } \\ &\quad{}+\frac{\mathcal{P}\vartheta _{3}}{\mathcal{H}_{4}} \int _{0}^{\kappa _{4}}\bar{\mathcal{H}}_{4}(\boldsymbol{\ell }) \bigl[ SY-S(t- \boldsymbol{\ell })Y(t- \boldsymbol{\ell }) \bigr] \,d\boldsymbol{\ell } \\ &\quad {}+ \frac{\mathcal{P} ( \psi +\omega ) }{\varphi \mathcal{H}_{4}\mathcal{H}_{5}} \int _{0}^{\kappa _{5}}\bar{\mathcal{H}}_{5}( \boldsymbol{\ell }) \bigl[ E-E(t- \boldsymbol{\ell }) \bigr] \,d\boldsymbol{\ell } \\ &\quad{}+\frac{b\mathcal{P}\vartheta _{1}S_{1}I_{1}}{\varepsilon } \int _{0}^{\kappa _{6}}\bar{\mathcal{H}}_{6}(\boldsymbol{\ell }) \biggl[ \frac{I}{I_{1}}- \frac{I(t-\boldsymbol{\ell })}{I_{1}}+\ln \biggl( \frac{I(t-\boldsymbol{\ell })}{I} \biggr) \biggr] \,d\boldsymbol{ \ell }. \end{aligned}$$

Summing the terms of Eq. (12), we get

$$\begin{aligned} \frac{d\Phi _{1}}{dt} & =\mathcal{P} \biggl( 1-\frac{S_{1}}{S} \biggr) ( \eta -\varrho S ) +\mathcal{P}\vartheta _{2}S_{1}I+ \mathcal{P}\vartheta _{3}S_{1}Y\\ &\quad {}-\vartheta _{1}\lambda \mathcal{H}_{3} ( 1- \beta ) \int _{0}^{\kappa _{1}}\bar{\mathcal{H}}_{1}( \boldsymbol{\ell })\frac{S(t-\boldsymbol{\ell })V(t-\boldsymbol{\ell })L_{1}}{L}\,d \boldsymbol{\ell } \\ &\quad{}-\vartheta _{2}\lambda \mathcal{H}_{3} ( 1-\beta ) \int _{0}^{\kappa _{1}}\bar{\mathcal{H}}_{1}( \boldsymbol{\ell }) \frac{S(t-\boldsymbol{\ell })I(t-\boldsymbol{\ell })L_{1}}{L}\,d\boldsymbol{\ell }+ \lambda \mathcal{H}_{3} ( \lambda +\gamma ) L_{1}-a ( \lambda + \gamma ) I \\ &\quad{}-\vartheta _{1}\beta ( \gamma +\lambda ) \int _{0}^{\kappa _{2}}\bar{\mathcal{H}}_{2}(\boldsymbol{\ell }) \frac{S(t-\boldsymbol{\ell })V(t-\boldsymbol{\ell })I_{1}}{I}\,d\boldsymbol{\ell }\\ &\quad {}- \vartheta _{2}\beta ( \gamma +\lambda ) \int _{0}^{ \kappa _{2}}\bar{\mathcal{H}}_{2}( \boldsymbol{\ell }) \frac{S(t-\boldsymbol{\ell })I(t-\boldsymbol{\ell })I_{1}}{I}\,d\boldsymbol{\ell } \\ &\quad{}-\lambda ( \lambda +\gamma ) \int _{0}^{ \kappa _{3}}\bar{\mathcal{H}}_{3}( \boldsymbol{\ell }) \frac{L(t-\boldsymbol{\ell })I_{1}}{I}\,d\boldsymbol{\ell }+a ( \lambda +\gamma ) I_{1}\\ &\quad {}+\mu _{1} ( \lambda +\gamma ) C^{I}I_{1}+ \frac{\mathcal{P}r}{\varphi \mathcal{H}_{4}}Y- \frac{\mathcal{P}\delta ( \psi +\omega ) }{\varphi \psi \mathcal{H}_{4}\mathcal{H}_{5}}Y \\ &\quad{}-\frac{\mathcal{P}b\vartheta _{1}S_{1}}{\varepsilon } \int _{0}^{\kappa _{6}}\bar{\mathcal{H}}_{6}(\boldsymbol{\ell }) \frac{I(t-\boldsymbol{\ell })V_{1}}{V}\,d\boldsymbol{\ell }+\mathcal{P}\vartheta _{1}S_{1}V_{1}\\ &\quad {}- \frac{\mu _{1}\pi _{1} ( \lambda +\gamma ) }{\sigma _{1}}C^{I}- \frac{\mu _{2}\pi _{2}\mathcal{P} ( \psi +\omega ) }{\sigma _{2}\varphi \psi \mathcal{H}_{4}\mathcal{H}_{5}}C^{Y} \\ &\quad{}+\lambda \mathcal{H}_{3} ( 1-\beta ) \vartheta _{1}S_{1}V_{1}\int _{0}^{\kappa _{1}}\bar{\mathcal{H}}_{1}( \boldsymbol{\ell }) \ln \biggl( \frac{S(t-\boldsymbol{\ell })V(t-\boldsymbol{\ell })}{SV} \biggr) \,d \boldsymbol{\ell }\\ &\quad {}+\lambda \mathcal{H}_{3} ( 1-\beta ) \vartheta _{2}S_{1}I_{1} \int _{0}^{\kappa _{1}}\bar{\mathcal{H}}_{1}( \boldsymbol{\ell })\ln \biggl( \frac{S(t-\boldsymbol{\ell })I(t-\boldsymbol{\ell })}{SI} \biggr) \,d \boldsymbol{\ell }\\ &\quad {}+\beta ( \gamma +\lambda ) \vartheta _{1}S_{1}V_{1}\int _{0}^{\kappa _{2}}\bar{\mathcal{H}}_{2}( \boldsymbol{\ell }) \ln \biggl( \frac{S(t-\boldsymbol{\ell })V(t-\boldsymbol{\ell })}{SV} \biggr) \,d \boldsymbol{\ell } \\ &\quad{}+\beta ( \gamma +\lambda ) \vartheta _{2}S_{1}I_{1} \int _{0}^{\kappa _{2}}\bar{\mathcal{H}}_{2}( \boldsymbol{\ell }) \ln \biggl( \frac{S(t-\boldsymbol{\ell })I(t-\boldsymbol{\ell })}{SI} \biggr) \,d \boldsymbol{\ell }\\ &\quad {}+\lambda ( \gamma +\lambda ) L_{1} \int _{0}^{ \kappa _{3}}\bar{\mathcal{H}}_{3}( \boldsymbol{\ell })\ln \biggl( \frac{L(t-\boldsymbol{\ell })}{L} \biggr) \,d\boldsymbol{\ell } \\ &\quad{}+ \frac{b\mathcal{P}\vartheta _{1}S_{1}\mathcal{H}_{6}}{\varepsilon }I+\frac{\mathcal{P}b\vartheta _{1}S_{1}I_{1}}{\varepsilon } \int _{0}^{\kappa _{6}}\bar{\mathcal{H}}_{6}(\boldsymbol{\ell })\ln \biggl( \frac{I(t-\boldsymbol{\ell })}{I} \biggr) \,d\boldsymbol{\ell }. \end{aligned}$$

The steady state conditions for  are given by

$$\begin{aligned} & \eta =\varrho S_{1}+\vartheta _{1}S_{1}V_{1}+ \vartheta _{2}S_{1}I_{1}, \qquad \mathcal{H}_{1} ( 1-\beta ) ( \vartheta _{1}S_{1}V_{1}+ \vartheta _{2}S_{1}I_{1} ) = ( \lambda +\gamma ) L_{1}, \\ & \beta \mathcal{H}_{2} ( \vartheta _{1}S_{1}V_{1}+ \vartheta _{2}S_{1}I_{1} ) +\lambda \mathcal{H}_{3}L_{1}=aI_{1} \qquad V_{1}=\frac{b\mathcal{H}_{6}I_{1}}{\varepsilon }. \end{aligned}$$

Then we get

$$ \mathcal{P} ( \vartheta _{1}S_{1}V_{1}+ \vartheta _{2}S_{1}I_{1} ) =a ( \lambda +\gamma ) I_{1}. $$

Further, we obtain

$$\begin{aligned} \frac{d\Phi _{1}}{dt} & =\mathcal{P} \biggl( 1-\frac{S_{1}}{S} \biggr) ( \varrho S_{1}-\varrho S ) +\mathcal{P} ( \vartheta _{1}S_{1}V_{1}+ \vartheta _{2}S_{1}I_{1} ) \biggl( 1- \frac{S_{1}}{S} \biggr) +\mathcal{P}\vartheta _{3}S_{1}Y \\ &\quad{}-\lambda \mathcal{H}_{3} ( 1-\beta ) \vartheta _{1}S_{1}V_{1}\int _{0}^{\kappa _{1}}\bar{\mathcal{H}}_{1}( \boldsymbol{\ell }) \frac{S(t-\boldsymbol{\ell })V(t-\boldsymbol{\ell })L_{1}}{S_{1}V_{1}L}\,d \boldsymbol{\ell }\\ &\quad {}-\lambda \mathcal{H}_{3} ( 1-\beta ) \vartheta _{2}S_{1}I_{1} \int _{0}^{\kappa _{1}}\bar{\mathcal{H}}_{1}( \boldsymbol{\ell })\frac{S(t-\boldsymbol{\ell })I(t-\boldsymbol{\ell })L_{1}}{S_{1}I_{1}L}\,d \boldsymbol{\ell }\\ &\quad {}+\lambda \mathcal{H}_{1}\mathcal{H}_{3} ( 1-\beta ) ( \vartheta _{1}S_{1}V_{1}+\vartheta _{2}S_{1}I_{1} ) \\ &\quad{}-\beta ( \gamma +\lambda ) \vartheta _{1}S_{1}V_{1} \int _{0}^{\kappa _{2}}\bar{\mathcal{H}}_{2}( \boldsymbol{\ell }) \frac{S(t-\boldsymbol{\ell })V(t-\boldsymbol{\ell })I_{1}}{S_{1}V_{1}I}\,d \boldsymbol{\ell }\\ &\quad {}-\beta ( \gamma +\lambda ) \vartheta _{2}S_{1}I_{1} \int _{0}^{\kappa _{2}}\bar{\mathcal{H}}_{2}( \boldsymbol{\ell }) \frac{S(t-\boldsymbol{\ell })I(t-\boldsymbol{\ell })}{S_{1}I}\,d \boldsymbol{\ell }\\ &\quad {}-\lambda \mathcal{H}_{1} ( 1-\beta ) ( \vartheta _{1}S_{1}V_{1}+ \vartheta _{2}S_{1}I_{1} ) \int _{0}^{ \kappa _{3}}\bar{\mathcal{H}}_{3}( \boldsymbol{\ell }) \frac{L(t-\boldsymbol{\ell })I_{1}}{L_{1}I}\,d\boldsymbol{\ell } \\ &\quad{}+\mathcal{P} ( \vartheta _{1}S_{1}V_{1}+ \vartheta _{2}S_{1}I_{1} ) +\mu _{1} ( \lambda +\gamma ) C^{I}I_{1}- \frac{\mathcal{P} [ ( \delta -r\mathcal{H}_{5} ) \psi +\delta \omega ] }{\varphi \psi \mathcal{H}_{4}\mathcal{H}_{5}}Y\\ &\quad {}- \frac{\mathcal{P}\vartheta _{1}S_{1}V_{1}}{\mathcal{H}_{6}} \int _{0}^{\kappa _{6}}\bar{\mathcal{H}}_{6}( \boldsymbol{\ell })\frac{I(t-\boldsymbol{\ell })V_{1}}{I_{1}V}\,d\boldsymbol{\ell }\\ &\quad {}+\mathcal{P} \vartheta _{1}S_{1}V_{1}- \frac{\mu _{1}\pi _{1} ( \lambda +\gamma ) }{\sigma _{1}}C^{I}- \frac{\mu _{2}\pi _{2}\mathcal{P} ( \psi +\omega ) }{\sigma _{2}\varphi \psi \mathcal{H}_{4}\mathcal{H}_{5}}C^{Y} \\ &\quad{}+\lambda \mathcal{H}_{3} ( 1-\beta ) \vartheta _{1}S_{1}V_{1}\int _{0}^{\kappa _{1}}\bar{\mathcal{H}}_{1}( \boldsymbol{\ell }) \ln \biggl( \frac{S(t-\boldsymbol{\ell })V(t-\boldsymbol{\ell })}{SV} \biggr) \,d \boldsymbol{\ell }\\ &\quad{}+\lambda \mathcal{H}_{3} ( 1-\beta ) \vartheta _{2}S_{1}I_{1} \int _{0}^{\kappa _{1}}\bar{\mathcal{H}}_{1}( \boldsymbol{\ell })\ln \biggl( \frac{S(t-\boldsymbol{\ell })I(t-\boldsymbol{\ell })}{SI} \biggr) \,d \boldsymbol{\ell }\\ &\quad {}+\beta ( \gamma +\lambda ) \vartheta _{1}S_{1}V_{1}\int _{0}^{\kappa _{2}}\bar{\mathcal{H}}_{2}( \boldsymbol{\ell }) \ln \biggl( \frac{S(t-\boldsymbol{\ell })V(t-\boldsymbol{\ell })}{SV} \biggr) \,d \boldsymbol{\ell } \\ &\quad{}+\beta ( \gamma +\lambda ) \vartheta _{2}S_{1}I_{1} \int _{0}^{\kappa _{2}}\bar{\mathcal{H}}_{2}( \boldsymbol{\ell }) \ln \biggl( \frac{S(t-\boldsymbol{\ell })I(t-\boldsymbol{\ell })}{SI} \biggr) \,d \boldsymbol{\ell }\\ &\quad {}+\lambda \mathcal{H}_{1} ( 1-\beta ) ( \vartheta _{1}S_{1}V_{1}+\vartheta _{2}S_{1}I_{1} ) \int _{0}^{\kappa _{3}}\bar{\mathcal{H}}_{3}( \boldsymbol{\ell })\ln \biggl( \frac{L(t-\boldsymbol{\ell })}{L} \biggr) \,d \boldsymbol{\ell } \\ &\quad{}+ \frac{\mathcal{P}\vartheta _{1}S_{1}V_{1}}{\mathcal{H}_{6}} \int _{0}^{\kappa _{6}}\bar{\mathcal{H}}_{6}(\boldsymbol{\ell })\ln \biggl( \frac{I(t-\boldsymbol{\ell })}{I} \biggr) \,d\boldsymbol{\ell }. \end{aligned}$$

Using the equalities given by (10) in case of $n=1$, we get

13 $$\begin{aligned} \frac{d\Phi _{1}}{dt} & =-\varrho \mathcal{P} \frac{(S-S_{1})^{2}}{S}-\mathcal{P} ( \vartheta _{1}S_{1}V_{1}+\vartheta _{2}S_{1}I_{1} ) \biggl[ \frac{S_{1}}{S}-1- \ln \biggl( \frac{S_{1}}{S} \biggr) \biggr] \\ &\quad{}-\lambda \mathcal{H}_{3} ( 1-\beta ) \vartheta _{1}S_{1}V_{1} \\ &\quad {}\times\int _{0}^{\kappa _{1}}\bar{\mathcal{H}}_{1}( \boldsymbol{\ell }) \biggl[ \frac{S(t-\boldsymbol{\ell })V(t-\boldsymbol{\ell })L_{1}}{S_{1}V_{1}L}-1- \ln \biggl( \frac{S(t-\boldsymbol{\ell })V(t-\boldsymbol{\ell })L_{1}}{S_{1}V_{1}L} \biggr) \biggr] \,d\boldsymbol{\ell } \\ &\quad{}-\lambda \mathcal{H}_{3} ( 1-\beta ) \vartheta _{2}S_{1}I_{1} \\ &\quad {}\times\int _{0}^{\kappa _{1}}\bar{\mathcal{H}}_{1}( \boldsymbol{\ell }) \biggl[ \frac{S(t-\boldsymbol{\ell })I(t-\boldsymbol{\ell })L_{1}}{S_{1}I_{1}L}-1- \ln \biggl( \frac{S(t-\boldsymbol{\ell })I(t-\boldsymbol{\ell })L_{1}}{S_{1}I_{1}L} \biggr) \biggr] \,d\boldsymbol{\ell } \\ &\quad{}-\beta ( \gamma +\lambda ) \vartheta _{1}S_{1}V_{1} \\ &\quad {}\times\int _{0}^{\kappa _{2}}\bar{\mathcal{H}}_{2}( \boldsymbol{\ell }) \biggl[ \frac{S(t-\boldsymbol{\ell })V(t-\boldsymbol{\ell })I_{1}}{S_{1}V_{1}I}-1- \ln \biggl( \frac{S(t-\boldsymbol{\ell })V(t-\boldsymbol{\ell })I_{1}}{S_{1}V_{1}I} \biggr) \biggr] \,d\boldsymbol{\ell } \\ &\quad{}-\beta ( \gamma +\lambda ) \vartheta _{2}S_{1}I_{1} \int _{0}^{\kappa _{2}}\bar{\mathcal{H}}_{2}( \boldsymbol{\ell }) \biggl[ \frac{S(t-\boldsymbol{\ell })I(t-\boldsymbol{\ell })}{S_{1}I}-1-\ln \biggl( \frac{S(t-\boldsymbol{\ell })I(t-\boldsymbol{\ell })}{S_{1}I} \biggr) \biggr] \,d\boldsymbol{\ell } \\ &\quad{}-\lambda \mathcal{H}_{1} ( 1-\beta ) ( \vartheta _{1}S_{1}V_{1}+\vartheta _{2}S_{1}I_{1} ) \\ &\quad {}\times\int _{0}^{ \kappa _{3}}\bar{\mathcal{H}}_{3}( \boldsymbol{\ell }) \biggl[ \frac{L(t-\boldsymbol{\ell })I_{1}}{L_{1}I}-1-\ln \biggl( \frac{L(t-\boldsymbol{\ell })I_{1}}{L_{1}I} \biggr) \biggr] \,d\boldsymbol{\ell } \\ &\quad{}-\frac{\mathcal{P}\vartheta _{1}S_{1}V_{1}}{\mathcal{H}_{6}} \int _{0}^{\kappa _{6}}\bar{\mathcal{H}}_{6}( \boldsymbol{\ell }) \biggl[ \frac{I(t-\boldsymbol{\ell })V_{1}}{I_{1}V}-1-\ln \biggl( \frac{I(t-\boldsymbol{\ell })V_{1}}{I_{1}V} \biggr) \biggr] \,d \boldsymbol{\ell } \\ &\quad {}+ \frac{\mathcal{P} [ ( \delta -r\mathcal{H}_{5} ) \psi +\delta \omega ] }{\varphi \psi \mathcal{H}_{4}\mathcal{H}_{5}}\biggl( \frac{\vartheta _{3}\varphi \psi \mathcal{H}_{4}\mathcal{H}_{5}S_{1}}{ ( \delta -r\mathcal{H}_{5} ) \psi +\delta \omega }-1 \biggr) Y \\ &\quad{}+\mu _{1} ( \lambda +\gamma ) \biggl( I_{1}- \frac{\pi _{1}}{\sigma _{1}} \biggr) C^{I}- \frac{\mu _{2}\pi _{2}\mathcal{P} ( \psi +\omega ) }{\sigma _{2}\varphi \psi \mathcal{H}_{4}\mathcal{H}_{5}}C^{Y}. \end{aligned}$$

Therefore, Eq. (13) becomes

$$\begin{aligned} \frac{d\Phi _{1}}{dt} & =-\varrho \mathcal{P} \frac{(S-S_{1})^{2}}{S}-\mathcal{P} ( \vartheta _{1}S_{1}V_{1}+\vartheta _{2}S_{1}I_{1} ) \digamma \biggl( \frac{S_{1}}{S} \biggr) \\ &\quad{}-\lambda \mathcal{H}_{3} ( 1-\beta ) \vartheta _{1}S_{1}V_{1}\int _{0}^{\kappa _{1}}\bar{\mathcal{H}}_{1}( \boldsymbol{\ell }) \digamma \biggl( \frac{S(t-\boldsymbol{\ell })V(t-\boldsymbol{\ell })L_{1}}{S_{1}V_{1}L} \biggr) \,d \boldsymbol{ \ell } \\ &\quad{}-\lambda \mathcal{H}_{3} ( 1-\beta ) \vartheta _{2}S_{1}I_{1}\int _{0}^{\kappa _{1}}\bar{\mathcal{H}}_{1}( \boldsymbol{\ell }) \digamma \biggl( \frac{S(t-\boldsymbol{\ell })I(t-\boldsymbol{\ell })L_{1}}{S_{1}I_{1}L} \biggr) \,d \boldsymbol{ \ell } \\ &\quad{}-\beta ( \gamma +\lambda ) \vartheta _{1}S_{1}V_{1} \int _{0}^{\kappa _{2}}\bar{\mathcal{H}}_{2}( \boldsymbol{\ell }) \digamma \biggl( \frac{S(t-\boldsymbol{\ell })V(t-\boldsymbol{\ell })I_{1}}{S_{1}V_{1}I} \biggr) \,d \boldsymbol{ \ell } \\ &\quad{}-\beta ( \gamma +\lambda ) \vartheta _{2}S_{1}I_{1} \int _{0}^{\kappa _{2}}\bar{\mathcal{H}}_{2}( \boldsymbol{\ell }) \digamma \biggl( \frac{S(t-\boldsymbol{\ell })I(t-\boldsymbol{\ell })}{S_{1}I} \biggr) \,d\boldsymbol{\ell } \\ &\quad{}-\lambda \mathcal{H}_{1} ( 1-\beta ) ( \vartheta _{1}S_{1}V_{1}+\vartheta _{2}S_{1}I_{1} ) \int _{0}^{ \kappa _{3}}\bar{\mathcal{H}}_{3}( \boldsymbol{\ell })\digamma \biggl( \frac{L(t-\boldsymbol{\ell })I_{1}}{L_{1}I} \biggr) \,d\boldsymbol{\ell } \\ &\quad{}-\frac{\mathcal{P}\vartheta _{1}S_{1}V_{1}}{\mathcal{H}_{6}} \int _{0}^{\kappa _{6}}\bar{\mathcal{H}}_{6}( \boldsymbol{\ell }) \digamma \biggl( \frac{I(t-\boldsymbol{\ell })V_{1}}{I_{1}V} \biggr) \,d \boldsymbol{ \ell }+ \frac{\mathcal{P} [ ( \delta -r\mathcal{H}_{5} ) \psi +\delta \omega ] }{\varphi \psi \mathcal{H}_{4}\mathcal{H}_{5}} \biggl( \frac{\Re _{2}}{\Re _{1}}-1 \biggr) Y \\ &\quad{}+ \frac{\mu _{1} ( \gamma +\lambda ) [ \pi _{1} ( b\vartheta _{1}\mathcal{H}_{6}+\varepsilon \vartheta _{2} ) +\varrho \varepsilon \sigma _{1} ] }{\sigma _{1} ( b\vartheta _{1}\mathcal{H}_{6}+\varepsilon \vartheta _{2} ) } ( \Re _{3}-1 ) C^{I}- \frac{\mu _{2}\pi _{2}\mathcal{P} ( \psi +\omega ) }{\sigma _{2}\varphi \psi \mathcal{H}_{4}\mathcal{H}_{5}}C^{Y}. \end{aligned}$$

Since $\Re _{2}/\Re _{1}\leq 1$ and $\Re _{3}\leq 1$, then $\frac{d\Phi _{1}}{dt}\leq 0$ for all $S,L,I,Y,V,C^{I},C^{Y}>0$. Moreover, $\frac{d\Phi _{1}}{dt}=0$ when $S=S_{1}$ and $Y=C^{I}=C^{Y}=\digamma =0$. The solutions of system (5) converge to $\Upsilon _{1}^{{\prime }}$ which includes elements that satisfy $S(t)=S_{1}$ and $\digamma =0$ i.e.

14 $$\begin{aligned} \frac{S(t-\boldsymbol{\ell })V(t-\boldsymbol{\ell })L_{1}}{S_{1}V_{1}L} & = \frac{S(t-\boldsymbol{\ell })I(t-\boldsymbol{\ell })L_{1}}{S_{1}I_{1}L}= \frac{S(t-\boldsymbol{\ell })V(t-\boldsymbol{\ell })I_{1}}{S_{1}V_{1}I} \\ & =\frac{S(t-\boldsymbol{\ell })I(t-\boldsymbol{\ell })}{S_{1}I}= \frac{L(t-\boldsymbol{\ell })I_{1}}{L_{1}I}= \frac{I(t-\boldsymbol{\ell })V_{1}}{I_{1}V}=1 \end{aligned}$$

for all $t\in {}[ 0,\kappa ]$. If $S(t)=S_{1}$, then from Eq. (14) we get $L(t)=L_{1}$, $I(t)=I_{1}$, and $V(t)=V_{1}$ for all *t*. Further, for each element of $\Upsilon _{1}^{\prime }$, we have $Y(t)=0$ and then $\frac{dY(t)}{dt}=0$. The fifth equation of system (5) becomes

$$ 0=\frac{dY(t)}{dt}=\psi \int _{0}^{\kappa _{5}} \bar{\mathcal{H}}_{5}( \boldsymbol{\ell })E(t-\boldsymbol{\ell })\,d\boldsymbol{\ell }, $$

which provides $E(t)=0$ for all *t*, and hence . Therefore, using LLAS theorem we get that  is GAS. □

### Theorem 3

*If* $\Re _{2}>1$, $\Re _{1}/\Re _{2}\leq 1$, *and* $\Re _{4}\leq 1$, *then*  *is GAS*.

### Proof

Define

$$\begin{aligned} \Phi _{2} & =\mathcal{P}S_{2}\digamma \biggl( \frac{S}{S_{2}} \biggr) + \lambda \mathcal{H}_{3}L+ ( \gamma + \lambda ) I+ \frac{\mathcal{P}}{\varphi \mathcal{H}_{4}}E_{2}\digamma \biggl( \frac{E}{E_{2}} \biggr) + \frac{\mathcal{P} ( \psi +\omega ) }{\varphi \psi \mathcal{H}_{4}\mathcal{H}_{5}}Y_{2}\digamma \biggl( \frac{Y}{Y_{2}} \biggr) \\ &\quad{}+\frac{\mathcal{P}\vartheta _{1}S_{2}}{\varepsilon }V+ \frac{\mu _{1} ( \gamma +\lambda ) }{\sigma _{1}}C^{I}+ \frac{\mu _{2}\mathcal{P} ( \psi +\omega ) }{\sigma _{2}\varphi \psi \mathcal{H}_{4}\mathcal{H}_{5}}C^{Y}+\lambda \mathcal{H}_{3} ( 1-\beta ) \int _{0}^{\kappa _{1}}\bar{\mathcal{H}}_{1}(\boldsymbol{\ell }) \int _{t- \boldsymbol{\ell }}^{t}S(\varkappa ) \\ &\quad{}\times \bigl[ \vartheta _{1}V(\varkappa )+\vartheta _{2}I( \varkappa ) \bigr] \,d\varkappa \,d\boldsymbol{\ell }\\ &\quad {}+\beta ( \gamma + \lambda ) \int _{0}^{\kappa _{2}}\bar{\mathcal{H}}_{2}( \boldsymbol{\ell }) \int _{t-\boldsymbol{\ell }}^{t}S(\varkappa ) \bigl[ \vartheta _{1}V(\varkappa )+\vartheta _{2}I(\varkappa ) \bigr] \,d \varkappa \,d\boldsymbol{\ell } \\ &\quad{}+\lambda ( \gamma +\lambda ) \int _{0}^{ \kappa _{3}}\bar{\mathcal{H}}_{3}( \boldsymbol{\ell }) \int _{t-\boldsymbol{\ell }}^{t}L(\varkappa )\,d\varkappa \,d \boldsymbol{\ell }\\ &\quad {}+ \frac{\mathcal{P}\vartheta _{3}S_{2}Y_{2}}{\mathcal{H}_{4}} \int _{0}^{\kappa _{4}} \bar{\mathcal{H}}_{4}( \boldsymbol{\ell }) \int _{t-\boldsymbol{\ell }}^{t}\digamma \biggl( \frac{S(\varkappa )Y(\varkappa )}{S_{2}Y_{2}} \biggr) \,d\varkappa \,d \boldsymbol{\ell } \\ &\quad{}+ \frac{\mathcal{P} ( \psi +\omega ) E_{2}}{\varphi \mathcal{H}_{4}\mathcal{H}_{5}} \int _{0}^{\kappa _{5}} \bar{\mathcal{H}}_{5}( \boldsymbol{\ell }) \int _{t-\boldsymbol{\ell }}^{t}\digamma \biggl( \frac{E(\varkappa )}{E_{2}} \biggr) \,d\varkappa \,d\boldsymbol{\ell }\\ &\quad {}+ \frac{b\mathcal{P}\vartheta _{1}S_{2}}{\varepsilon } \int _{0}^{ \kappa _{6}}\bar{\mathcal{H}}_{6}( \boldsymbol{\ell }) \int _{t-\boldsymbol{\ell }}^{t}I(\varkappa )\,d\varkappa \,d \boldsymbol{\ell }. \end{aligned}$$

We calculate $\frac{d\Phi _{2}}{dt}$ as follows:

15 $$\begin{aligned} \frac{d\Phi _{2}}{dt} & =\mathcal{P} \biggl( 1-\frac{S_{2}}{S} \biggr) ( \eta -\varrho S-\vartheta _{1}SV-\vartheta _{2}SI-\vartheta _{3}SY ) \\ &\quad{}+\lambda \mathcal{H}_{3} \biggl[ ( 1-\beta ) \int _{0}^{\kappa _{1}}\bar{\mathcal{H}}_{1}(\boldsymbol{\ell })S(t-\boldsymbol{\ell }) \bigl\{ \vartheta _{1}V(t-\boldsymbol{\ell })+\vartheta _{2}I(t- \boldsymbol{\ell }) \bigr\} \,d\boldsymbol{\ell }- ( \lambda +\gamma ) L \biggr] \\ &\quad{}+ ( \gamma +\lambda ) \biggl[ \beta \int _{0}^{ \kappa _{2}}\bar{\mathcal{H}}_{2}( \boldsymbol{\ell })S(t-\boldsymbol{\ell }) \bigl\{ \vartheta _{1}V(t- \boldsymbol{\ell })+\vartheta _{2}I(t-\boldsymbol{\ell }) \bigr\} \,d \boldsymbol{\ell } \\ &\quad {}+\lambda \int _{0}^{\kappa _{3}} \bar{\mathcal{H}}_{3}( \boldsymbol{\ell })L(t-\boldsymbol{\ell })\,d\boldsymbol{\ell } -aI-\mu _{1}C^{I}I \biggr] \\ & \quad{} + \frac{\mathcal{P}}{\varphi \mathcal{H}_{4}} \biggl( 1-\frac{E_{2}}{E} \biggr) \biggl[ \varphi \vartheta _{3} \int _{0}^{\kappa _{4}} \bar{\mathcal{H}}_{4}( \boldsymbol{\ell })S(t-\boldsymbol{\ell })Y(t- \boldsymbol{\ell })\,d\boldsymbol{ \ell }+rY- ( \psi +\omega ) E \biggr] \\ &\quad{}+ \frac{\mathcal{P} ( \psi +\omega ) }{\varphi \psi \mathcal{H}_{4}\mathcal{H}_{5}} \biggl( 1-\frac{Y_{2}}{Y} \biggr) \biggl[ \psi \int _{0}^{\kappa _{5}}\bar{\mathcal{H}}_{5}( \boldsymbol{\ell })E(t-\boldsymbol{\ell })\,d\boldsymbol{\ell }-\delta Y- \mu _{2}C^{Y}Y \biggr] + \frac{\mathcal{P}\vartheta _{1}S_{2}}{\varepsilon } \\ &\quad{}\times \biggl[ b \int _{0}^{\kappa _{6}}\bar{\mathcal{H}}_{6}( \boldsymbol{\ell })I(t-\boldsymbol{\ell })\,d\boldsymbol{\ell }-\varepsilon V \biggr] + \frac{\mu _{1} ( \gamma +\lambda ) }{\sigma _{1}} \bigl( \sigma _{1}C^{I}I-\pi _{1}C^{I} \bigr) + \frac{\mu _{2}\mathcal{P} ( \psi +\omega ) }{\sigma _{2}\varphi \psi \mathcal{H}_{4}\mathcal{H}_{5}} \\ &\quad{}\times \bigl( \sigma _{2}C^{Y}Y-\pi _{2}C^{Y} \bigr) +\mathcal{P} ( \vartheta _{1}SV+ \vartheta _{2}SI ) -\lambda \mathcal{H}_{3} ( 1-\beta ) \int _{0}^{\kappa _{1}} \bar{\mathcal{H}}_{1}( \boldsymbol{\ell })S(t-\boldsymbol{\ell }) \\ &\quad{}\times \bigl[ \vartheta _{1}V(t-\boldsymbol{\ell })+\vartheta _{2}I(t- \boldsymbol{\ell }) \bigr] \,d\boldsymbol{\ell }-\beta ( \gamma +\lambda ) \int _{0}^{\kappa _{2}}\bar{\mathcal{H}}_{2}( \boldsymbol{\ell })S(t-\boldsymbol{\ell }) \bigl[ \vartheta _{1}V(t- \boldsymbol{\ell }) \\ & \quad{} +\vartheta _{2}I(t-\boldsymbol{\ell }) \bigr] \,d \boldsymbol{\ell }+\lambda ( \gamma +\lambda ) \int _{0}^{\kappa _{3}}\bar{\mathcal{H}}_{3}( \boldsymbol{\ell }) \bigl[ L-L(t-\boldsymbol{\ell }) \bigr] \,d\boldsymbol{\ell } \\ &\quad {}+ \frac{\mathcal{P}\vartheta _{3}S_{2}Y_{2}}{\mathcal{H}_{4}}\int _{0}^{\kappa _{4}}\bar{\mathcal{H}}_{4}( \boldsymbol{\ell }) \biggl[ \frac{SY}{S_{2}Y_{2}} \\ & \quad{} -\frac{S(t-\boldsymbol{\ell })Y(t-\boldsymbol{\ell })}{S_{2}Y_{2}}+ \ln \biggl( \frac{S(t-\boldsymbol{\ell })Y(t-\boldsymbol{\ell })}{SY} \biggr) \biggr] \,d\boldsymbol{\ell } \\ &\quad{}+\frac{\mathcal{P} ( \psi +\omega ) E_{2}}{\varphi \mathcal{H}_{4}\mathcal{H}_{5}} \int _{0}^{\kappa _{5}}\bar{\mathcal{H}}_{5}( \boldsymbol{\ell }) \biggl[ \frac{E}{E_{2}}-\frac{E(t-\boldsymbol{\ell })}{E_{2}}+ \ln \biggl( \frac{E(t-\boldsymbol{\ell })}{E} \biggr) \biggr] \,d \boldsymbol{\ell } \\ &\quad {}+\frac{b\mathcal{P}\vartheta _{1}S_{2}}{\varepsilon } \int _{0}^{\kappa _{6}}\bar{\mathcal{H}}_{6}( \boldsymbol{\ell }) \bigl[ I-I(t-\boldsymbol{\ell }) \bigr] \,d\boldsymbol{\ell }. \end{aligned}$$

By collecting the terms of Eq. (15), we get

$$\begin{aligned} \frac{d\Phi _{2}}{dt} & =\mathcal{P} \biggl[ \biggl( 1-\frac{S_{2}}{S} \biggr) ( \eta -\varrho S ) +\vartheta _{2}S_{2}I+ \vartheta _{3}S_{2}Y-\frac{a ( \lambda +\gamma ) }{\mathcal{P}}I+ \frac{r}{\varphi \mathcal{H}_{4}}Y \\ &\quad {} -\frac{\vartheta _{3}}{\mathcal{H}_{4}} \int _{0}^{ \kappa _{4}}\bar{\mathcal{H}}_{4}( \boldsymbol{\ell }) \frac{S(t-\boldsymbol{\ell })Y(t-\boldsymbol{\ell })E_{2}}{E}\,d\boldsymbol{\ell }- \frac{r}{\varphi \mathcal{H}_{4}} \frac{YE_{2}}{E}+\frac{\psi +\omega }{\varphi \mathcal{H}_{4}}E_{2}- \frac{\delta ( \psi +\omega ) }{\varphi \psi \mathcal{H}_{4}\mathcal{H}_{5}}Y \\ &\quad {} - \frac{\psi +\omega }{\varphi \mathcal{H}_{4}\mathcal{H}_{5}}\int _{0}^{\kappa _{5}}\bar{\mathcal{H}}_{5}( \boldsymbol{\ell }) \frac{E(t-\boldsymbol{\ell })Y_{2}}{Y}\,d\boldsymbol{\ell }+ \frac{\delta ( \psi +\omega ) }{\varphi \psi \mathcal{H}_{4}\mathcal{H}_{5}}Y_{2}+ \frac{\mu _{2} ( \psi +\omega ) }{\varphi \psi \mathcal{H}_{4}\mathcal{H}_{5}}C^{Y}Y_{2} \\ &\quad {} - \frac{\mu _{1}\pi _{1} ( \gamma +\lambda ) }{\sigma _{1}\mathcal{P}}C^{I}- \frac{\mu _{2}\pi _{2} ( \psi +\omega ) }{\sigma _{2}\varphi \psi \mathcal{H}_{4}\mathcal{H}_{5}}C^{Y}\\ &\quad {}+ \frac{\vartheta _{3}S_{2}Y_{2}}{\mathcal{H}_{4}} \int _{0}^{ \kappa _{4}}\bar{\mathcal{H}}_{4}( \boldsymbol{\ell })\ln \biggl( \frac{S(t-\boldsymbol{\ell })Y(t-\boldsymbol{\ell })}{SY} \biggr) \,d\boldsymbol{\ell } \\ & \quad{} + \frac{ ( \psi +\omega ) E_{2}}{\varphi \mathcal{H}_{4}\mathcal{H}_{5}} \int _{0}^{\kappa _{5}} \bar{\mathcal{H}}_{5}( \boldsymbol{\ell })\ln \biggl( \frac{E(t-\boldsymbol{\ell })}{E} \biggr) \,d \boldsymbol{\ell }+ \frac{b\vartheta _{1}S_{2}\mathcal{H}_{6}}{\varepsilon }I \biggr] . \end{aligned}$$

Using the steady state conditions for

$$ \eta =\varrho S_{2}+\vartheta _{3}S_{2}Y_{2}, \qquad \vartheta _{3}S_{2}Y_{2}+ \frac{rY_{2}}{\varphi \mathcal{H}_{4}}= \frac{ ( \psi +\omega ) E_{2}}{\varphi \mathcal{H}_{4}}= \frac{\delta ( \psi +\omega ) Y_{2}}{\varphi \psi \mathcal{H}_{4}\mathcal{H}_{5}}, $$

we obtain

$$\begin{aligned} \frac{d\Phi _{2}}{dt} & =\mathcal{P} \biggl[ \biggl( 1-\frac{S_{2}}{S} \biggr) ( \varrho S_{2}-\varrho S ) +\vartheta _{3}S_{2}Y_{2} \biggl( 1-\frac{S_{2}}{S} \biggr) \\ &\quad {}- \frac{\vartheta _{3}S_{2}Y_{2}}{\mathcal{H}_{4}}\int _{0}^{\kappa _{4}}\bar{\mathcal{H}}_{4}( \boldsymbol{\ell }) \frac{S(t-\boldsymbol{\ell })Y(t-\boldsymbol{\ell })E_{2}}{S_{2}Y_{2}E}\,d \boldsymbol{\ell }-\frac{rY_{2}}{\varphi \mathcal{H}_{4}} \frac{YE_{2}}{Y_{2}E} \\ &\quad {}+\vartheta _{3}S_{2}Y_{2}+\frac{rY_{2}}{\varphi \mathcal{H}_{4}}- \biggl( \frac{\vartheta _{3}S_{2}Y_{2}}{\mathcal{H}_{5}}+ \frac{rY_{2}}{\varphi \mathcal{H}_{4}\mathcal{H}_{5}} \biggr) \int _{0}^{\kappa _{5}}\bar{\mathcal{H}}_{5}( \boldsymbol{\ell }) \frac{E(t-\boldsymbol{\ell })Y_{2}}{E_{2}Y}\,d\boldsymbol{\ell } \\ & \quad{} +\vartheta _{3}S_{2}Y_{2}+ \frac{rY_{2}}{\varphi \mathcal{H}_{4}}+ \frac{\mu _{2} ( \psi +\omega ) }{\varphi \psi \mathcal{H}_{4}\mathcal{H}_{5}}C^{Y}Y_{2}- \frac{\mu _{1}\pi _{1} ( \gamma +\lambda ) }{\sigma _{1}\mathcal{P}}C^{I}- \frac{\mu _{2}\pi _{2} ( \psi +\omega ) }{\sigma _{2}\varphi \psi \mathcal{H}_{4}\mathcal{H}_{5}}C^{Y} \\ & \quad{} +\frac{\vartheta _{3}S_{2}Y_{2}}{\mathcal{H}_{4}} \int _{0}^{\kappa _{4}}\bar{\mathcal{H}}_{4}(\boldsymbol{\ell })\ln \biggl( \frac{S(t-\boldsymbol{\ell })Y(t-\boldsymbol{\ell })}{SY} \biggr) \,d \boldsymbol{\ell }\\ &\quad {}+ \biggl( \frac{\vartheta _{3}S_{2}Y_{2}}{\mathcal{H}_{5}}+ \frac{rY_{2}}{\varphi \mathcal{H}_{4}\mathcal{H}_{5}} \biggr) \int _{0}^{\kappa _{5}}\bar{\mathcal{H}}_{5}( \boldsymbol{\ell })\ln \biggl( \frac{E(t-\boldsymbol{\ell })}{E} \biggr) \,d \boldsymbol{\ell } \\ & \quad {} + \frac{a ( \lambda +\gamma ) }{\mathcal{P}} \biggl\{ \frac{\mathcal{P}S_{2} ( \varepsilon \vartheta _{2}+b\vartheta _{1}\mathcal{H}_{6} ) }{a\varepsilon ( \lambda +\gamma ) }-1 \biggr\} I \biggr] . \end{aligned}$$

Using the equalities given by (11) in case of $m=2$, we get

16 $$\begin{aligned} \frac{d\Phi _{2}}{dt} & =-\mathcal{P} \biggl[ \varrho \frac{ ( S-S_{2} ) ^{2}}{S}+ \vartheta _{3}S_{2}Y_{2} \biggl\{ \frac{S_{2}}{S}-1-\ln \biggl( \frac{S_{2}}{S} \biggr) \biggr\} + \frac{rY_{2}}{\varphi \mathcal{H}_{4}} \biggl\{ \frac{YE_{2}}{Y_{2}E}-1- \ln \biggl( \frac{YE_{2}}{Y_{2}E} \biggr) \biggr\} \\ & \quad{} +\frac{\vartheta _{3}S_{2}Y_{2}}{\mathcal{H}_{4}} \int _{0}^{\kappa _{4}}\bar{\mathcal{H}}_{4}(\boldsymbol{\ell }) \biggl\{ \frac{S(t-\boldsymbol{\ell })Y(t-\boldsymbol{\ell })E_{2}}{S_{2}Y_{2}E}-1-\ln \biggl( \frac{S(t-\boldsymbol{\ell })Y(t-\boldsymbol{\ell })E_{2}}{S_{2}Y_{2}E} \biggr) \biggr\} \,d\boldsymbol{\ell } \\ & \quad{} + \biggl( \frac{\vartheta _{3}S_{2}Y_{2}}{\mathcal{H}_{5}}+\frac{rY_{2}}{\varphi \mathcal{H}_{4}\mathcal{H}_{5}} \biggr) \int _{0}^{\kappa _{5}}\bar{\mathcal{H}}_{5}( \boldsymbol{\ell }) \biggl\{ \frac{E(t-\boldsymbol{\ell })Y_{2}}{E_{2}Y}-1-\ln \biggl( \frac{E(t-\boldsymbol{\ell })Y_{2}}{E_{2}Y} \biggr) \biggr\} \,d \boldsymbol{\ell } \\ & \quad {} - \frac{\mu _{1}\pi _{1} ( \gamma +\lambda ) }{\sigma _{1}\mathcal{P}}C^{I}+ \frac{\mu _{2} ( \psi +\omega ) }{\varphi \psi \mathcal{H}_{4}\mathcal{H}_{5}} \biggl( Y_{2}-\frac{\pi _{2}}{\sigma _{2}} \biggr) C^{Y} \biggr] \\ &\quad {}+a ( \lambda +\gamma ) \biggl( \frac{\mathcal{P}S_{2} ( \varepsilon \vartheta _{2}+b\vartheta _{1}\mathcal{H}_{6} ) }{a\varepsilon ( \lambda +\gamma ) }-1 \biggr) I. \end{aligned}$$

Therefore, Eq. (16) becomes

$$\begin{aligned} \frac{d\Phi _{2}}{dt} & =-\mathcal{P} \biggl[ \varrho \frac{ ( S-S_{2} ) ^{2}}{S}+ \frac{rY_{2}}{\varphi \mathcal{H}_{4}} \digamma \biggl( \frac{YE_{2}}{Y_{2}E} \biggr)\\ &\quad {} + \frac{\vartheta _{3}S_{2}Y_{2}}{\mathcal{H}_{4}} \int _{0}^{\kappa _{4}}\bar{\mathcal{H}}_{4}( \boldsymbol{\ell }) \biggl\{ \digamma \biggl( \frac{S(t-\boldsymbol{\ell })Y(t-\boldsymbol{\ell })E_{2}}{S_{2}Y_{2}E} \biggr)+\digamma \biggl( \frac{S_{2}}{S} \biggr) \biggr\} \,d \boldsymbol{\ell } \\ & \quad{}+ \biggl( \frac{\vartheta _{3}S_{2}Y_{2}}{\mathcal{H}_{5}}+\frac{rY_{2}}{\varphi \mathcal{H}_{4}\mathcal{H}_{5}} \biggr) \int _{0}^{\kappa _{5}}\bar{\mathcal{H}}_{5}( \boldsymbol{\ell }) \digamma \biggl( \frac{E(t-\boldsymbol{\ell })Y_{2}}{E_{2}Y} \biggr) \,d \boldsymbol{ \ell } \\ & \quad {} - \frac{\mu _{1}\pi _{1} ( \gamma +\lambda ) }{\sigma _{1}\mathcal{P}}C^{I}+ \frac{\mu _{2} ( \psi +\omega ) ( \varrho \sigma _{2}+\vartheta _{3}\pi _{2} ) }{\vartheta _{3}\sigma _{2}\varphi \psi \mathcal{H}_{4}\mathcal{H}_{5}} ( \Re _{4}-1 ) C^{Y} \biggr] \\ &\quad {}+a ( \lambda +\gamma ) \biggl( \frac{\Re _{1}}{\Re _{2}}-1 \biggr) I. \end{aligned}$$

Thus, if $\Re _{1}/\Re _{2}\leq 1$ and $\Re _{4}\leq 1$, then $\frac{d\Phi _{2}}{dt}\leq 0$ for all $S,I,E,Y,C^{I},C^{Y}>0$. Moreover, $\frac{d\Phi _{2}}{dt}=0$ when $(S,E,Y,I,C^{I},C^{Y})=(S_{2},E_{2},Y_{2},0,0,0)$. The solutions of system (5) converge to $\Upsilon _{2}^{{\prime }}$ which includes elements with $(S(t),E(t),Y(t),I(t),C^{I}(t),C^{Y}(t))=(S_{2},E_{2},Y_{2},0,0,0)$. Then we have $\frac{dS(t)}{dt}=0$, and the first equation of system (5) becomes

$$ 0=\frac{dS(t)}{dt}=\eta -\varrho S_{2}-\vartheta _{1}S_{2}V(t)- \vartheta _{3}S_{2}Y_{2}, $$

which yields $V(t)=0$ for all *t*. Moreover, we have $\frac{dI(t)}{dt}=0$ and from the third equation of system (5) we get

$$ 0=\frac{dI(t)}{dt}=\lambda \int _{0}^{\kappa _{3}} \bar{\mathcal{H}}_{3}( \boldsymbol{\ell })L(t-\boldsymbol{\ell })\,d\boldsymbol{\ell }, $$

which implies that $L(t)=0$ for all *t*. Therefore, . Applying LLAS theorem, we get  is GAS. □

### Theorem 4

*Let* $\Re _{3}>1$ *and* $\Re _{5}\leq 1$, *then*  *is GAS*.

### Proof

Define a functional as follows:

17 $$\begin{aligned} \Phi _{3} & =\mathcal{P}S_{3}\digamma \biggl( \frac{S}{S_{3}} \biggr) + \lambda \mathcal{H}_{3}L_{3} \digamma \biggl( \frac{L}{L_{3}} \biggr) + ( \gamma +\lambda ) I_{3}\digamma \biggl( \frac{I}{I_{3}} \biggr) + \frac{\mathcal{P}}{\varphi \mathcal{H}_{4}}E+ \frac{\mathcal{P} ( \psi +\omega ) }{\varphi \psi \mathcal{H}_{4}\mathcal{H}_{5}}Y \\ &\quad{}+\frac{\mathcal{P}\vartheta _{1}S_{3}}{\varepsilon }V_{3}\digamma \biggl( \frac{V}{V_{3}} \biggr) + \frac{\mu _{1} ( \gamma +\lambda ) }{\sigma _{1}}C_{3}^{I}\digamma \biggl( \frac{C^{I}}{C_{3}^{I}} \biggr) + \frac{\mu _{2}\mathcal{P} ( \psi +\omega ) }{\sigma _{2}\varphi \psi \mathcal{H}_{4}\mathcal{H}_{5}}C^{Y} \\ &\quad{}+\vartheta _{1}\lambda \mathcal{H}_{3} ( 1-\beta ) S_{3}V_{3}\int _{0}^{\kappa _{1}}\bar{\mathcal{H}}_{1}( \boldsymbol{\ell }) \int _{t-\boldsymbol{\ell }}^{t}\digamma \biggl( \frac{S(\varkappa )V(\varkappa )}{S_{3}V_{3}} \biggr) \,d\varkappa \,d \boldsymbol{\ell }+\vartheta _{2}\lambda \mathcal{H}_{3} ( 1-\beta ) S_{3}I_{3} \\ &\quad \times{} \int _{0}^{\kappa _{1}}\bar{\mathcal{H}}_{1}( \boldsymbol{\ell }) \int _{t-\boldsymbol{\ell }}^{t}\digamma \biggl( \frac{S(\varkappa )I(\varkappa )}{S_{3}I_{3}} \biggr) \,d\varkappa \,d \boldsymbol{\ell }+\vartheta _{1}\beta ( \gamma + \lambda ) S_{3}V_{3} \int _{0}^{\kappa _{2}}\bar{\mathcal{H}}_{2}( \boldsymbol{\ell }) \\ &\quad{}\times \int _{t-\boldsymbol{\ell }}^{t}\digamma \biggl( \frac{S(\varkappa )V(\varkappa )}{S_{3}V_{3}} \biggr) \,d\varkappa \,d \boldsymbol{\ell } \\ &\quad {}+\vartheta _{2}\beta ( \gamma + \lambda ) S_{3}I_{3} \int _{0}^{\kappa _{2}}\bar{\mathcal{H}}_{2}( \boldsymbol{\ell }) \int _{t-\boldsymbol{\ell }}^{t}\digamma \biggl( \frac{S(\varkappa )I(\varkappa )}{S_{3}I_{3}} \biggr) \,d\varkappa \,d\boldsymbol{\ell } \\ &\quad{}+\lambda ( \gamma +\lambda ) L_{3} \int _{0}^{ \kappa _{3}}\bar{\mathcal{H}}_{3}( \boldsymbol{\ell }) \int _{t-\boldsymbol{\ell }}^{t}\digamma \biggl( \frac{L(\varkappa )}{L_{3}} \biggr) \,d\varkappa \,d \boldsymbol{\ell } \\ &\quad {}+\frac{\mathcal{P}\vartheta _{3}}{\mathcal{H}_{4}} \int _{0}^{\kappa _{4}}\bar{\mathcal{H}}_{4}( \boldsymbol{\ell }) \int _{t-\boldsymbol{\ell }}^{t}S(\varkappa )Y(\varkappa )\,d \varkappa \,d\boldsymbol{\ell } \\ &\quad{}+ \frac{\mathcal{P} ( \psi +\omega ) }{\varphi \mathcal{H}_{4}\mathcal{H}_{5}} \int _{0}^{\kappa _{5}} \bar{\mathcal{H}}_{5}( \boldsymbol{\ell }) \int _{t-\boldsymbol{\ell }}^{t}E(\varkappa )\,d \varkappa \,d\boldsymbol{ \ell } \\ &\quad {}+ \frac{b\mathcal{P}\vartheta _{1}S_{3}I_{3}}{\varepsilon }\int _{0}^{\kappa _{6}}\bar{\mathcal{H}}_{6}( \boldsymbol{\ell }) \int _{t-\boldsymbol{\ell }}^{t}\digamma \biggl( \frac{I(\varkappa )}{I_{3}} \biggr) \,d\varkappa \,d\boldsymbol{\ell }. \end{aligned}$$

We calculate $\frac{d\Phi _{3}}{dt}$ as follows:

18 $$\begin{aligned} \frac{d\Phi _{3}}{dt} & =\mathcal{P} \biggl( 1-\frac{S_{3}}{S} \biggr) ( \eta -\varrho S-\vartheta _{1}SV-\vartheta _{2}SI-\vartheta _{3}SY ) +\lambda \mathcal{H}_{3} \biggl( 1- \frac{L_{3}}{L} \biggr) \\ &\quad{}\times \biggl[ ( 1-\beta ) \int _{0}^{\kappa _{1}}\bar{\mathcal{H}}_{1}( \boldsymbol{\ell })S(t-\boldsymbol{\ell }) \bigl\{ \vartheta _{1}V(t- \boldsymbol{\ell })+\vartheta _{2}I(t-\boldsymbol{\ell }) \bigr\} \,d \boldsymbol{\ell }- ( \lambda +\gamma ) L \biggr] \\ &\quad{}+ ( \gamma +\lambda ) \biggl( 1-\frac{I_{3}}{I} \biggr) \biggl[ \beta \int _{0}^{\kappa _{2}}\bar{\mathcal{H}}_{2}( \boldsymbol{\ell })S(t-\boldsymbol{\ell }) \bigl\{ \vartheta _{1}V(t- \boldsymbol{\ell })+\vartheta _{2}I(t-\boldsymbol{\ell }) \bigr\} \,d \boldsymbol{\ell } \\ & \quad{} +\lambda \int _{0}^{\kappa _{3}}\bar{\mathcal{H}}_{3}( \boldsymbol{\ell })L(t-\boldsymbol{\ell })\,d\boldsymbol{\ell }-aI-\mu _{1}C^{I}I \biggr] \\ &\quad {}+\frac{\mathcal{P}}{\varphi \mathcal{H}_{4}} \biggl[ \varphi \vartheta _{3}\int _{0}^{\kappa _{4}}\bar{\mathcal{H}}_{4}( \boldsymbol{\ell })S(t-\boldsymbol{\ell })Y(t-\boldsymbol{\ell })\,d \boldsymbol{\ell }+rY- ( \psi +\omega ) E \biggr] \\ & \quad{} + \frac{\mathcal{P} ( \psi +\omega ) }{\varphi \psi \mathcal{H}_{4}\mathcal{H}_{5}} \biggl[ \psi \int _{0}^{\kappa _{5}}\bar{\mathcal{H}}_{5}( \boldsymbol{\ell })E(t-\boldsymbol{\ell })\,d\boldsymbol{\ell }-\delta Y-\mu _{2}C^{Y}Y \biggr] \\ &\quad{}+\frac{\mathcal{P}\vartheta _{1}S_{3}}{\varepsilon } \biggl( 1- \frac{V_{3}}{V} \biggr) \biggl[ b \int _{0}^{\kappa _{6}} \bar{\mathcal{H}}_{6}( \boldsymbol{\ell })I(t-\boldsymbol{\ell })\,d\boldsymbol{\ell }-\varepsilon V \biggr] + \frac{\mu _{1} ( \gamma +\lambda ) }{\sigma _{1}} \\ &\quad{}\times \biggl( 1-\frac{C_{3}^{I}}{C^{I}} \biggr) \bigl( \sigma _{1}C^{I}I-\pi _{1}C^{I} \bigr) + \frac{\mu _{2}\mathcal{P} ( \psi +\omega ) }{\sigma _{2}\varphi \psi \mathcal{H}_{4}\mathcal{H}_{5}} \bigl( \sigma _{2}C^{Y}Y-\pi _{2}C^{Y} \bigr) \\ &\quad{}+\vartheta _{1}\lambda \mathcal{H}_{3} ( 1-\beta ) S_{3}V_{3} \\ &\quad {}\times \int _{0}^{\kappa _{1}}\bar{\mathcal{H}}_{1}( \boldsymbol{\ell }) \biggl[ \frac{SV}{S_{3}V_{3}}- \frac{S(t-\boldsymbol{\ell })V(t-\boldsymbol{\ell })}{S_{3}V_{3}}+\ln \biggl( \frac{S(t-\boldsymbol{\ell })V(t-\boldsymbol{\ell })}{SV} \biggr) \biggr] \,d\boldsymbol{\ell } \\ &\quad{}+\vartheta _{2}\lambda \mathcal{H}_{3} ( 1-\beta ) S_{3}I_{3} \\ &\quad {}\times \int _{0}^{\kappa _{1}}\bar{\mathcal{H}}_{1}( \boldsymbol{\ell }) \biggl[ \frac{SI}{S_{3}I_{3}}- \frac{S(t-\boldsymbol{\ell })I(t-\boldsymbol{\ell })}{S_{3}I_{3}}+\ln \biggl( \frac{S(t-\boldsymbol{\ell })I(t-\boldsymbol{\ell })}{SI} \biggr) \biggr] \,d\boldsymbol{\ell } \\ &\quad{}+\vartheta _{1}\beta ( \gamma +\lambda ) S_{3}V_{3} \\ &\quad {}\times \int _{0}^{\kappa _{2}}\bar{\mathcal{H}}_{2}( \boldsymbol{\ell }) \biggl[ \frac{SV}{S_{3}V_{3}}- \frac{S(t-\boldsymbol{\ell })V(t-\boldsymbol{\ell })}{S_{3}V_{3}}+ \ln \biggl( \frac{S(t-\boldsymbol{\ell })V(t-\boldsymbol{\ell })}{SV} \biggr) \biggr] \,d\boldsymbol{\ell } \\ &\quad{}+\vartheta _{2}\beta ( \gamma +\lambda ) S_{3}I_{3} \\ &\quad {}\times \int _{0}^{\kappa _{2}}\bar{\mathcal{H}}_{2}( \boldsymbol{\ell }) \biggl[ \frac{SI}{S_{3}I_{3}}- \frac{S(t-\boldsymbol{\ell })I(t-\boldsymbol{\ell })}{S_{3}I_{3}}+ \ln \biggl( \frac{S(t-\boldsymbol{\ell })I(t-\boldsymbol{\ell })}{SI} \biggr) \biggr] \,d\boldsymbol{\ell } \\ &\quad{}+\lambda ( \gamma +\lambda ) L_{3} \int _{0}^{ \kappa _{3}}\bar{\mathcal{H}}_{3}( \boldsymbol{\ell }) \biggl[ \frac{L}{L_{3}}- \frac{L(t-\boldsymbol{\ell })}{L_{3}}+\ln \biggl( \frac{L(t-\boldsymbol{\ell })}{L} \biggr) \biggr] \,d\boldsymbol{\ell } \\ &\quad{}+\frac{\mathcal{P}\vartheta _{3}}{\mathcal{H}_{4}} \int _{0}^{\kappa _{4}}\bar{\mathcal{H}}_{4}(\boldsymbol{\ell }) \bigl[ SY-S(t- \boldsymbol{\ell })Y(t- \boldsymbol{\ell }) \bigr] \,d\boldsymbol{\ell } \\ &\quad {}+ \frac{\mathcal{P} ( \psi +\omega ) }{\varphi \mathcal{H}_{4}\mathcal{H}_{5}} \int _{0}^{\kappa _{5}}\bar{\mathcal{H}}_{5}( \boldsymbol{\ell }) \bigl[ E-E(t- \boldsymbol{\ell }) \bigr] \,d\boldsymbol{\ell } \\ &\quad{}+\frac{b\mathcal{P}\vartheta _{1}S_{3}I_{3}}{\varepsilon } \int _{0}^{\kappa _{6}}\bar{\mathcal{H}}_{6}(\boldsymbol{\ell }) \biggl[ \frac{I}{I_{3}}- \frac{I(t-\boldsymbol{\ell })}{I_{3}}+\ln \biggl( \frac{I(t-\boldsymbol{\ell })}{I} \biggr) \biggr] \,d\boldsymbol{ \ell }. \end{aligned}$$

Collecting the terms of Eq. (18), we derive

$$\begin{aligned} \frac{d\Phi _{3}}{dt} & =\mathcal{P} \biggl( 1-\frac{S_{3}}{S} \biggr) ( \eta -\varrho S ) +\mathcal{P}\vartheta _{2}S_{3}I+ \mathcal{P}\vartheta _{3}S_{3}Y-\vartheta _{1}\lambda \mathcal{H}_{3} ( 1- \beta ) \int _{0}^{\kappa _{1}}\bar{\mathcal{H}}_{1}( \boldsymbol{\ell }) \\ &\quad{}\times \frac{S(t-\boldsymbol{\ell })V(t-\boldsymbol{\ell })L_{3}}{L}\,d \boldsymbol{\ell }-\vartheta _{2} \lambda \mathcal{H}_{3} ( 1-\beta ) \int _{0}^{\kappa _{1}}\bar{\mathcal{H}}_{1}( \boldsymbol{\ell })\frac{S(t-\boldsymbol{\ell })I(t-\boldsymbol{\ell })L_{3}}{L}\,d \boldsymbol{\ell } \\ &\quad{}+\lambda \mathcal{H}_{3} ( \lambda +\gamma ) L_{3}-a ( \lambda +\gamma ) I-\vartheta _{1}\beta ( \gamma +\lambda ) \int _{0}^{\kappa _{2}} \bar{\mathcal{H}}_{2}( \boldsymbol{\ell }) \frac{S(t-\boldsymbol{\ell })V(t-\boldsymbol{\ell })I_{3}}{I}\,d\boldsymbol{\ell } \\ &\quad{}-\vartheta _{2}\beta ( \gamma +\lambda ) \int _{0}^{\kappa _{2}}\bar{\mathcal{H}}_{2}(\boldsymbol{\ell }) \frac{S(t-\boldsymbol{\ell })I(t-\boldsymbol{\ell })I_{3}}{I}\,d\boldsymbol{\ell }\\ &\quad {}- \lambda ( \lambda +\gamma ) \int _{0}^{\kappa _{3}} \bar{\mathcal{H}}_{3}( \boldsymbol{\ell })\frac{L(t-\boldsymbol{\ell })I_{3}}{I}\,d\boldsymbol{\ell }+a ( \lambda +\gamma ) I_{3}+\mu _{1} ( \lambda + \gamma ) C^{I}I_{3} \\ &\quad{}+ \frac{\mathcal{P}r}{\varphi \mathcal{H}_{4}}Y- \frac{\mathcal{P}\delta ( \psi +\omega ) }{\varphi \psi \mathcal{H}_{4}\mathcal{H}_{5}}Y- \frac{\mathcal{P}b\vartheta _{1}S_{3}}{\varepsilon } \int _{0}^{\kappa _{6}}\bar{\mathcal{H}}_{6}( \boldsymbol{\ell })\frac{I(t-\boldsymbol{\ell })V_{3}}{V}\,d\boldsymbol{\ell } \\ &\quad{}+ \mathcal{P}\vartheta _{1}S_{3}V_{3}- \frac{\mu _{1}\pi _{1} ( \lambda +\gamma ) }{\sigma _{1}}C^{I}- \mu _{1} ( \lambda +\gamma ) C_{3}^{I}I \\ &\quad{}+ \frac{\mu _{1}\pi _{1} ( \lambda +\gamma ) }{\sigma _{1}}C_{3}^{I}- \frac{\mu _{2}\pi _{2}\mathcal{P} ( \psi +\omega ) }{\sigma _{2}\varphi \psi \mathcal{H}_{4}\mathcal{H}_{5}}C^{Y} \\ &\quad{}+ \lambda \mathcal{H}_{3} ( 1-\beta ) \vartheta _{1}S_{3}V_{3} \int _{0}^{ \kappa _{1}}\bar{\mathcal{H}}_{1}( \boldsymbol{\ell }) \ln \biggl( \frac{S(t-\boldsymbol{\ell })V(t-\boldsymbol{\ell })}{SV} \biggr) \,d\boldsymbol{\ell }\\ &\quad {}+ \lambda \mathcal{H}_{3} ( 1-\beta ) \vartheta _{2}S_{3}I_{3} \int _{0}^{\kappa _{1}} \bar{\mathcal{H}}_{1}( \boldsymbol{\ell })\ln \biggl( \frac{S(t-\boldsymbol{\ell })I(t-\boldsymbol{\ell })}{SI} \biggr) \,d \boldsymbol{\ell } \\ &\quad{}+\beta ( \gamma +\lambda ) \vartheta _{1}S_{3}V_{3} \int _{0}^{\kappa _{2}}\bar{\mathcal{H}}_{2}( \boldsymbol{\ell }) \ln \biggl( \frac{S(t-\boldsymbol{\ell })V(t-\boldsymbol{\ell })}{SV} \biggr) \,d \boldsymbol{\ell }\\ &\quad {}+\beta ( \gamma +\lambda ) \vartheta _{2}S_{3}I_{3} \int _{0}^{\kappa _{2}}\bar{\mathcal{H}}_{2}( \boldsymbol{\ell })\ln \biggl( \frac{S(t-\boldsymbol{\ell })I(t-\boldsymbol{\ell })}{SI} \biggr) \,d\boldsymbol{\ell } \\ &\quad{} + \lambda ( \gamma +\lambda ) L_{3} \int _{0}^{\kappa _{3}}\bar{\mathcal{H}}_{3}(\boldsymbol{\ell })\ln \biggl( \frac{L(t-\boldsymbol{\ell })}{L} \biggr) \,d\boldsymbol{\ell } \\ &\quad{}+ \frac{b\mathcal{P}\vartheta _{1}S_{3}\mathcal{H}_{6}}{\varepsilon }I+\frac{b\mathcal{P}\vartheta _{1}S_{3}I_{3}}{\varepsilon } \int _{0}^{\kappa _{6}}\bar{\mathcal{H}}_{6}(\boldsymbol{\ell })\ln \biggl( \frac{I(t-\boldsymbol{\ell })}{I} \biggr) \,d\boldsymbol{\ell }. \end{aligned}$$

Using the steady state conditions for

$$\begin{aligned} & \eta =\varrho S_{3}+\vartheta _{1}S_{3}V_{3}+ \vartheta _{2}S_{3}I_{3}, \qquad \mathcal{H}_{1} ( 1-\beta ) ( \vartheta _{1}S_{3}V_{3}+ \vartheta _{2}S_{3}I_{3} ) = ( \lambda +\gamma ) L_{3}, \\ & \beta \mathcal{H}_{2} ( \vartheta _{1}S_{3}V_{3}+ \vartheta _{2}S_{3}I_{3} ) +\lambda \mathcal{H}_{3}L_{3}= \bigl( a+\mu _{1}C_{3}^{I} \bigr) I_{3}, \qquad I_{3}= \frac{\pi _{1}}{\sigma _{1}}, \qquad V_{3}= \frac{b\mathcal{H}_{6}}{\varepsilon }I_{3}, \end{aligned}$$

we get

$$ \mathcal{P} ( \vartheta _{1}S_{3}V_{3}+ \vartheta _{2}S_{3}I_{3} ) = ( \lambda +\gamma ) \bigl( a+\mu _{1}C_{3}^{I} \bigr) I_{3}. $$

Further, we obtain

$$\begin{aligned} \frac{d\Phi _{3}}{dt} & =\mathcal{P} \biggl( 1-\frac{S_{3}}{S} \biggr) ( \varrho S_{3}-\varrho S ) +\mathcal{P} ( \vartheta _{1}S_{3}V_{3}+ \vartheta _{2}S_{3}I_{3} ) \biggl( 1- \frac{S_{3}}{S} \biggr) +\mathcal{P}\vartheta _{3}S_{3}Y \\ &\quad{}-\lambda \mathcal{H}_{3} ( 1-\beta ) \vartheta _{1}S_{3}V_{3}\int _{0}^{\kappa _{1}}\bar{\mathcal{H}}_{1}( \boldsymbol{\ell }) \frac{S(t-\boldsymbol{\ell })V(t-\boldsymbol{\ell })L_{3}}{S_{3}V_{3}L}\,d \boldsymbol{\ell }-\lambda \mathcal{H}_{3} ( 1-\beta ) \vartheta _{2}S_{3}I_{3} \\ &\quad{}\times \int _{0}^{\kappa _{1}}\bar{\mathcal{H}}_{1}( \boldsymbol{\ell })\frac{S(t-\boldsymbol{\ell })I(t-\boldsymbol{\ell })L_{3}}{S_{3}I_{3}L}\,d \boldsymbol{\ell }+\lambda \mathcal{H}_{1}\mathcal{H}_{3} ( 1-\beta ) ( \vartheta _{1}S_{3}V_{3}+\vartheta _{2}S_{3}I_{3} ) \\ &\quad{}-\beta ( \gamma +\lambda ) \vartheta _{1}S_{3}V_{3} \int _{0}^{\kappa _{2}}\bar{\mathcal{H}}_{2}( \boldsymbol{\ell }) \frac{S(t-\boldsymbol{\ell })V(t-\boldsymbol{\ell })I_{3}}{S_{3}V_{3}I}\,d \boldsymbol{\ell }-\beta ( \gamma +\lambda ) \vartheta _{2}S_{3}I_{3} \int _{0}^{\kappa _{2}}\bar{\mathcal{H}}_{2}( \boldsymbol{\ell }) \\ &\quad{}\times \frac{S(t-\boldsymbol{\ell })I(t-\boldsymbol{\ell })}{S_{3}I}\,d \boldsymbol{\ell }-\lambda \mathcal{H}_{1} ( 1-\beta ) ( \vartheta _{1}S_{3}V_{3}+ \vartheta _{2}S_{3}I_{3} ) \int _{0}^{ \kappa _{3}}\bar{\mathcal{H}}_{3}( \boldsymbol{\ell }) \frac{L(t-\boldsymbol{\ell })I_{3}}{L_{3}I}\,d\boldsymbol{\ell } \\ &\quad{}+\mathcal{P} ( \vartheta _{1}S_{3}V_{3}+ \vartheta _{2}S_{3}I_{3} ) - \frac{\mathcal{P} [ ( \delta -r\mathcal{H}_{5} ) \psi +\delta \omega ] }{\varphi \psi \mathcal{H}_{4}\mathcal{H}_{5}}Y-\frac{\mathcal{P}\vartheta _{1}S_{3}V_{3}}{\mathcal{H}_{6}} \int _{0}^{\kappa _{6}}\bar{\mathcal{H}}_{6}( \boldsymbol{\ell }) \\ &\quad{}\times \frac{I(t-\boldsymbol{\ell })V_{3}}{I_{3}V}\,d\boldsymbol{\ell }+ \mathcal{P}\vartheta _{1}S_{3}V_{3}- \frac{\mu _{2}\pi _{2}\mathcal{P} ( \psi +\omega ) }{\sigma _{2}\varphi \psi \mathcal{H}_{4}\mathcal{H}_{5}}C^{Y}+ \lambda \mathcal{H}_{3} ( 1-\beta ) \vartheta _{1}S_{3}V_{3} \\ &\quad{}\times \int _{0}^{\kappa _{1}}\bar{\mathcal{H}}_{1}( \boldsymbol{\ell })\ln \biggl( \frac{S(t-\boldsymbol{\ell })V(t-\boldsymbol{\ell })}{SV} \biggr) \,d \boldsymbol{\ell } \\ &\quad{}+\lambda \mathcal{H}_{3} ( 1-\beta ) \vartheta _{2}S_{3}I_{3} \int _{0}^{\kappa _{1}} \bar{\mathcal{H}}_{1}( \boldsymbol{\ell }) \ln \biggl( \frac{S(t-\boldsymbol{\ell })I(t-\boldsymbol{\ell })}{SI} \biggr) \,d\boldsymbol{\ell }\\ &\quad {}+\beta ( \gamma +\lambda ) \vartheta _{1}S_{3}V_{3}\int _{0}^{\kappa _{2}}\bar{\mathcal{H}}_{2}( \boldsymbol{\ell }) \ln \biggl( \frac{S(t-\boldsymbol{\ell })V(t-\boldsymbol{\ell })}{SV} \biggr) \,d \boldsymbol{\ell } \\ &\quad{}+\beta ( \gamma +\lambda ) \vartheta _{2}S_{3}I_{3} \int _{0}^{\kappa _{2}}\bar{\mathcal{H}}_{2}( \boldsymbol{\ell }) \ln \biggl( \frac{S(t-\boldsymbol{\ell })I(t-\boldsymbol{\ell })}{SI} \biggr) \,d \boldsymbol{\ell }\\ &\quad {}+\lambda \mathcal{H}_{1} ( 1-\beta ) ( \vartheta _{1}S_{3}V_{3}+\vartheta _{2}S_{3}I_{3} ) \int _{0}^{\kappa _{3}}\bar{\mathcal{H}}_{3}( \boldsymbol{\ell })\ln \biggl( \frac{L(t-\boldsymbol{\ell })}{L} \biggr) \,d \boldsymbol{\ell } \\ &\quad{}+ \frac{\mathcal{P}\vartheta _{1}S_{3}V_{3}}{\mathcal{H}_{6}} \int _{0}^{\kappa _{6}}\bar{\mathcal{H}}_{6}(\boldsymbol{\ell })\ln \biggl( \frac{I(t-\boldsymbol{\ell })}{I} \biggr) \,d\boldsymbol{\ell }. \end{aligned}$$

Using the equalities given by (10) in case of $n=3$, we get

19 $$\begin{aligned} \frac{d\Phi _{3}}{dt} & =-\varrho \mathcal{P} \frac{(S-S_{3})^{2}}{S}-\mathcal{P} ( \vartheta _{1}S_{3}V_{3}+\vartheta _{2}S_{3}I_{3} ) \biggl[ \frac{S_{3}}{S}-1- \ln \biggl( \frac{S_{3}}{S} \biggr) \biggr] \\ &\quad{}-\lambda \mathcal{H}_{3} ( 1-\beta ) \vartheta _{1}S_{3}V_{3} \\ &\quad {}\times\int _{0}^{\kappa _{1}}\bar{\mathcal{H}}_{1}( \boldsymbol{\ell }) \biggl[ \frac{S(t-\boldsymbol{\ell })V(t-\boldsymbol{\ell })L_{3}}{S_{3}V_{3}L}-1- \ln \biggl( \frac{S(t-\boldsymbol{\ell })V(t-\boldsymbol{\ell })L_{3}}{S_{3}V_{3}L} \biggr) \biggr] \,d\boldsymbol{\ell } \\ &\quad{}-\lambda \mathcal{H}_{3} ( 1-\beta ) \vartheta _{2}S_{3}I_{3} \\ &\quad {}\times\int _{0}^{\kappa _{1}}\bar{\mathcal{H}}_{1}( \boldsymbol{\ell }) \biggl[ \frac{S(t-\boldsymbol{\ell })I(t-\boldsymbol{\ell })L_{3}}{S_{3}I_{3}L}-1- \ln \biggl( \frac{S(t-\boldsymbol{\ell })I(t-\boldsymbol{\ell })L_{3}}{S_{3}I_{3}L} \biggr) \biggr] \,d\boldsymbol{\ell } \\ &\quad{}-\beta ( \gamma +\lambda ) \vartheta _{1}S_{3}V_{3} \\ &\quad {}\times\int _{0}^{\kappa _{2}}\bar{\mathcal{H}}_{2}( \boldsymbol{\ell }) \biggl[ \frac{S(t-\boldsymbol{\ell })V(t-\boldsymbol{\ell })I_{3}}{S_{3}V_{3}I}-1- \ln \biggl( \frac{S(t-\boldsymbol{\ell })V(t-\boldsymbol{\ell })I_{3}}{S_{3}V_{3}I} \biggr) \biggr] \,d\boldsymbol{\ell } \\ &\quad{}-\beta ( \gamma +\lambda ) \vartheta _{2}S_{3}I_{3} \int _{0}^{\kappa _{2}}\bar{\mathcal{H}}_{2}( \boldsymbol{\ell }) \biggl[ \frac{S(t-\boldsymbol{\ell })I(t-\boldsymbol{\ell })}{S_{3}I}-1-\ln \biggl( \frac{S(t-\boldsymbol{\ell })I(t-\boldsymbol{\ell })}{S_{3}I} \biggr) \biggr] \,d\boldsymbol{\ell } \\ &\quad{}-\lambda \mathcal{H}_{1} ( 1-\beta ) ( \vartheta _{1}S_{3}V_{3}+\vartheta _{2}S_{3}I_{3} ) \\ &\quad {}\times\int _{0}^{ \kappa _{3}}\bar{\mathcal{H}}_{3}( \boldsymbol{\ell }) \biggl[ \frac{L(t-\boldsymbol{\ell })I_{3}}{L_{3}I}-1-\ln \biggl( \frac{L(t-\boldsymbol{\ell })I_{3}}{L_{3}I} \biggr) \biggr] \,d\boldsymbol{\ell } \\ &\quad{}-\frac{\mathcal{P}\vartheta _{1}S_{3}V_{3}}{\mathcal{H}_{6}} \int _{0}^{\kappa _{6}}\bar{\mathcal{H}}_{6}( \boldsymbol{\ell }) \biggl[ \frac{I(t-\boldsymbol{\ell })V_{3}}{I_{3}V}-1-\ln \biggl( \frac{I(t-\boldsymbol{\ell })V_{3}}{I_{3}V} \biggr) \biggr] \,d \boldsymbol{\ell } \\ &\quad{}+\mathcal{P}\vartheta _{3} \biggl( S_{3}- \frac{ ( \delta -r\mathcal{H}_{5} ) \psi +\delta \omega }{\vartheta _{3}\varphi \psi \mathcal{H}_{4}\mathcal{H}_{5}} \biggr) Y- \frac{\mu _{2}\pi _{2}\mathcal{P} ( \psi +\omega ) }{\sigma _{2}\varphi \psi \mathcal{H}_{4}\mathcal{H}_{5}}C^{Y}. \end{aligned}$$

Therefore, Eq. (19) becomes

$$\begin{aligned} \frac{d\Phi _{3}}{dt} & =-\varrho \mathcal{P} \frac{(S-S_{3})^{2}}{S}-\mathcal{P} ( \vartheta _{1}S_{3}V_{3}+\vartheta _{2}S_{3}I_{3} ) \digamma \biggl( \frac{S_{3}}{S} \biggr) \\ &\quad{}-\lambda \mathcal{H}_{3} ( 1-\beta ) \vartheta _{1}S_{3}V_{3}\int _{0}^{\kappa _{1}}\bar{\mathcal{H}}_{1}( \boldsymbol{\ell }) \digamma \biggl( \frac{S(t-\boldsymbol{\ell })V(t-\boldsymbol{\ell })L_{3}}{S_{3}V_{3}L} \biggr) \,d \boldsymbol{ \ell } \\ &\quad{}-\lambda \mathcal{H}_{3} ( 1-\beta ) \vartheta _{2}S_{3}I_{3}\int _{0}^{\kappa _{1}}\bar{\mathcal{H}}_{1}( \boldsymbol{\ell }) \digamma \biggl( \frac{S(t-\boldsymbol{\ell })I(t-\boldsymbol{\ell })L_{3}}{S_{3}I_{3}L} \biggr) \,d \boldsymbol{ \ell } \\ &\quad{}-\beta ( \gamma +\lambda ) \vartheta _{1}S_{3}V_{3} \int _{0}^{\kappa _{2}}\bar{\mathcal{H}}_{2}( \boldsymbol{\ell }) \digamma \biggl( \frac{S(t-\boldsymbol{\ell })V(t-\boldsymbol{\ell })I_{3}}{S_{3}V_{3}I} \biggr) \,d \boldsymbol{ \ell } \\ &\quad{}-\beta ( \gamma +\lambda ) \vartheta _{2}S_{3}I_{3} \int _{0}^{\kappa _{2}}\bar{\mathcal{H}}_{2}( \boldsymbol{\ell }) \digamma \biggl( \frac{S(t-\boldsymbol{\ell })I(t-\boldsymbol{\ell })}{S_{3}I} \biggr) \,d\boldsymbol{\ell } \\ &\quad{}-\lambda \mathcal{H}_{1} ( 1-\beta ) ( \vartheta _{1}S_{3}V_{3}+\vartheta _{2}S_{3}I_{3} ) \int _{0}^{ \kappa _{3}}\bar{\mathcal{H}}_{3}( \boldsymbol{\ell })\digamma \biggl( \frac{L(t-\boldsymbol{\ell })I_{3}}{L_{3}I} \biggr) \,d\boldsymbol{\ell } \\ &\quad{}-\frac{\mathcal{P}\vartheta _{1}S_{3}V_{3}}{\mathcal{H}_{6}} \int _{0}^{\kappa _{6}}\bar{\mathcal{H}}_{6}( \boldsymbol{\ell }) \digamma \biggl( \frac{I(t-\boldsymbol{\ell })V_{3}}{I_{3}V} \biggr) \,d \boldsymbol{ \ell }\\ &\quad {}+\mathcal{P}\vartheta _{3} ( S_{3}-S_{5} ) Y- \frac{\mu _{2}\pi _{2}\mathcal{P} ( \psi +\omega ) }{\sigma _{2}\varphi \psi \mathcal{H}_{4}\mathcal{H}_{5}}C^{Y}. \end{aligned}$$

Hence, if $\Re _{5}\leq 1$, then  does not exist since $E_{5}\leq 0$ and $Y_{5}\leq 0$. In this case

$$\begin{aligned} &\frac{dE(t)}{dt} =\varphi \vartheta _{3} \int _{0}^{\kappa _{4}}\bar{\mathcal{H}}_{4}( \boldsymbol{\ell })S(t-\boldsymbol{\ell })Y(t- \boldsymbol{\ell })\,d\boldsymbol{ \ell }+rY(t)- ( \psi +\omega ) E(t) \leq 0, \\ &\frac{dY(t)}{dt} =\psi \int _{0}^{\kappa _{5}} \bar{\mathcal{H}}_{5}( \boldsymbol{\ell })E(t-\boldsymbol{\ell })\,d\boldsymbol{\ell }-\delta Y(t)-\mu _{2}C^{Y}Y\leq 0. \end{aligned}$$

Now we want to find the value *S̄* such that, for all $0< S(t)\leq \bar{S}$, we get $\frac{dE(t)}{dt}\leq 0$ and $\frac{dY(t)}{dt}\leq 0$. Let us consider

$$\begin{aligned} & \frac{d}{dt} \biggl[ \frac{1}{\mathcal{H}_{4}}E+ \frac{\psi +\omega }{\psi \mathcal{H}_{4}\mathcal{H}_{5}}Y+ \frac{\varphi \vartheta _{3}}{\mathcal{H}_{4}} \int _{0}^{\kappa _{4}}\bar{\mathcal{H}}_{4}( \boldsymbol{\ell }) \int _{t-\boldsymbol{\ell }}^{t}S(\varkappa )Y( \varkappa )\,d\varkappa \,d\boldsymbol{\ell }\\ &\qquad {}+ \frac{\psi +\omega }{\mathcal{H}_{4}\mathcal{H}_{5}} \int _{0}^{\kappa _{5}}\bar{\mathcal{H}}_{5}( \boldsymbol{\ell }) \int _{t-\boldsymbol{\ell }}^{t}E(\varkappa )\,d\varkappa \,d \boldsymbol{ \ell } \biggr] \\ &\quad =\varphi \vartheta _{3}SY- \frac{ ( \delta -r\mathcal{H}_{5} ) \psi +\delta \omega }{\psi \mathcal{H}_{4}\mathcal{H}_{5}}Y- \frac{\mu _{2} ( \psi +\omega ) }{\psi \mathcal{H}_{4}\mathcal{H}_{5}}C^{Y}Y \\ &\quad =\varphi \vartheta _{3} \biggl( S- \frac{ ( \delta -r\mathcal{H}_{5} ) \psi +\delta \omega }{\varphi \vartheta _{3}\psi \mathcal{H}_{4}\mathcal{H}_{5}} \biggr) Y- \frac{\mu _{2} ( \psi +\omega ) }{\psi \mathcal{H}_{4}\mathcal{H}_{5}}C^{Y}Y\leq 0\quad \text{for all }C^{Y},Y>0. \end{aligned}$$

This happens when $S_{3}\leq \bar{S}= \frac{ ( \delta -r\mathcal{H}_{5} ) \psi +\delta \omega }{\vartheta _{3}\varphi \psi \mathcal{H}_{4}\mathcal{H}_{5}}=S_{5}$. Clearly, $\frac{d\Phi _{3}}{dt}\leq 0$ for all $S,L,I,Y,V,C^{Y}>0$, where $\frac{d\Phi _{3}}{dt}=0$ occurs at $S=S_{3}$ and $Y=C^{Y}=0$. The solutions of system (5) converge to $\Upsilon _{3}^{{\prime }}$ which includes elements satisfying $S(t)=S_{3}$ and $\digamma =0$ i.e.

20 $$\begin{aligned} \frac{S(t-\boldsymbol{\ell })V(t-\boldsymbol{\ell })L_{3}}{S_{3}V_{3}L} & = \frac{S(t-\boldsymbol{\ell })I(t-\boldsymbol{\ell })L_{3}}{S_{3}I_{3}L}= \frac{S(t-\boldsymbol{\ell })V(t-\boldsymbol{\ell })I_{3}}{S_{3}V_{3}I} \\ & =\frac{S(t-\boldsymbol{\ell })I(t-\boldsymbol{\ell })}{S_{3}I}= \frac{L(t-\boldsymbol{\ell })I_{3}}{L_{3}I}= \frac{I(t-\boldsymbol{\ell })V_{3}}{I_{3}V}=1 \end{aligned}$$

for all $t\in {}[ 0,\kappa ]$. If $S(t)=S_{3}$, then from Eq. (20) we get $L(t)=L_{3}$, $I(t)=I_{3}$, and $V(t)=V_{3}$ for all *t*. Thus, $\Upsilon _{3}^{{\prime }}$ contains elements with $I(t)=I_{3}$, $V(t)=V_{3}$, $Y(t)=0$, and then $\frac{dI(t)}{dt}=0$, $\frac{dY(t)}{dt}=0$. The third and fifth equations of system (5) become

$$\begin{aligned} &0 =\frac{dI(t)}{dt}=\beta \mathcal{H}_{2} ( \vartheta _{1}S_{3}V_{3}+\vartheta _{2}S_{3}I_{3} ) +\lambda \mathcal{H}_{3}L_{3}-aI_{3}- \mu _{1}C^{I}(t)I_{3}, \\ &0 =\frac{dY(t)}{dt}=\psi \int _{0}^{\kappa _{5}} \bar{\mathcal{H}}_{5}E(t- \boldsymbol{\ell })\,d\boldsymbol{\ell }, \end{aligned}$$

which yield $C^{I}(t)=C_{3}^{I}$ and $E(t)=0$ for all *t*. Therefore, . Applying LLAS theorem, we get  is GAS. □

### Theorem 5

*If* $\Re _{4}>1$ *and* $\Re _{6}\leq 1$, *then*  *is GAS*.

### Proof

Let

$$\begin{aligned} \Phi _{4} & =\mathcal{P}S_{4}\digamma \biggl( \frac{S}{S_{4}} \biggr) + \lambda \mathcal{H}_{3}L+ ( \gamma + \lambda ) I+ \frac{\mathcal{P}}{\varphi \mathcal{H}_{4}}E_{4}\digamma \biggl( \frac{E}{E_{4}} \biggr) + \frac{\mathcal{P} ( \psi +\omega ) }{\varphi \psi \mathcal{H}_{4}\mathcal{H}_{5}}Y_{4}\digamma \biggl( \frac{Y}{Y_{4}} \biggr) \\ &\quad{}+\frac{\mathcal{P}\vartheta _{1}S_{4}}{\varepsilon }V+ \frac{\mu _{1} ( \gamma +\lambda ) }{\sigma _{1}}C^{I}+ \frac{\mu _{2}\mathcal{P} ( \psi +\omega ) }{\sigma _{2}\varphi \psi \mathcal{H}_{4}\mathcal{H}_{5}}C_{4}^{Y}\digamma \biggl( \frac{C^{Y}}{C_{4}^{Y}} \biggr) \\ &\quad{}+\lambda \mathcal{H}_{3} ( 1-\beta ) \int _{0}^{\kappa _{1}}\bar{\mathcal{H}}_{1}( \boldsymbol{\ell }) \int _{t-\boldsymbol{\ell }}^{t}S(\varkappa ) \bigl[ \vartheta _{1}V(\varkappa )+\vartheta _{2}I(\varkappa ) \bigr] \,d \varkappa \,d\boldsymbol{\ell }\\ &\quad {}+\beta ( \gamma +\lambda ) \int _{0}^{\kappa _{2}}\bar{\mathcal{H}}_{2}( \boldsymbol{\ell }) \int _{t-\boldsymbol{\ell }}^{t}S(\varkappa ) \bigl[ \vartheta _{1}V(\varkappa )+\vartheta _{2}I( \varkappa ) \bigr] \,d\varkappa \,d\boldsymbol{\ell } \\ &\quad{}+\lambda ( \gamma +\lambda ) \int _{0}^{ \kappa _{3}}\bar{\mathcal{H}}_{3}( \boldsymbol{\ell }) \int _{t-\boldsymbol{\ell }}^{t}L(\varkappa )\,d\varkappa \,d \boldsymbol{\ell }\\ &\quad {}+ \frac{\mathcal{P}\vartheta _{3}S_{4}Y_{4}}{\mathcal{H}_{4}} \int _{0}^{\kappa _{4}} \bar{\mathcal{H}}_{4}( \boldsymbol{\ell }) \int _{t-\boldsymbol{\ell }}^{t}\digamma \biggl( \frac{S(\varkappa )Y(\varkappa )}{S_{4}Y_{4}} \biggr) \,d\varkappa \,d \boldsymbol{\ell } \\ &\quad{}+ \frac{\mathcal{P} ( \psi +\omega ) E_{4}}{\varphi \mathcal{H}_{4}\mathcal{H}_{5}} \int _{0}^{\kappa _{5}} \bar{\mathcal{H}}_{5}( \boldsymbol{\ell }) \int _{t-\boldsymbol{\ell }}^{t}\digamma \biggl( \frac{E(\varkappa )}{E_{4}} \biggr) \,d\varkappa \,d\boldsymbol{\ell }\\ &\quad {}+ \frac{b\mathcal{P}\vartheta _{1}S_{4}}{\varepsilon } \int _{0}^{ \kappa _{6}}\bar{\mathcal{H}}_{6}( \boldsymbol{\ell }) \int _{t-\boldsymbol{\ell }}^{t}I(\varkappa )\,d\varkappa \,d \boldsymbol{\ell }. \end{aligned}$$

Calculate $\frac{d\Phi _{4}}{dt}$ as follows:

21 $$\begin{aligned} \frac{d\Phi _{4}}{dt} & =\mathcal{P} \biggl( 1-\frac{S_{4}}{S} \biggr) ( \eta -\varrho S-\vartheta _{1}SV-\vartheta _{2}SI-\vartheta _{3}SY ) \\ &\quad{}+\lambda \mathcal{H}_{3} \biggl[ ( 1-\beta ) \int _{0}^{\kappa _{1}}\bar{\mathcal{H}}_{1}(\boldsymbol{\ell })S(t-\boldsymbol{\ell }) \bigl\{ \vartheta _{1}V(t-\boldsymbol{\ell })+\vartheta _{2}I(t- \boldsymbol{\ell }) \bigr\} \,d\boldsymbol{\ell }- ( \lambda +\gamma ) L \biggr] \\ &\quad{}+ ( \gamma +\lambda ) \biggl[ \beta \int _{0}^{ \kappa _{2}}\bar{\mathcal{H}}_{2}( \boldsymbol{\ell })S(t-\boldsymbol{\ell }) \bigl\{ \vartheta _{1}V(t- \boldsymbol{\ell })+\vartheta _{2}I(t-\boldsymbol{\ell }) \bigr\} \,d \boldsymbol{\ell } \\ & \quad{}+\lambda \int _{0}^{\kappa _{3}} \bar{\mathcal{H}}_{3}( \boldsymbol{\ell })L(t-\boldsymbol{\ell })\,d\boldsymbol{\ell } -aI-\mu _{1}C^{I}I \biggr] \\ &\quad {} + \frac{\mathcal{P}}{\varphi \mathcal{H}_{4}} \biggl( 1-\frac{E_{4}}{E} \biggr) \biggl[ \varphi \vartheta _{3} \int _{0}^{\kappa _{4}} \bar{\mathcal{H}}_{4}( \boldsymbol{\ell })S(t-\boldsymbol{\ell })Y(t- \boldsymbol{\ell })\,d\boldsymbol{ \ell }+rY- ( \psi +\omega ) E \biggr] \\ &\quad{}+ \frac{\mathcal{P} ( \psi +\omega ) }{\varphi \psi \mathcal{H}_{4}\mathcal{H}_{5}} \biggl( 1-\frac{Y_{4}}{Y} \biggr) \biggl[ \psi \int _{0}^{\kappa _{5}}\bar{\mathcal{H}}_{5}( \boldsymbol{\ell })E(t-\boldsymbol{\ell })\,d\boldsymbol{\ell }-\delta Y- \mu _{2}C^{Y}Y \biggr] \\ &\quad{}+\frac{\mathcal{P}\vartheta _{1}S_{4}}{\varepsilon } \biggl[ b \int _{0}^{\kappa _{6}}\bar{\mathcal{H}}_{6}( \boldsymbol{\ell })I(t- \boldsymbol{\ell })\,d\boldsymbol{\ell }-\varepsilon V \biggr] + \frac{\mu _{1} ( \gamma +\lambda ) }{\sigma _{1}} \bigl( \sigma _{1}C^{I}I-\pi _{1}C^{I} \bigr) \\ &\quad{}+ \frac{\mu _{2}\mathcal{P} ( \psi +\omega ) }{\sigma _{2}\varphi \psi \mathcal{H}_{4}\mathcal{H}_{5}} \biggl( 1-\frac{C_{4}^{Y}}{C^{Y}} \biggr) \bigl( \sigma _{2}C^{Y}Y-\pi _{2}C^{Y} \bigr) +\mathcal{P} ( \vartheta _{1}SV+ \vartheta _{2}SI ) \\ &\quad{}- \lambda \mathcal{H}_{3} ( 1-\beta ) \int _{0}^{\kappa _{1}}\bar{\mathcal{H}}_{1}( \boldsymbol{\ell })S(t-\boldsymbol{\ell }) \bigl[ \vartheta _{1}V(t-\boldsymbol{\ell })+\vartheta _{2}I(t- \boldsymbol{\ell }) \bigr] \,d\boldsymbol{\ell } \\ &\quad {}-\beta ( \gamma +\lambda ) \int _{0}^{\kappa _{2}}\bar{\mathcal{H}}_{2}( \boldsymbol{\ell })S(t-\boldsymbol{\ell }) \bigl[ \vartheta _{1}V(t- \boldsymbol{\ell })+\vartheta _{2}I(t-\boldsymbol{\ell }) \bigr] \,d \boldsymbol{\ell } \\ &\quad{}+\lambda ( \gamma +\lambda ) \int _{0}^{ \kappa _{3}}\bar{\mathcal{H}}_{3}( \boldsymbol{\ell }) \bigl[ L-L(t-\boldsymbol{\ell }) \bigr] \,d\boldsymbol{\ell } \\ &\quad {}+ \frac{\mathcal{P}\vartheta _{3}S_{4}Y_{4}}{\mathcal{H}_{4}}\int _{0}^{\kappa _{4}}\bar{\mathcal{H}}_{4}( \boldsymbol{\ell }) \biggl[ \frac{SY}{S_{4}Y_{4}}- \frac{S(t-\boldsymbol{\ell })Y(t-\boldsymbol{\ell })}{S_{4}Y_{4}} \\ & \quad{} +\ln \biggl( \frac{S(t-\boldsymbol{\ell })Y(t-\boldsymbol{\ell })}{SY} \biggr) \biggr] \,d \boldsymbol{ \ell }+ \frac{\mathcal{P} ( \psi +\omega ) E_{4}}{\varphi \mathcal{H}_{4}\mathcal{H}_{5}} \int _{0}^{\kappa _{5}}\bar{\mathcal{H}}_{5}( \boldsymbol{\ell }) \biggl[ \frac{E}{E_{4}}- \frac{E(t-\boldsymbol{\ell })}{E_{4}} \\ & \quad{} +\ln \biggl( \frac{E(t-\boldsymbol{\ell })}{E} \biggr) \biggr] \,d \boldsymbol{ \ell }+\frac{b\mathcal{P}\vartheta _{1}S_{4}}{\varepsilon }\int _{0}^{\kappa _{6}}\bar{\mathcal{H}}_{6}( \boldsymbol{\ell }) \bigl[ I-I(t-\boldsymbol{\ell }) \bigr] \,d\boldsymbol{\ell }. \end{aligned}$$

Summing the terms of Eq. (21), we get

$$\begin{aligned} \frac{d\Phi _{4}}{dt} & =\mathcal{P} \biggl[ \biggl( 1-\frac{S_{4}}{S} \biggr) ( \eta -\varrho S ) +\vartheta _{2}S_{4}I+ \vartheta _{3}S_{4}Y-\frac{a ( \lambda +\gamma ) }{\mathcal{P}}I+ \frac{r}{\varphi \mathcal{H}_{4}}Y \\ & \quad{} -\frac{\vartheta _{3}}{\mathcal{H}_{4}} \int _{0}^{ \kappa _{4}}\bar{\mathcal{H}}_{4}( \boldsymbol{\ell }) \frac{S(t-\boldsymbol{\ell })Y(t-\boldsymbol{\ell })E_{4}}{E}\,d\boldsymbol{\ell }- \frac{r}{\varphi \mathcal{H}_{4}} \frac{YE_{4}}{E}+\frac{\psi +\omega }{\varphi \mathcal{H}_{4}}E_{4} \\ & \quad{} - \frac{\delta ( \psi +\omega ) }{\varphi \psi \mathcal{H}_{4}\mathcal{H}_{5}}Y- \frac{\psi +\omega }{\varphi \mathcal{H}_{4}\mathcal{H}_{5}} \int _{0}^{\kappa _{5}} \bar{\mathcal{H}}_{5}( \boldsymbol{\ell })\frac{E(t-\boldsymbol{\ell })Y_{4}}{Y}\,d\boldsymbol{\ell }+ \frac{\delta ( \psi +\omega ) }{\varphi \psi \mathcal{H}_{4}\mathcal{H}_{5}}Y_{4} \\ & \quad{} + \frac{\mu _{2} ( \psi +\omega ) }{\varphi \psi \mathcal{H}_{4}\mathcal{H}_{5}}C^{Y}Y_{4}- \frac{\mu _{1}\pi _{1} ( \gamma +\lambda ) }{\sigma _{1}\mathcal{P}}C^{I}- \frac{\mu _{2}\pi _{2} ( \psi +\omega ) }{\sigma _{2}\varphi \psi \mathcal{H}_{4}\mathcal{H}_{5}}C^{Y}- \frac{\mu _{2} ( \psi +\omega ) }{\varphi \psi \mathcal{H}_{4}\mathcal{H}_{5}}C_{4}^{Y}Y \\ & \quad{} + \frac{\mu _{2}\pi _{2} ( \psi +\omega ) }{\sigma _{2}\varphi \psi \mathcal{H}_{4}\mathcal{H}_{5}}C_{4}^{Y}+ \frac{\vartheta _{3}S_{4}Y_{4}}{\mathcal{H}_{4}} \int _{0}^{ \kappa _{4}}\bar{\mathcal{H}}_{4}( \boldsymbol{\ell })\ln \biggl( \frac{S(t-\boldsymbol{\ell })Y(t-\boldsymbol{\ell })}{SY} \biggr) \,d\boldsymbol{\ell } \\ & \quad{} + \frac{ ( \psi +\omega ) E_{4}}{\varphi \mathcal{H}_{4}\mathcal{H}_{5}} \int _{0}^{\kappa _{5}} \bar{\mathcal{H}}_{5}( \boldsymbol{\ell })\ln \biggl( \frac{E(t-\boldsymbol{\ell })}{E} \biggr) \,d \boldsymbol{\ell }+ \frac{b\vartheta _{1}S_{4}\mathcal{H}_{6}}{\varepsilon }I \biggr] . \end{aligned}$$

Using the steady state conditions for

$$\begin{aligned} &\eta =\varrho S_{4}+\vartheta _{3}S_{4}Y_{4}, \qquad Y_{4}= \frac{\pi _{2}}{\sigma _{2}}, \\ &\vartheta _{3}S_{4}Y_{4}+\frac{rY_{4}}{\varphi \mathcal{H}_{4}} = \frac{ ( \psi +\omega ) E_{4}}{\varphi \mathcal{H}_{4}}= \frac{\delta ( \psi +\omega ) }{\varphi \psi \mathcal{H}_{4}\mathcal{H}_{5}}Y_{4}+ \frac{\mu _{2} ( \psi +\omega ) }{\varphi \psi \mathcal{H}_{4}\mathcal{H}_{5}}C_{4}^{Y}Y_{4}, \end{aligned}$$

we obtain

$$\begin{aligned} \frac{d\Phi _{4}}{dt} & =\mathcal{P} \biggl[ \biggl( 1-\frac{S_{4}}{S} \biggr) ( \varrho S_{4}-\varrho S ) +\vartheta _{3}S_{4}Y_{4} \biggl( 1-\frac{S_{4}}{S} \biggr)\\ &\quad {} - \frac{\vartheta _{3}S_{4}Y_{4}}{\mathcal{H}_{4}}\int _{0}^{\kappa _{4}}\bar{\mathcal{H}}_{4}( \boldsymbol{\ell }) \frac{S(t-\boldsymbol{\ell })Y(t-\boldsymbol{\ell })E_{4}}{S_{4}Y_{4}E}\,d\boldsymbol{\ell }\\ &\quad {}-\frac{rY_{4}}{\varphi \mathcal{H}_{4}} \frac{YE_{4}}{Y_{4}E}+\vartheta _{3}S_{4}Y_{4}+ \frac{rY_{4}}{\varphi \mathcal{H}_{4}}\\ &\quad {}- \biggl( \frac{\vartheta _{3}S_{4}Y_{4}}{\mathcal{H}_{5}}+ \frac{rY_{4}}{\varphi \mathcal{H}_{4}\mathcal{H}_{5}} \biggr) \int _{0}^{\kappa _{5}}\bar{\mathcal{H}}_{5}( \boldsymbol{\ell })\frac{E(t-\boldsymbol{\ell })Y_{4}}{E_{4}Y}\,d\boldsymbol{\ell }\\ &\quad {}+\vartheta _{3}S_{4}Y_{4}+\frac{rY_{4}}{\varphi \mathcal{H}_{4}}- \frac{\mu _{1}\pi _{1} ( \gamma +\lambda ) }{\sigma _{1}\mathcal{P}}C^{I} \\ & \quad{} +\frac{\vartheta _{3}S_{4}Y_{4}}{\mathcal{H}_{4}} \int _{0}^{\kappa _{4}}\bar{\mathcal{H}}_{4}(\boldsymbol{\ell })\ln \biggl( \frac{S(t-\boldsymbol{\ell })Y(t-\boldsymbol{\ell })}{SY} \biggr) \,d \boldsymbol{\ell }\\ &\quad {}+ \biggl( \frac{\vartheta _{3}S_{4}Y_{4}}{\mathcal{H}_{5}}+ \frac{rY_{4}}{\varphi \mathcal{H}_{4}\mathcal{H}_{5}} \biggr) \int _{0}^{\kappa _{5}}\bar{\mathcal{H}}_{5}( \boldsymbol{\ell })\ln \biggl( \frac{E(t-\boldsymbol{\ell })}{E} \biggr) \,d \boldsymbol{\ell }\\ &\quad {}+ \frac{a ( \lambda +\gamma ) }{\mathcal{P}} \biggl\{ \frac{\mathcal{P}S_{4} ( \varepsilon \vartheta _{2}+b\vartheta _{1}\mathcal{H}_{6} ) }{a\varepsilon ( \lambda +\gamma ) }-1 \biggr\} I \biggr] . \end{aligned}$$

Using the equalities given by (11) in case of $m=4$, we get

22 $$\begin{aligned} \frac{d\Phi _{4}}{dt} & =-\mathcal{P} \biggl[ \varrho \frac{ ( S-S_{4} ) ^{2}}{S}+ \vartheta _{3}S_{4}Y_{4} \biggl\{ \frac{S_{4}}{S}-1-\ln \biggl( \frac{S_{4}}{S} \biggr) \biggr\} + \frac{rY_{4}}{\varphi \mathcal{H}_{4}} \biggl\{ \frac{YE_{4}}{Y_{4}E}-1- \ln \biggl( \frac{YE_{4}}{Y_{4}E} \biggr) \biggr\} \\ & \quad{} +\frac{\vartheta _{3}S_{4}Y_{4}}{\mathcal{H}_{4}} \int _{0}^{\kappa _{4}}\bar{\mathcal{H}}_{4}(\boldsymbol{\ell }) \biggl\{ \frac{S(t-\boldsymbol{\ell })Y(t-\boldsymbol{\ell })E_{4}}{S_{4}Y_{4}E}-1-\ln \biggl( \frac{S(t-\boldsymbol{\ell })Y(t-\boldsymbol{\ell })E_{4}}{S_{4}Y_{4}E} \biggr) \biggr\} \,d\boldsymbol{\ell } \\ & \quad{} + \biggl( \frac{\vartheta _{3}S_{4}Y_{4}}{\mathcal{H}_{5}}+\frac{rY_{4}}{\varphi \mathcal{H}_{4}\mathcal{H}_{5}} \biggr) \int _{0}^{\kappa _{5}}\bar{\mathcal{H}}_{5}( \boldsymbol{\ell }) \biggl\{ \frac{E(t-\boldsymbol{\ell })Y_{4}}{E_{4}Y}-1-\ln \biggl( \frac{E(t-\boldsymbol{\ell })Y_{4}}{E_{4}Y} \biggr) \biggr\} \,d \boldsymbol{\ell } \\ & \quad{} +\frac{a ( \lambda +\gamma ) }{\mathcal{P}} \biggl\{ \frac{\mathcal{P}S_{4} ( \varepsilon \vartheta _{2}+b\vartheta _{1}\mathcal{H}_{6} ) }{a\varepsilon ( \lambda +\gamma ) }-1 \biggr\} I- \frac{\mu _{1}\pi _{1} ( \gamma +\lambda ) }{\sigma _{1}\mathcal{P}}C^{I} \biggr] . \end{aligned}$$

Therefore, Eq. (22) becomes

$$\begin{aligned} \frac{d\Phi _{4}}{dt} & =-\mathcal{P} \biggl[ \varrho \frac{ ( S-S_{4} ) ^{2}}{S}+ \frac{rY_{4}}{\varphi \mathcal{H}_{4}} \digamma \biggl( \frac{YE_{4}}{Y_{4}E} \biggr) \\ &\quad {}+ \frac{\vartheta _{3}S_{4}Y_{4}}{\mathcal{H}_{4}} \int _{0}^{\kappa _{4}}\bar{\mathcal{H}}_{4}( \boldsymbol{\ell }) \biggl\{ \digamma \biggl( \frac{S(t-\boldsymbol{\ell })Y(t-\boldsymbol{\ell })E_{4}}{S_{4}Y_{4}E} \biggr)+\digamma \biggl( \frac{S_{4}}{S} \biggr) \biggr\} \,d \boldsymbol{\ell } \\ & \quad{}+ \biggl( \frac{\vartheta _{3}S_{4}Y_{4}}{\mathcal{H}_{5}}+\frac{rY_{4}}{\varphi \mathcal{H}_{4}\mathcal{H}_{5}} \biggr) \int _{0}^{\kappa _{5}}\bar{\mathcal{H}}_{5}( \boldsymbol{\ell }) \digamma \biggl( \frac{E(t-\boldsymbol{\ell })Y_{4}}{E_{4}Y} \biggr) \,d \boldsymbol{ \ell } \\ & \quad{} +\frac{a ( \lambda +\gamma ) }{\mathcal{P}} ( \Re _{6}-1 ) I- \frac{\mu _{1}\pi _{1} ( \gamma +\lambda ) }{\sigma _{1}\mathcal{P}}C^{I} \biggr] . \end{aligned}$$

Hence, if $\Re _{6}\leq 1$, then $\frac{d\Phi _{4}}{dt}\leq 0$ for all $S,I,E,Y,V,C^{I}>0$, where $\frac{d\Phi _{4}}{dt}=0$ occurs at $S=S_{4}$, $E=E_{4}$, $Y=Y_{4}$, and $I=C^{I}=0$. The trajectories of system (5) converge to $\Upsilon _{4}^{{\prime }}$ which includes elements with $S(t)=S_{4}$, $E(t)=E_{4}$, $Y(t)=Y_{4}$, and then $\frac{dS(t)}{dt}=\frac{dY(t)}{dt}=0$. The first and fifth equations of system (5) become

$$\begin{aligned} &0 =\frac{dS(t)}{dt}=\eta -\varrho S_{4}-\vartheta _{1}S_{4}V(t)- \vartheta _{3}S_{4}Y_{4}, \\ &0 =\frac{dY(t)}{dt}=\psi \mathcal{H}_{5}E_{4}-\delta Y_{4}-\mu _{2}C^{Y}(t)Y_{4}, \end{aligned}$$

which imply that $V(t)=0$ and $C^{Y}(t)=C_{4}^{Y}$ for all *t*. Moreover, we have $\frac{dI(t)}{dt}=0$, then the third equation of system (5) becomes

$$ 0=\frac{dI(t)}{dt}=\lambda \int _{0}^{\kappa _{3}} \bar{\mathcal{H}}_{3}( \boldsymbol{\ell })L(t-\boldsymbol{\ell })\,d\boldsymbol{\ell }, $$

which yields $L(t)=0$ for all *t*, and then . Applying LLAS theorem, we get  is GAS. □

### Theorem 6

*If* $\Re _{5}>1$, $\Re _{8}\leq 1$, *and* $\Re _{1}/\Re _{2}>1$, *then*  *is GAS*.

### Proof

Define

$$\begin{aligned} \Phi _{5} & =\mathcal{P}S_{5}\digamma \biggl( \frac{S}{S_{5}} \biggr) + \lambda \mathcal{H}_{3}L_{5} \digamma \biggl( \frac{L}{L_{5}} \biggr) + ( \gamma +\lambda ) I_{5}\digamma \biggl( \frac{I}{I_{5}} \biggr) + \frac{\mathcal{P}}{\varphi \mathcal{H}_{4}}E_{5} \digamma \biggl( \frac{E}{E_{5}} \biggr) \\ &\quad{}+ \frac{\mathcal{P} ( \psi +\omega ) }{\varphi \psi \mathcal{H}_{4}\mathcal{H}_{5}}Y_{5}\digamma \biggl( \frac{Y}{Y_{5}} \biggr) + \frac{\mathcal{P}\vartheta _{1}S_{5}}{\varepsilon }V_{5}\digamma \biggl( \frac{V}{V_{5}} \biggr) + \frac{\mu _{1} ( \gamma +\lambda ) }{\sigma _{1}}C_{5}^{I} \digamma \biggl( \frac{C^{I}}{C_{5}^{I}} \biggr) \\ &\quad{}+ \frac{\mu _{2}\mathcal{P} ( \psi +\omega ) }{\sigma _{2}\varphi \psi \mathcal{H}_{4}\mathcal{H}_{5}}C^{Y}+\vartheta _{1} \lambda \mathcal{H}_{3} ( 1-\beta ) S_{5}V_{5} \int _{0}^{\kappa _{1}}\bar{\mathcal{H}}_{1}( \boldsymbol{\ell }) \int _{t-\boldsymbol{\ell }}^{t}\digamma \biggl( \frac{S(\varkappa )V(\varkappa )}{S_{5}V_{5}} \biggr) \,d\varkappa \,d\boldsymbol{\ell } \\ &\quad{}+\vartheta _{2}\lambda \mathcal{H}_{3} ( 1-\beta ) S_{5}I_{5}\int _{0}^{\kappa _{1}}\bar{\mathcal{H}}_{1}( \boldsymbol{\ell }) \int _{t-\boldsymbol{\ell }}^{t}\digamma \biggl( \frac{S(\varkappa )I(\varkappa )}{S_{5}I_{5}} \biggr) \,d\varkappa \,d \boldsymbol{\ell } \\ &\quad{}+\vartheta _{1}\beta ( \gamma + \lambda ) S_{5}V_{5} \int _{0}^{\kappa _{2}}\bar{\mathcal{H}}_{2}( \boldsymbol{\ell }) \int _{t-\boldsymbol{\ell }}^{t}\digamma \biggl( \frac{S(\varkappa )V(\varkappa )}{S_{5}V_{5}} \biggr) \,d\varkappa \,d \boldsymbol{\ell }\\ &\quad {}+\vartheta _{2}\beta ( \gamma + \lambda ) S_{5}I_{5} \int _{0}^{\kappa _{2}}\bar{\mathcal{H}}_{2}( \boldsymbol{\ell }) \int _{t-\boldsymbol{\ell }}^{t}\digamma \biggl( \frac{S(\varkappa )I(\varkappa )}{S_{5}I_{5}} \biggr) \,d\varkappa \,d\boldsymbol{\ell } \\ &\quad{}+\lambda ( \gamma +\lambda ) L_{5} \int _{0}^{ \kappa _{3}}\bar{\mathcal{H}}_{3}( \boldsymbol{\ell }) \int _{t-\boldsymbol{\ell }}^{t}\digamma \biggl( \frac{L(\varkappa )}{L_{5}} \biggr) \,d\varkappa \,d \boldsymbol{\ell }\\ &\quad {}+ \frac{\mathcal{P}\vartheta _{3}S_{5}Y_{5}}{\mathcal{H}_{4}} \int _{0}^{\kappa _{4}}\bar{\mathcal{H}}_{4}( \boldsymbol{\ell }) \int _{t-\boldsymbol{\ell }}^{t}\digamma \biggl( \frac{S(\varkappa )Y(\varkappa )}{S_{5}Y_{5}} \biggr) \,d\varkappa \,d \boldsymbol{\ell } \\ &\quad{}+ \frac{\mathcal{P} ( \psi +\omega ) E_{5}}{\varphi \mathcal{H}_{4}\mathcal{H}_{5}} \int _{0}^{\kappa _{5}} \bar{\mathcal{H}}_{5}( \boldsymbol{\ell }) \int _{t-\boldsymbol{\ell }}^{t}\digamma \biggl( \frac{E(\varkappa )}{E_{5}} \biggr) \,d\varkappa \,d\boldsymbol{\ell }\\ &\quad {}+ \frac{b\mathcal{P}\vartheta _{1}S_{5}I_{5}}{\varepsilon } \int _{0}^{\kappa _{6}}\bar{\mathcal{H}}_{6}( \boldsymbol{\ell }) \int _{t-\boldsymbol{\ell }}^{t}\digamma \biggl( \frac{I(\varkappa )}{I_{5}} \biggr) \,d\varkappa \,d \boldsymbol{\ell }. \end{aligned}$$

Calculate $\frac{d\Phi _{5}}{dt}$ as follows:

23 $$\begin{aligned} \frac{d\Phi _{5}}{dt} & =\mathcal{P} \biggl( 1-\frac{S_{5}}{S} \biggr) ( \eta -\varrho S-\vartheta _{1}SV-\vartheta _{2}SI-\vartheta _{3}SY ) +\lambda \mathcal{H}_{3} \biggl( 1- \frac{L_{5}}{L} \biggr) \\ &\quad{}\times \biggl[ ( 1-\beta ) \int _{0}^{\kappa _{1}}\bar{\mathcal{H}}_{1}( \boldsymbol{\ell })S(t-\boldsymbol{\ell }) \bigl\{ \vartheta _{1}V(t- \boldsymbol{\ell })+\vartheta _{2}I(t-\boldsymbol{\ell }) \bigr\} \,d \boldsymbol{\ell }- ( \lambda +\gamma ) L \biggr] \\ &\quad {} + ( \gamma +\lambda ) \biggl( 1- \frac{I_{5}}{I} \biggr) \biggl[ \beta \int _{0}^{\kappa _{2}} \bar{\mathcal{H}}_{2}( \boldsymbol{\ell })S(t-\boldsymbol{\ell }) \bigl\{ \vartheta _{1}V(t- \boldsymbol{\ell })+\vartheta _{2}I(t-\boldsymbol{\ell }) \bigr\} \,d \boldsymbol{\ell } \\ &\quad {}+\lambda \int _{0}^{\kappa _{3}} \bar{\mathcal{H}}_{3}( \boldsymbol{\ell })L(t-\boldsymbol{\ell })\,d\boldsymbol{\ell }-aI-\mu _{1}C^{I}I \biggr] \\ &\quad{}+\frac{\mathcal{P}}{\varphi \mathcal{H}_{4}} \biggl( 1- \frac{E_{5}}{E} \biggr) \biggl[ \varphi \vartheta _{3} \int _{0}^{\kappa _{4}}\bar{\mathcal{H}}_{4}( \boldsymbol{\ell })S(t-\boldsymbol{\ell })Y(t- \boldsymbol{\ell })\,d\boldsymbol{ \ell }+rY- ( \psi +\omega ) E \biggr] \\ &\quad{}+ \frac{\mathcal{P} ( \psi +\omega ) }{\varphi \psi \mathcal{H}_{4}\mathcal{H}_{5}} \biggl( 1-\frac{Y_{5}}{Y} \biggr) \biggl[ \psi \int _{0}^{\kappa _{5}}\bar{\mathcal{H}}_{5}(\boldsymbol{\ell })E(t-\boldsymbol{\ell })\,d \boldsymbol{ \ell }-\delta Y-\mu _{2}C^{Y}Y \biggr] \\ &\quad {}+ \frac{\mathcal{P}\vartheta _{1}S_{5}}{\varepsilon } \biggl( 1-\frac{V_{5}}{V} \biggr) \biggl[ b \int _{0}^{\kappa _{6}}\bar{\mathcal{H}}_{6}( \boldsymbol{\ell }) I(t-\boldsymbol{\ell })\,d\boldsymbol{\ell }-\varepsilon V \biggr] \\ & \quad{} + \frac{\mu _{1} ( \gamma +\lambda ) }{\sigma _{1}} \biggl( 1- \frac{C_{5}^{I}}{C^{I}} \biggr) \bigl( \sigma _{1}C^{I}I-\pi _{1}C^{I} \bigr) + \frac{\mu _{2}\mathcal{P} ( \psi +\omega ) }{\sigma _{2}\varphi \psi \mathcal{H}_{4}\mathcal{H}_{5}} \bigl( \sigma _{2}C^{Y}Y- \pi _{2}C^{Y} \bigr) \\ &\quad{}+\vartheta _{1}\lambda \mathcal{H}_{3} ( 1-\beta ) S_{5}V_{5} \\ &\quad {}\times\int _{0}^{\kappa _{1}}\bar{\mathcal{H}}_{1}( \boldsymbol{\ell }) \biggl[ \frac{SV}{S_{5}V_{5}}- \frac{S(t-\boldsymbol{\ell })V(t-\boldsymbol{\ell })}{S_{5}V_{5}}+\ln \biggl( \frac{S(t-\boldsymbol{\ell })V(t-\boldsymbol{\ell })}{SV} \biggr) \biggr] \,d\boldsymbol{\ell } \\ &\quad{}+\vartheta _{2}\lambda \mathcal{H}_{3} ( 1-\beta ) S_{5}I_{5} \\ &\quad {}\times\int _{0}^{\kappa _{1}}\bar{\mathcal{H}}_{1}( \boldsymbol{\ell }) \biggl[ \frac{SI}{S_{5}I_{5}}- \frac{S(t-\boldsymbol{\ell })I(t-\boldsymbol{\ell })}{S_{5}I_{5}}+\ln \biggl( \frac{S(t-\boldsymbol{\ell })I(t-\boldsymbol{\ell })}{SI} \biggr) \biggr] \,d\boldsymbol{\ell } \\ &\quad{}+\vartheta _{1}\beta ( \gamma +\lambda ) S_{5}V_{5} \\ &\quad {}\times\int _{0}^{\kappa _{2}}\bar{\mathcal{H}}_{2}( \boldsymbol{\ell }) \biggl[ \frac{SV}{S_{5}V_{5}}- \frac{S(t-\boldsymbol{\ell })V(t-\boldsymbol{\ell })}{S_{5}V_{5}}+ \ln \biggl( \frac{S(t-\boldsymbol{\ell })V(t-\boldsymbol{\ell })}{SV} \biggr) \biggr] \,d\boldsymbol{\ell } \\ &\quad{}+\vartheta _{2}\beta ( \gamma +\lambda ) S_{5}I_{5} \\ &\quad {}\times\int _{0}^{\kappa _{2}}\bar{\mathcal{H}}_{2}( \boldsymbol{\ell }) \biggl[ \frac{SI}{S_{5}I_{5}}- \frac{S(t-\boldsymbol{\ell })I(t-\boldsymbol{\ell })}{S_{5}I_{5}}+ \ln \biggl( \frac{S(t-\boldsymbol{\ell })I(t-\boldsymbol{\ell })}{SI} \biggr) \biggr] \,d\boldsymbol{\ell } \\ &\quad{}+\lambda ( \gamma +\lambda ) L_{5} \int _{0}^{ \kappa _{3}}\bar{\mathcal{H}}_{3}( \boldsymbol{\ell }) \biggl[ \frac{L}{L_{5}}- \frac{L(t-\boldsymbol{\ell })}{L_{5}}+\ln \biggl( \frac{L(t-\boldsymbol{\ell })}{L} \biggr) \biggr] \,d\boldsymbol{\ell } \\ &\quad{}+\frac{\mathcal{P}\vartheta _{3}S_{5}Y_{5}}{\mathcal{H}_{4}} \int _{0}^{\kappa _{4}}\bar{\mathcal{H}}_{4}( \boldsymbol{\ell }) \biggl[ \frac{SY}{S_{5}Y_{5}}- \frac{S(t-\boldsymbol{\ell })Y(t-\boldsymbol{\ell })}{S_{5}Y_{5}}+ \ln \biggl( \frac{S(t-\boldsymbol{\ell })Y(t-\boldsymbol{\ell })}{SY} \biggr) \biggr] \,d\boldsymbol{\ell } \\ &\quad{}+ \frac{\mathcal{P} ( \psi +\omega ) E_{5}}{\varphi \mathcal{H}_{4}\mathcal{H}_{5}} \int _{0}^{\kappa _{5}} \bar{\mathcal{H}}_{5}( \boldsymbol{\ell }) \biggl[ \frac{E}{E_{5}}- \frac{E(t-\boldsymbol{\ell })}{E_{5}}+\ln \biggl( \frac{E(t-\boldsymbol{\ell })}{E} \biggr) \biggr] \,d \boldsymbol{\ell } \\ &\quad{}+\frac{b\mathcal{P}\vartheta _{1}S_{5}I_{5}}{\varepsilon } \int _{0}^{\kappa _{6}}\bar{\mathcal{H}}_{6}(\boldsymbol{\ell }) \biggl[ \frac{I}{I_{5}}- \frac{I(t-\boldsymbol{\ell })}{I_{5}}+\ln \biggl( \frac{I(t-\boldsymbol{\ell })}{I} \biggr) \biggr] \,d\boldsymbol{ \ell }. \end{aligned}$$

Summing the terms of Eq. (23), we get

$$\begin{aligned} \frac{d\Phi _{5}}{dt} & =\mathcal{P} \biggl( 1-\frac{S_{5}}{S} \biggr) ( \eta -\varrho S ) +\mathcal{P}\vartheta _{2}S_{5}I+ \mathcal{P}\vartheta _{3}S_{5}Y-\vartheta _{1}\lambda \mathcal{H}_{3} ( 1- \beta ) \int _{0}^{\kappa _{1}}\bar{\mathcal{H}}_{1}( \boldsymbol{\ell }) \\ &\quad{}\times \frac{S(t-\boldsymbol{\ell })V(t-\boldsymbol{\ell })L_{5}}{L}\,d \boldsymbol{\ell }-\vartheta _{2} \lambda \mathcal{H}_{3} ( 1-\beta ) \int _{0}^{\kappa _{1}}\bar{\mathcal{H}}_{1}( \boldsymbol{\ell })\frac{S(t-\boldsymbol{\ell })I(t-\boldsymbol{\ell })L_{5}}{L}\,d \boldsymbol{\ell } \\ &\quad{}+\lambda \mathcal{H}_{3} ( \lambda +\gamma ) L_{5}-a ( \lambda +\gamma ) I-\vartheta _{1}\beta ( \gamma +\lambda ) \int _{0}^{\kappa _{2}} \bar{\mathcal{H}}_{2}( \boldsymbol{\ell }) \frac{S(t-\boldsymbol{\ell })V(t-\boldsymbol{\ell })I_{5}}{I}\,d\boldsymbol{\ell } \\ &\quad{}-\vartheta _{2}\beta ( \gamma +\lambda ) \int _{0}^{\kappa _{2}}\bar{\mathcal{H}}_{2}(\boldsymbol{\ell }) \frac{S(t-\boldsymbol{\ell })I(t-\boldsymbol{\ell })I_{5}}{I}\,d\boldsymbol{\ell }\\ &\quad {}- \lambda ( \lambda +\gamma ) \int _{0}^{\kappa _{3}} \bar{\mathcal{H}}_{3}( \boldsymbol{\ell })\frac{L(t-\boldsymbol{\ell })I_{5}}{I}\,d\boldsymbol{\ell }+a ( \lambda +\gamma ) I_{5}+\mu _{1} ( \lambda + \gamma ) C^{I}I_{5} \\ &\quad{}+ \frac{\mathcal{P}r}{\varphi \mathcal{H}_{4}}Y- \frac{\mathcal{P}\vartheta _{3}}{\mathcal{H}_{4}} \int _{0}^{ \kappa _{4}}\bar{\mathcal{H}}_{4}( \boldsymbol{\ell }) \frac{S(t-\boldsymbol{\ell })Y(t-\boldsymbol{\ell })E_{5}}{E}\,d\boldsymbol{\ell } \\ &\quad{}-\frac{\mathcal{P}r}{\varphi \mathcal{H}_{4}}\frac{YE_{5}}{E}+ \frac{\mathcal{P} ( \psi +\omega ) }{\varphi \mathcal{H}_{4}}E_{5}- \frac{\mathcal{P}\delta ( \psi +\omega ) }{\varphi \psi \mathcal{H}_{4}\mathcal{H}_{5}}Y\\ &\quad {}- \frac{\mathcal{P} ( \psi +\omega ) }{\varphi \mathcal{H}_{4}\mathcal{H}_{5}} \int _{0}^{\kappa _{5}}\bar{\mathcal{H}}_{5}( \boldsymbol{\ell }) \frac{E(t-\boldsymbol{\ell })Y_{5}}{Y}\,d\boldsymbol{\ell }+ \frac{\mathcal{P}\delta ( \psi +\omega ) }{\varphi \psi \mathcal{H}_{4}\mathcal{H}_{5}}Y_{5} \\ &\quad{}+ \frac{\mu _{2}\mathcal{P} ( \psi +\omega ) }{\varphi \psi \mathcal{H}_{4}\mathcal{H}_{5}}C^{Y}Y_{5}- \frac{b\mathcal{P}\vartheta _{1}S_{5}}{\varepsilon } \int _{0}^{\kappa _{6}}\bar{\mathcal{H}}_{6}(\boldsymbol{\ell }) \frac{I(t-\boldsymbol{\ell })V_{5}}{V}\,d\boldsymbol{\ell }+\mathcal{P}\vartheta _{1}S_{5}V_{5} \\ &\quad{}- \frac{\mu _{1}\pi _{1} ( \gamma +\lambda ) }{\sigma _{1}}C^{I}-\mu _{1} ( \lambda +\gamma ) C_{5}^{I}I+ \frac{\mu _{1}\pi _{1} ( \lambda +\gamma ) }{\sigma _{1}}C_{5}^{I}- \frac{\mu _{2}\pi _{2}\mathcal{P} ( \psi +\omega ) }{\sigma _{2}\varphi \psi \mathcal{H}_{4}\mathcal{H}_{5}}C^{Y} \\ &\quad{}+\lambda \mathcal{H}_{3} ( 1-\beta ) \vartheta _{1}S_{5}V_{5}\int _{0}^{\kappa _{1}}\bar{\mathcal{H}}_{1}( \boldsymbol{\ell }) \ln \biggl( \frac{S(t-\boldsymbol{\ell })V(t-\boldsymbol{\ell })}{SV} \biggr) \,d \boldsymbol{\ell }\\ &\quad{}+\lambda \mathcal{H}_{3} ( 1-\beta ) \vartheta _{2}S_{5}I_{5}\int _{0}^{\kappa _{1}}\bar{\mathcal{H}}_{1}( \boldsymbol{\ell }) \ln \biggl( \frac{S(t-\boldsymbol{\ell })I(t-\boldsymbol{\ell })}{SI} \biggr) \,d\boldsymbol{\ell }\\ &\quad {}+\beta ( \gamma +\lambda ) \vartheta _{1}S_{5}V_{5}\int _{0}^{\kappa _{2}}\bar{\mathcal{H}}_{2}( \boldsymbol{\ell }) \ln \biggl( \frac{S(t-\boldsymbol{\ell })V(t-\boldsymbol{\ell })}{SV} \biggr) \,d \boldsymbol{\ell } \\ &\quad{}+\beta ( \gamma +\lambda ) \vartheta _{2}S_{5}I_{5} \int _{0}^{\kappa _{2}}\bar{\mathcal{H}}_{2}( \boldsymbol{\ell }) \ln \biggl( \frac{S(t-\boldsymbol{\ell })I(t-\boldsymbol{\ell })}{SI} \biggr) \,d \boldsymbol{\ell }\\ &\quad {}+\lambda ( \gamma +\lambda ) L_{5} \int _{0}^{ \kappa _{3}}\bar{\mathcal{H}}_{3}( \boldsymbol{\ell })\ln \biggl( \frac{L(t-\boldsymbol{\ell })}{L} \biggr) \,d\boldsymbol{\ell } \\ &\quad{}+\frac{\mathcal{P}\vartheta _{3}S_{5}Y_{5}}{\mathcal{H}_{4}} \int _{0}^{\kappa _{4}}\bar{\mathcal{H}}_{4}( \boldsymbol{\ell })\ln \biggl( \frac{S(t-\boldsymbol{\ell })Y(t-\boldsymbol{\ell })}{SY} \biggr) \,d \boldsymbol{\ell }\\ &\quad {}+ \frac{\mathcal{P} ( \psi +\omega ) E_{5}}{\varphi \mathcal{H}_{4}\mathcal{H}_{5}} \int _{0}^{\kappa _{5}} \bar{\mathcal{H}}_{5}( \boldsymbol{\ell })\ln \biggl( \frac{E(t-\boldsymbol{\ell })}{E} \biggr) \,d \boldsymbol{\ell } \\ &\quad{}+ \frac{b\mathcal{P}\vartheta _{1}S_{5}\mathcal{H}_{6}}{\varepsilon }I+\frac{b\mathcal{P}\vartheta _{1}S_{5}I_{5}}{\varepsilon } \int _{0}^{\kappa _{6}}\bar{\mathcal{H}}_{6}(\boldsymbol{\ell })\ln \biggl( \frac{I(t-\boldsymbol{\ell })}{I} \biggr) \,d\boldsymbol{\ell }. \end{aligned}$$

Using the steady state conditions for

$$\begin{aligned} & \eta =\varrho S_{5}+\vartheta _{1}S_{5}V_{5}+ \vartheta _{2}S_{5}I_{5}+\vartheta _{3}S_{5}Y_{5}, \qquad \mathcal{H}_{1} ( 1-\beta ) ( \vartheta _{1}S_{5}V_{5}+\vartheta _{2}S_{5}I_{5} ) = ( \lambda +\gamma ) L_{5}, \\ & \beta \mathcal{H}_{2} ( \vartheta _{1}S_{5}V_{5}+ \vartheta _{2}S_{5}I_{5} ) +\lambda \mathcal{H}_{3}L_{5}= \bigl( a+\mu _{1}C_{5}^{I} \bigr) I_{5} \qquad I_{5}= \frac{\pi _{1}}{\sigma _{1}}, \qquad V_{5}= \frac{b\mathcal{H}_{6}}{\varepsilon }I_{5}, \\ & \vartheta _{3}S_{5}Y_{5}+ \frac{rY_{5}}{\varphi \mathcal{H}_{4}}=\frac{ ( \psi +\omega ) E_{5}}{\varphi \mathcal{H}_{4}}= \frac{\delta ( \psi +\omega ) Y_{5}}{\varphi \psi \mathcal{H}_{4}\mathcal{H}_{5}}, \end{aligned}$$

we obtain

$$ \mathcal{P} ( \vartheta _{1}S_{5}V_{5}+ \vartheta _{2}S_{5}I_{5} ) = ( \lambda +\gamma ) \bigl( a+\mu _{1}C_{5}^{I} \bigr) I_{5}. $$

Moreover, we get

$$\begin{aligned} \frac{d\Phi _{5}}{dt} & =\mathcal{P} \biggl( 1-\frac{S_{5}}{S} \biggr) ( \varrho S_{5}-\varrho S ) +\mathcal{P} ( \vartheta _{1}S_{5}V_{5}+ \vartheta _{2}S_{5}I_{5}+\vartheta _{3}S_{5}Y_{5} ) \biggl( 1-\frac{S_{5}}{S} \biggr) \\ &\quad{}-\lambda \mathcal{H}_{3} ( 1-\beta ) \vartheta _{1}S_{5}V_{5}\int _{0}^{\kappa _{1}}\bar{\mathcal{H}}_{1}( \boldsymbol{\ell }) \frac{S(t-\boldsymbol{\ell })V(t-\boldsymbol{\ell })L_{5}}{S_{5}V_{5}L}\,d \boldsymbol{\ell }\\ &\quad {} -\lambda \mathcal{H}_{3} ( 1-\beta ) \vartheta _{2}S_{5}I_{5} \int _{0}^{\kappa _{1}}\bar{\mathcal{H}}_{1}( \boldsymbol{\ell })\frac{S(t-\boldsymbol{\ell })I(t-\boldsymbol{\ell })L_{5}}{S_{5}I_{5}L}\,d \boldsymbol{\ell }\\ &\quad {}+\lambda \mathcal{H}_{1}\mathcal{H}_{3} ( 1-\beta ) ( \vartheta _{1}S_{5}V_{5}+\vartheta _{2}S_{5}I_{5} ) \\ &\quad{}-\beta ( \gamma +\lambda ) \vartheta _{1}S_{5}V_{5} \int _{0}^{\kappa _{2}}\bar{\mathcal{H}}_{2}( \boldsymbol{\ell }) \frac{S(t-\boldsymbol{\ell })V(t-\boldsymbol{\ell })I_{5}}{S_{5}V_{5}I}\,d \boldsymbol{\ell }\\ &\quad {}-\beta ( \gamma +\lambda ) \vartheta _{2}S_{5}I_{5} \int _{0}^{\kappa _{2}}\bar{\mathcal{H}}_{2}( \boldsymbol{\ell })\frac{S(t-\boldsymbol{\ell })I(t-\boldsymbol{\ell })}{S_{5}I}\,d\boldsymbol{\ell }\\ &\quad {}- \lambda \mathcal{H}_{1} ( 1-\beta ) ( \vartheta _{1}S_{5}V_{5}+ \vartheta _{2}S_{5}I_{5} ) \int _{0}^{\kappa _{3}}\bar{\mathcal{H}}_{3}( \boldsymbol{\ell })\frac{L(t-\boldsymbol{\ell })I_{5}}{L_{5}I}\,d\boldsymbol{\ell }\\ &\quad {}+\mathcal{P} ( \vartheta _{1}S_{5}V_{5}+\vartheta _{2}S_{5}I_{5} ) - \frac{\mathcal{P}\vartheta _{3}S_{5}Y_{5}}{\mathcal{H}_{4}} \int _{0}^{\kappa _{4}}\bar{\mathcal{H}}_{4}( \boldsymbol{\ell }) \frac{S(t-\boldsymbol{\ell })Y(t-\boldsymbol{\ell })E_{5}}{S_{5}Y_{5}E}\,d\boldsymbol{\ell }\\ &\quad {}-\frac{\mathcal{P}rY_{5}}{\varphi \mathcal{H}_{4}} \frac{YE_{5}}{Y_{5}E}+\mathcal{P}\vartheta _{3}S_{5}Y_{5}+ \frac{\mathcal{P}rY_{5}}{\varphi \mathcal{H}_{4}} \\ &\quad{}- \biggl( \frac{\mathcal{P}\vartheta _{3}S_{5}Y_{5}}{\mathcal{H}_{5}}+\frac{\mathcal{P}rY_{5}}{\varphi \mathcal{H}_{4}\mathcal{H}_{5}} \biggr) \int _{0}^{\kappa _{5}}\bar{\mathcal{H}}_{5}( \boldsymbol{\ell })\frac{E(t-\boldsymbol{\ell })Y_{5}}{E_{5}Y}\,d\boldsymbol{\ell }+ \mathcal{P}\vartheta _{3}S_{5}Y_{5}+\frac{\mathcal{P}rY_{5}}{\varphi \mathcal{H}_{4}} \\ &\quad{}-\frac{\mathcal{P}\vartheta _{1}S_{5}V_{5}}{\mathcal{H}_{6}} \int _{0}^{\kappa _{6}}\bar{\mathcal{H}}_{6}( \boldsymbol{\ell }) \frac{I(t-\boldsymbol{\ell })V_{5}}{I_{5}V}\,d\boldsymbol{\ell }+\mathcal{P} \vartheta _{1}S_{5}V_{5}\\ &\quad {}+\lambda \mathcal{H}_{3} ( 1-\beta ) \vartheta _{1}S_{5}V_{5} \int _{0}^{\kappa _{1}}\bar{\mathcal{H}}_{1}( \boldsymbol{\ell })\ln \biggl( \frac{S(t-\boldsymbol{\ell })V(t-\boldsymbol{\ell })}{SV} \biggr) \,d \boldsymbol{\ell } \\ &\quad{}+\lambda \mathcal{H}_{3} ( 1-\beta ) \vartheta _{2}S_{5}I_{5} \int _{0}^{\kappa _{1}} \bar{\mathcal{H}}_{1}( \boldsymbol{\ell }) \ln \biggl( \frac{S(t-\boldsymbol{\ell })I(t-\boldsymbol{\ell })}{SI} \biggr) \,d\boldsymbol{\ell }\\ &\quad {}+\beta ( \gamma +\lambda ) \vartheta _{1}S_{5}V_{5}\int _{0}^{\kappa _{2}}\bar{\mathcal{H}}_{2}( \boldsymbol{\ell }) \ln \biggl( \frac{S(t-\boldsymbol{\ell })V(t-\boldsymbol{\ell })}{SV} \biggr) \,d \boldsymbol{\ell } \\ &\quad{}+\beta ( \gamma +\lambda ) \vartheta _{2}S_{5}I_{5} \int _{0}^{\kappa _{2}}\bar{\mathcal{H}}_{2}( \boldsymbol{\ell }) \ln \biggl( \frac{S(t-\boldsymbol{\ell })I(t-\boldsymbol{\ell })}{SI} \biggr) \,d \boldsymbol{\ell }\\ &\quad {}+\lambda \mathcal{H}_{1} ( 1-\beta ) ( \vartheta _{1}S_{5}V_{5}+ \vartheta _{2}S_{5}I_{5} ) \int _{0}^{\kappa _{3}}\bar{\mathcal{H}}_{3}( \boldsymbol{\ell })\ln \biggl( \frac{L(t-\boldsymbol{\ell })}{L} \biggr) \,d \boldsymbol{\ell } \\ &\quad{}+ \frac{\mathcal{P}\vartheta _{3}S_{5}Y_{5}}{\mathcal{H}_{4}} \int _{0}^{\kappa _{4}}\bar{\mathcal{H}}_{4}( \boldsymbol{\ell }) \ln \biggl( \frac{S(t-\boldsymbol{\ell })Y(t-\boldsymbol{\ell })}{SY} \biggr) \,d\boldsymbol{\ell }\\ &\quad {}+ \biggl( \frac{\mathcal{P}\vartheta _{3}S_{5}Y_{5}}{\mathcal{H}_{5}}+ \frac{\mathcal{P}rY_{5}}{\varphi \mathcal{H}_{4}\mathcal{H}_{5}} \biggr) \int _{0}^{\kappa _{5}} \bar{\mathcal{H}}_{5}( \boldsymbol{\ell })\ln \biggl( \frac{E(t-\boldsymbol{\ell })}{E} \biggr) \,d \boldsymbol{\ell } \\ &\quad{}+\frac{\mathcal{P}\vartheta _{1}S_{5}V_{5}}{\mathcal{H}_{6}} \int _{0}^{\kappa _{6}}\bar{\mathcal{H}}_{6}( \boldsymbol{\ell })\ln \biggl( \frac{I(t-\boldsymbol{\ell })}{I} \biggr) \,d\boldsymbol{\ell }+ \frac{\mu _{2}\mathcal{P} ( \psi +\omega ) }{\varphi \psi \mathcal{H}_{4}\mathcal{H}_{5}} \biggl( Y_{5}-\frac{\pi _{2}}{\sigma _{2}} \biggr) C^{Y}. \end{aligned}$$

Using the equalities given by (10) and (11) in case of $n=m=5$, we get

24 $$\begin{aligned} \frac{d\Phi _{5}}{dt} & =-\varrho \mathcal{P} \frac{(S-S_{5})^{2}}{S}-\mathcal{P} ( \vartheta _{1}S_{5}V_{5}+\vartheta _{2}S_{5}I_{5}+\vartheta _{3}S_{5}Y_{5} ) \biggl[ \frac{S_{5}}{S}-1- \ln \biggl( \frac{S_{5}}{S} \biggr) \biggr] \\ &\quad{}-\lambda \mathcal{H}_{3} ( 1-\beta ) \vartheta _{1}S_{5}V_{5} \\ &\quad {}\times\int _{0}^{\kappa _{1}}\bar{\mathcal{H}}_{1}( \boldsymbol{\ell }) \biggl[ \frac{S(t-\boldsymbol{\ell })V(t-\boldsymbol{\ell })L_{5}}{S_{5}V_{5}L}-1- \ln \biggl( \frac{S(t-\boldsymbol{\ell })V(t-\boldsymbol{\ell })L_{5}}{S_{5}V_{5}L} \biggr) \biggr] \,d\boldsymbol{\ell } \\ &\quad{}-\lambda \mathcal{H}_{3} ( 1-\beta ) \vartheta _{2}S_{5}I_{5} \\ &\quad {}\times\int _{0}^{\kappa _{1}}\bar{\mathcal{H}}_{1}( \boldsymbol{\ell }) \biggl[ \frac{S(t-\boldsymbol{\ell })I(t-\boldsymbol{\ell })L_{5}}{S_{5}I_{5}L}-1- \ln \biggl( \frac{S(t-\boldsymbol{\ell })I(t-\boldsymbol{\ell })L_{5}}{S_{5}I_{5}L} \biggr) \biggr] \,d\boldsymbol{\ell } \\ &\quad{}-\beta ( \gamma +\lambda ) \vartheta _{1}S_{5}V_{5} \\ &\quad {}\times\int _{0}^{\kappa _{2}}\bar{\mathcal{H}}_{2}( \boldsymbol{\ell }) \biggl[ \frac{S(t-\boldsymbol{\ell })V(t-\boldsymbol{\ell })I_{5}}{S_{5}V_{5}I}-1- \ln \biggl( \frac{S(t-\boldsymbol{\ell })V(t-\boldsymbol{\ell })I_{5}}{S_{5}V_{5}I} \biggr) \biggr] \,d\boldsymbol{\ell } \\ &\quad{}-\beta ( \gamma +\lambda ) \vartheta _{2}S_{5}I_{5} \int _{0}^{\kappa _{2}}\bar{\mathcal{H}}_{2}( \boldsymbol{\ell }) \biggl[ \frac{S(t-\boldsymbol{\ell })I(t-\boldsymbol{\ell })}{S_{5}I}-1-\ln \biggl( \frac{S(t-\boldsymbol{\ell })I(t-\boldsymbol{\ell })}{S_{5}I} \biggr) \biggr] \,d\boldsymbol{\ell } \\ &\quad{}-\lambda \mathcal{H}_{1} ( 1-\beta ) ( \vartheta _{1}S_{5}V_{5}+\vartheta _{2}S_{5}I_{5} ) \\ &\quad {}\times\int _{0}^{ \kappa _{3}}\bar{\mathcal{H}}_{3}( \boldsymbol{\ell }) \biggl[ \frac{L(t-\boldsymbol{\ell })I_{5}}{L_{5}I}-1-\ln \biggl( \frac{L(t-\boldsymbol{\ell })I_{5}}{L_{5}I} \biggr) \biggr] \,d\boldsymbol{\ell } \\ &\quad{}-\frac{\mathcal{P}rY_{5}}{\varphi \mathcal{H}_{4}} \biggl[ \frac{YE_{5}}{Y_{5}E}-1-\ln \biggl( \frac{YE_{5}}{Y_{5}E} \biggr) \biggr] \\ & \quad{}- \frac{\mathcal{P}\vartheta _{3}S_{5}Y_{5}}{\mathcal{H}_{4}} \int _{0}^{\kappa _{4}}\bar{\mathcal{H}}_{4}(\boldsymbol{\ell }) \biggl[ \frac{S(t-\boldsymbol{\ell })Y(t-\boldsymbol{\ell })E_{5}}{S_{5}Y_{5}E}-1 -\ln \biggl( \frac{S(t-\boldsymbol{\ell })Y(t-\boldsymbol{\ell })E_{5}}{S_{5}Y_{5}E} \biggr) \biggr] \,d\boldsymbol{\ell } \\ & \quad{} - \biggl( \frac{\mathcal{P}\vartheta _{3}S_{5}Y_{5}}{\mathcal{H}_{5}}+ \frac{\mathcal{P}rY_{5}}{\varphi \mathcal{H}_{4}\mathcal{H}_{5}} \biggr) \int _{0}^{ \kappa _{5}}\bar{\mathcal{H}}_{5}( \boldsymbol{\ell }) \biggl[ \frac{E(t-\boldsymbol{\ell })Y_{5}}{E_{5}Y}-1-\ln \biggl( \frac{E(t-\boldsymbol{\ell })Y_{5}}{E_{5}Y} \biggr) \biggr] \,d\boldsymbol{\ell } \\ &\quad {}- \frac{\mathcal{P}\vartheta _{1}S_{5}V_{5}}{\mathcal{H}_{6}}\int _{0}^{\kappa _{6}}\bar{\mathcal{H}}_{6}( \boldsymbol{\ell }) \biggl[ \frac{I(t-\boldsymbol{\ell })V_{5}}{I_{5}V}-1-\ln \biggl( \frac{I(t-\boldsymbol{\ell })V_{5}}{I_{5}V} \biggr) \biggr] \,d \boldsymbol{\ell } \\ &\quad{}+ \frac{\mu _{2}\mathcal{P} ( \psi +\omega ) }{\varphi \psi \mathcal{H}_{4}\mathcal{H}_{5}} \biggl( Y_{5}-\frac{\pi _{2}}{\sigma _{2}} \biggr) C^{Y}. \end{aligned}$$

Therefore, Eq. (24) becomes

$$\begin{aligned} \frac{d\Phi _{5}}{dt} & =-\varrho \mathcal{P} \frac{(S-S_{5})^{2}}{S}-\mathcal{P} ( \vartheta _{1}S_{5}V_{5}+\vartheta _{2}S_{5}I_{5}+\vartheta _{3}S_{5}Y_{5} ) \digamma \biggl( \frac{S_{5}}{S} \biggr) \\ &\quad{}-\lambda \mathcal{H}_{3} ( 1-\beta ) \vartheta _{1}S_{5}V_{5}\int _{0}^{\kappa _{1}}\bar{\mathcal{H}}_{1}( \boldsymbol{\ell }) \digamma \biggl( \frac{S(t-\boldsymbol{\ell })V(t-\boldsymbol{\ell })L_{5}}{S_{5}V_{5}L} \biggr) \,d \boldsymbol{ \ell } \\ &\quad{}-\lambda \mathcal{H}_{3} ( 1-\beta ) \vartheta _{2}S_{5}I_{5}\int _{0}^{\kappa _{1}}\bar{\mathcal{H}}_{1}( \boldsymbol{\ell }) \digamma \biggl( \frac{S(t-\boldsymbol{\ell })I(t-\boldsymbol{\ell })L_{5}}{S_{5}I_{5}L} \biggr) \,d \boldsymbol{ \ell } \\ &\quad{}-\beta ( \gamma +\lambda ) \vartheta _{1}S_{5}V_{5} \int _{0}^{\kappa _{2}}\bar{\mathcal{H}}_{2}( \boldsymbol{\ell }) \digamma \biggl( \frac{S(t-\boldsymbol{\ell })V(t-\boldsymbol{\ell })I_{5}}{S_{5}V_{5}I} \biggr) \,d \boldsymbol{ \ell } \\ &\quad{}-\beta ( \gamma +\lambda ) \vartheta _{2}S_{5}I_{5} \int _{0}^{\kappa _{2}}\bar{\mathcal{H}}_{2}( \boldsymbol{\ell }) \digamma \biggl( \frac{S(t-\boldsymbol{\ell })I(t-\boldsymbol{\ell })}{S_{5}I} \biggr) \,d\boldsymbol{\ell } \\ &\quad{}-\lambda \mathcal{H}_{1} ( 1-\beta ) ( \vartheta _{1}S_{5}V_{5}+\vartheta _{2}S_{5}I_{5} ) \int _{0}^{ \kappa _{3}}\bar{\mathcal{H}}_{3}( \boldsymbol{\ell })\digamma \biggl( \frac{L(t-\boldsymbol{\ell })I_{5}}{L_{5}I} \biggr) \,d\boldsymbol{\ell } \\ &\quad{}-\frac{\mathcal{P}rY_{5}}{\varphi \mathcal{H}_{4}}\digamma \biggl( \frac{YE_{5}}{Y_{5}E} \biggr) - \frac{\mathcal{P}\vartheta _{3}S_{5}Y_{5}}{\mathcal{H}_{4}} \int _{0}^{\kappa _{4}}\bar{\mathcal{H}}_{4}( \boldsymbol{\ell })\digamma \biggl( \frac{S(t-\boldsymbol{\ell })Y(t-\boldsymbol{\ell })E_{5}}{S_{5}Y_{5}E} \biggr) \,d\boldsymbol{\ell } \\ &\quad{}- \biggl( \frac{\mathcal{P}\vartheta _{3}S_{5}Y_{5}}{\mathcal{H}_{5}}+\frac{\mathcal{P}rY_{5}}{\varphi \mathcal{H}_{4}\mathcal{H}_{5}} \biggr) \int _{0}^{\kappa _{5}}\bar{\mathcal{H}}_{5}( \boldsymbol{\ell })\digamma \biggl( \frac{E(t-\boldsymbol{\ell })Y_{5}}{E_{5}Y} \biggr) \,d\boldsymbol{\ell } \\ &\quad{}-\frac{\mathcal{P}\vartheta _{1}S_{5}V_{5}}{\mathcal{H}_{6}} \int _{0}^{\kappa _{6}}\bar{\mathcal{H}}_{6}( \boldsymbol{\ell }) \digamma \biggl( \frac{I(t-\boldsymbol{\ell })V_{5}}{I_{5}V} \biggr) \,d \boldsymbol{ \ell } \\ &\quad{}+ \frac{\mu _{2}\mathcal{P} ( \psi +\omega ) [ \pi _{1}\sigma _{2} ( b\vartheta _{1}\mathcal{H}_{6}+\varepsilon \vartheta _{2} ) +\varepsilon \sigma _{1} ( \pi _{2}\vartheta _{3}+\varrho \sigma _{2} ) ] }{\varphi \psi \vartheta _{3}\varepsilon \sigma _{1}\sigma _{2}\mathcal{H}_{4}\mathcal{H}_{5}} ( \Re _{8}-1 ) C^{Y}. \end{aligned}$$

Hence, if $\Re _{8}\leq 1$, then $\frac{d\Phi _{5}}{dt}\leq 0$ for all $S,L,I,E,Y,V,C^{Y}>0$. One can show that $\frac{d\Phi _{5}}{dt}=0$ when $(S,L,I,E,Y,V,C^{Y})=(S_{5},L_{5},I_{5},E_{5},Y_{5},V_{5},0)$. The solutions of model (5) tend to $\Upsilon _{5}^{{\prime }}$ which includes elements with $(S(t),L(t),I(t),V(t))=(S_{5},L_{5},I_{5},V_{5})$, and then $\frac{dI(t)}{dt}=0$. The third equation of system (5) becomes

$$ 0=\frac{dI(t)}{dt}=\beta \mathcal{H}_{2} ( \vartheta _{1}S_{5}V_{5}+\vartheta _{2}S_{5}I_{5} ) +\lambda \mathcal{H}_{3}L_{5}-aI_{5}- \mu _{1}C^{I}(t)I_{5}, $$

which yields $C^{I}(t)=C_{5}^{I}$ for all *t*, and hence . Applying LLAS theorem, we get  is GAS. □

### Theorem 7

*If* $\Re _{6}>1$, $\Re _{7}\leq 1$, *and* $\Re _{2}/\Re _{1}>1$, *then*  *is GAS*.

### Proof

Define

$$\begin{aligned} \Phi _{6} & =\mathcal{P}S_{6}\digamma \biggl( \frac{S}{S_{6}} \biggr) + \lambda \mathcal{H}_{3}L_{6} \digamma \biggl( \frac{L}{L_{6}} \biggr) + ( \gamma +\lambda ) I_{6}\digamma \biggl( \frac{I}{I_{6}} \biggr) + \frac{\mathcal{P}}{\varphi \mathcal{H}_{4}}E_{6} \digamma \biggl( \frac{E}{E_{6}} \biggr) \\ &\quad{}+ \frac{\mathcal{P} ( \psi +\omega ) }{\varphi \psi \mathcal{H}_{4}\mathcal{H}_{5}}Y_{6}\digamma \biggl( \frac{Y}{Y_{6}} \biggr) + \frac{\mathcal{P}\vartheta _{1}S_{6}}{\varepsilon }V_{6}\digamma \biggl( \frac{V}{V_{6}} \biggr) + \frac{\mu _{1} ( \gamma +\lambda ) }{\sigma _{1}}C^{I} \\ &\quad{}+ \frac{\mu _{2}\mathcal{P} ( \psi +\omega ) }{\sigma _{2}\varphi \psi \mathcal{H}_{4}\mathcal{H}_{5}}C_{6}^{Y}\digamma \biggl( \frac{C^{Y}}{C_{6}^{Y}} \biggr) \\ &\quad {}+\vartheta _{1}\lambda \mathcal{H}_{3} ( 1-\beta ) S_{6}V_{6} \int _{0}^{\kappa _{1}} \bar{\mathcal{H}}_{1}( \boldsymbol{\ell }) \int _{t-\boldsymbol{\ell }}^{t}\digamma \biggl( \frac{S(\varkappa )V(\varkappa )}{S_{6}V_{6}} \biggr) \,d\varkappa \,d \boldsymbol{\ell } \\ &\quad{}+\vartheta _{2}\lambda \mathcal{H}_{3} ( 1-\beta ) S_{6}I_{6}\int _{0}^{\kappa _{1}}\bar{\mathcal{H}}_{1}( \boldsymbol{\ell }) \int _{t-\boldsymbol{\ell }}^{t}\digamma \biggl( \frac{S(\varkappa )I(\varkappa )}{S_{6}I_{6}} \biggr) \,d\varkappa \,d \boldsymbol{\ell } \\ &\quad{}+\vartheta _{1}\beta ( \gamma + \lambda ) S_{6}V_{6} \int _{0}^{\kappa _{2}}\bar{\mathcal{H}}_{2}( \boldsymbol{\ell }) \int _{t-\boldsymbol{\ell }}^{t}\digamma \biggl( \frac{S(\varkappa )V(\varkappa )}{S_{6}V_{6}} \biggr) \,d\varkappa \,d \boldsymbol{\ell }\\ &\quad {}+\vartheta _{2}\beta ( \gamma + \lambda ) S_{6}I_{6} \int _{0}^{\kappa _{2}}\bar{\mathcal{H}}_{2}( \boldsymbol{\ell }) \int _{t-\boldsymbol{\ell }}^{t}\digamma \biggl( \frac{S(\varkappa )I(\varkappa )}{S_{6}I_{6}} \biggr) \,d\varkappa \,d\boldsymbol{\ell } \\ &\quad{}+\lambda ( \gamma +\lambda ) L_{6} \int _{0}^{ \kappa _{3}}\bar{\mathcal{H}}_{3}( \boldsymbol{\ell }) \int _{t-\boldsymbol{\ell }}^{t}\digamma \biggl( \frac{L(\varkappa )}{L_{6}} \biggr) \,d\varkappa \,d \boldsymbol{\ell }\\ &\quad {}+ \frac{\mathcal{P}\vartheta _{3}S_{6}Y_{6}}{\mathcal{H}_{4}} \int _{0}^{\kappa _{4}}\bar{\mathcal{H}}_{4}( \boldsymbol{\ell }) \int _{t-\boldsymbol{\ell }}^{t}\digamma \biggl( \frac{S(\varkappa )Y(\varkappa )}{S_{6}Y_{6}} \biggr) \,d\varkappa \,d \boldsymbol{\ell } \\ &\quad{}+ \frac{\mathcal{P} ( \psi +\omega ) E_{6}}{\varphi \mathcal{H}_{4}\mathcal{H}_{5}} \int _{0}^{\kappa _{5}} \bar{\mathcal{H}}_{5}( \boldsymbol{\ell }) \int _{t-\boldsymbol{\ell }}^{t}\digamma \biggl( \frac{E(\varkappa )}{E_{6}} \biggr) \,d\varkappa \,d\boldsymbol{\ell }\\ &\quad {}+ \frac{b\mathcal{P}\vartheta _{1}S_{6}I_{6}}{\varepsilon } \int _{0}^{\kappa _{6}}\bar{\mathcal{H}}_{6}( \boldsymbol{\ell }) \int _{t-\boldsymbol{\ell }}^{t}\digamma \biggl( \frac{I(\varkappa )}{I_{6}} \biggr) \,d\varkappa \,d \boldsymbol{\ell }. \end{aligned}$$

Calculate $\frac{d\Phi _{6}}{dt}$ as follows:

25 $$\begin{aligned} \frac{d\Phi _{6}}{dt} & =\mathcal{P} \biggl( 1-\frac{S_{6}}{S} \biggr) ( \eta -\varrho S-\vartheta _{1}SV-\vartheta _{2}SI-\vartheta _{3}SY ) +\lambda \mathcal{H}_{3} \biggl( 1- \frac{L_{6}}{L} \biggr) \\ &\quad{}\times \biggl[ ( 1-\beta ) \int _{0}^{\kappa _{1}}\bar{\mathcal{H}}_{1}( \boldsymbol{\ell })S(t-\boldsymbol{\ell }) \bigl\{ \vartheta _{1}V(t- \boldsymbol{\ell })+\vartheta _{2}I(t-\boldsymbol{\ell }) \bigr\} \,d \boldsymbol{\ell }- ( \lambda +\gamma ) L \biggr] \\ &\quad {} + ( \gamma +\lambda ) \biggl( 1- \frac{I_{6}}{I} \biggr)\biggl[ \beta \int _{0}^{\kappa _{2}} \bar{\mathcal{H}}_{2}( \boldsymbol{\ell })S(t-\boldsymbol{\ell }) \bigl\{ \vartheta _{1}V(t- \boldsymbol{\ell })+\vartheta _{2}I(t-\boldsymbol{\ell }) \bigr\} \,d \boldsymbol{\ell } \\ &\quad{}+\lambda \int _{0}^{\kappa _{3}} \bar{\mathcal{H}}_{3}( \boldsymbol{\ell })L(t-\boldsymbol{\ell })\,d\boldsymbol{\ell }-aI-\mu _{1}C^{I}I \biggr] \\ &\quad{}+\frac{\mathcal{P}}{\varphi \mathcal{H}_{4}} \biggl( 1- \frac{E_{6}}{E} \biggr) \biggl[ \varphi \vartheta _{3} \int _{0}^{\kappa _{4}}\bar{\mathcal{H}}_{4}( \boldsymbol{\ell })S(t-\boldsymbol{\ell })Y(t- \boldsymbol{\ell })\,d\boldsymbol{ \ell }+rY- ( \psi +\omega ) E \biggr] \\ &\quad {} + \frac{\mathcal{P} ( \psi +\omega ) }{\varphi \psi \mathcal{H}_{4}\mathcal{H}_{5}}\biggl( 1-\frac{Y_{6}}{Y} \biggr) \biggl[ \psi \int _{0}^{\kappa _{5}}\bar{\mathcal{H}}_{5}(\boldsymbol{\ell })E(t-\boldsymbol{\ell })\,d \boldsymbol{ \ell }-\delta Y-\mu _{2}C^{Y}Y \biggr] \\ &\quad{} + \frac{\mathcal{P}\vartheta _{1}S_{6}}{\varepsilon } \biggl( 1-\frac{V_{6}}{V} \biggr)\biggl[ b \int _{0}^{\kappa _{6}}\bar{\mathcal{H}}_{6}( \boldsymbol{\ell })I(t-\boldsymbol{\ell })\,d\boldsymbol{\ell }-\varepsilon V \biggr] \\ &\quad{}+ \frac{\mu _{1} ( \gamma +\lambda ) }{\sigma _{1}} \bigl( \sigma _{1}C^{I}I-\pi _{1}C^{I} \bigr) + \frac{\mu _{2}\mathcal{P} ( \psi +\omega ) }{\sigma _{2}\varphi \psi \mathcal{H}_{4}\mathcal{H}_{5}} \biggl( 1- \frac{C_{6}^{Y}}{C^{Y}} \biggr)\bigl( \sigma _{2}C^{Y}Y-\pi _{2}C^{Y} \bigr) \\ &\quad{} +\vartheta _{1}\lambda \mathcal{H}_{3} ( 1-\beta ) S_{6}V_{6} \\ &\quad {}\times\int _{0}^{\kappa _{1}}\bar{\mathcal{H}}_{1}(\boldsymbol{\ell }) \biggl[ \frac{SV}{S_{6}V_{6}}- \frac{S(t-\boldsymbol{\ell })V(t-\boldsymbol{\ell })}{S_{6}V_{6}} +\ln \biggl( \frac{S(t-\boldsymbol{\ell })V(t-\boldsymbol{\ell })}{SV} \biggr) \biggr] \,d \boldsymbol{ \ell } \\ &\quad {}+\vartheta _{2}\lambda \mathcal{H}_{3} ( 1-\beta ) S_{6}I_{6} \\ &\quad {}\times\int _{0}^{\kappa _{1}}\bar{\mathcal{H}}_{1}( \boldsymbol{\ell }) \biggl[ \frac{SI}{S_{6}I_{6}}- \frac{S(t-\boldsymbol{\ell })I(t-\boldsymbol{\ell })}{S_{6}I_{6}} +\ln \biggl( \frac{S(t-\boldsymbol{\ell })I(t-\boldsymbol{\ell })}{SI} \biggr) \biggr] \,d \boldsymbol{ \ell } \\ &\quad {}+\vartheta _{1}\beta ( \gamma +\lambda ) S_{6}V_{6} \\ &\quad {}\times\int _{0}^{\kappa _{2}}\bar{\mathcal{H}}_{2}( \boldsymbol{\ell }) \biggl[ \frac{SV}{S_{6}V_{6}}- \frac{S(t-\boldsymbol{\ell })V(t-\boldsymbol{\ell })}{S_{6}V_{6}} +\ln \biggl( \frac{S(t-\boldsymbol{\ell })V(t-\boldsymbol{\ell })}{SV} \biggr) \biggr] \,d \boldsymbol{ \ell } \\ &\quad {}+\vartheta _{2}\beta ( \gamma +\lambda ) S_{6}I_{6} \\ &\quad {}\times\int _{0}^{\kappa _{2}}\bar{\mathcal{H}}_{2}( \boldsymbol{\ell }) \biggl[ \frac{SI}{S_{6}I_{6}}- \frac{S(t-\boldsymbol{\ell })I(t-\boldsymbol{\ell })}{S_{6}I_{6}} +\ln \biggl( \frac{S(t-\boldsymbol{\ell })I(t-\boldsymbol{\ell })}{SI} \biggr) \biggr] \,d \boldsymbol{ \ell } \\ &\quad {}+\lambda ( \gamma +\lambda ) L_{6}\int _{0}^{\kappa _{3}}\bar{\mathcal{H}}_{3}( \boldsymbol{\ell }) \biggl[ \frac{L}{L_{6}}-\frac{L(t-\boldsymbol{\ell })}{L_{6}}+\ln \biggl( \frac{L(t-\boldsymbol{\ell })}{L} \biggr) \biggr] \,d\boldsymbol{\ell } \\ &\quad{}+\frac{\mathcal{P}\vartheta _{3}S_{6}Y_{6}}{\mathcal{H}_{4}} \int _{0}^{\kappa _{4}}\bar{\mathcal{H}}_{4}( \boldsymbol{\ell }) \biggl[ \frac{SY}{S_{6}Y_{6}}- \frac{S(t-\boldsymbol{\ell })Y(t-\boldsymbol{\ell })}{S_{6}Y_{6}}+ \ln \biggl( \frac{S(t-\boldsymbol{\ell })Y(t-\boldsymbol{\ell })}{SY} \biggr) \biggr] \,d\boldsymbol{\ell } \\ &\quad{}+ \frac{\mathcal{P} ( \psi +\omega ) E_{6}}{\varphi \mathcal{H}_{4}\mathcal{H}_{5}} \int _{0}^{\kappa _{5}} \bar{\mathcal{H}}_{5}( \boldsymbol{\ell }) \biggl[ \frac{E}{E_{6}}- \frac{E(t-\boldsymbol{\ell })}{E_{6}}+\ln \biggl( \frac{E(t-\boldsymbol{\ell })}{E} \biggr) \biggr] \,d \boldsymbol{\ell } \\ &\quad{}+\frac{b\mathcal{P}\vartheta _{1}S_{6}I_{6}}{\varepsilon } \int _{0}^{\kappa _{6}}\bar{\mathcal{H}}_{6}(\boldsymbol{\ell }) \biggl[ \frac{I}{I_{6}}- \frac{I(t-\boldsymbol{\ell })}{I_{6}}+\ln \biggl( \frac{I(t-\boldsymbol{\ell })}{I} \biggr) \biggr] \,d\boldsymbol{ \ell }. \end{aligned}$$

Collecting the terms of Eq. (25), we obtain

$$\begin{aligned} \frac{d\Phi _{6}}{dt} & =\mathcal{P} \biggl( 1-\frac{S_{6}}{S} \biggr) ( \eta -\varrho S ) +\mathcal{P}\vartheta _{2}S_{6}I+ \mathcal{P}\vartheta _{3}S_{6}Y-\vartheta _{1}\lambda \mathcal{H}_{3} ( 1- \beta ) \int _{0}^{\kappa _{1}}\bar{\mathcal{H}}_{1}( \boldsymbol{\ell }) \\ &\quad{}\times \frac{S(t-\boldsymbol{\ell })V(t-\boldsymbol{\ell })L_{6}}{L}\,d \boldsymbol{\ell }-\vartheta _{2} \lambda \mathcal{H}_{3} ( 1-\beta ) \int _{0}^{\kappa _{1}}\bar{\mathcal{H}}_{1}( \boldsymbol{\ell })\frac{S(t-\boldsymbol{\ell })I(t-\boldsymbol{\ell })L_{6}}{L}\,d \boldsymbol{\ell } \\ &\quad{}+\lambda \mathcal{H}_{3} ( \lambda +\gamma ) L_{6}-a ( \lambda +\gamma ) I-\vartheta _{1}\beta ( \gamma +\lambda ) \int _{0}^{\kappa _{2}} \bar{\mathcal{H}}_{2}( \boldsymbol{\ell }) \frac{S(t-\boldsymbol{\ell })V(t-\boldsymbol{\ell })I_{6}}{I}\,d\boldsymbol{\ell } \\ &\quad{}-\vartheta _{2}\beta ( \gamma +\lambda ) \int _{0}^{\kappa _{2}}\bar{\mathcal{H}}_{2}(\boldsymbol{\ell }) \frac{S(t-\boldsymbol{\ell })I(t-\boldsymbol{\ell })I_{6}}{I}\,d\boldsymbol{\ell }\\ &\quad {}- \lambda ( \lambda +\gamma ) \int _{0}^{\kappa _{3}} \bar{\mathcal{H}}_{3}( \boldsymbol{\ell })\frac{L(t-\boldsymbol{\ell })I_{6}}{I}\,d\boldsymbol{\ell }+a ( \lambda +\gamma ) I_{6}+\mu _{1} ( \lambda + \gamma ) C^{I}I_{6} \\ &\quad{}+ \frac{\mathcal{P}r}{\varphi \mathcal{H}_{4}}Y- \frac{\mathcal{P}\vartheta _{3}}{\mathcal{H}_{4}} \int _{0}^{ \kappa _{4}}\bar{\mathcal{H}}_{4}( \boldsymbol{\ell }) \frac{S(t-\boldsymbol{\ell })Y(t-\boldsymbol{\ell })E_{6}}{E}\,d\boldsymbol{\ell } \\ &\quad{}-\frac{\mathcal{P}r}{\varphi \mathcal{H}_{4}}\frac{YE_{6}}{E}+ \frac{\mathcal{P} ( \psi +\omega ) }{\varphi \mathcal{H}_{4}}E_{6}- \frac{\mathcal{P}\delta ( \psi +\omega ) }{\varphi \psi \mathcal{H}_{4}\mathcal{H}_{5}}Y\\ &\quad {}- \frac{\mathcal{P} ( \psi +\omega ) }{\varphi \mathcal{H}_{4}\mathcal{H}_{5}} \int _{0}^{\kappa _{5}}\bar{\mathcal{H}}_{5}( \boldsymbol{\ell }) \frac{E(t-\boldsymbol{\ell })Y_{6}}{Y}\,d\boldsymbol{\ell }+ \frac{\mathcal{P}\delta ( \psi +\omega ) }{\varphi \psi \mathcal{H}_{4}\mathcal{H}_{5}}Y_{6} + \frac{\mu _{2}\mathcal{P} ( \psi +\omega ) }{\varphi \psi \mathcal{H}_{4}\mathcal{H}_{5}}C^{Y}Y_{6}\\ &\quad{}- \frac{b\mathcal{P}\vartheta _{1}S_{6}}{\varepsilon } \int _{0}^{\kappa _{6}}\bar{\mathcal{H}}_{6}(\boldsymbol{\ell }) \frac{I(t-\boldsymbol{\ell })V_{6}}{V}\,d\boldsymbol{\ell }+\mathcal{P}\vartheta _{1}S_{6}V_{6}- \frac{\mu _{1}\pi _{1} ( \lambda +\gamma ) }{\sigma _{1}}C^{I} \\ &\quad{}- \frac{\mu _{2}\pi _{2}\mathcal{P} ( \psi +\omega ) }{\sigma _{2}\varphi \psi \mathcal{H}_{4}\mathcal{H}_{5}}C^{Y}- \frac{\mu _{2}\mathcal{P} ( \psi +\omega ) }{\varphi \psi \mathcal{H}_{4}\mathcal{H}_{5}}C_{6}^{Y}Y+ \frac{\mu _{2}\pi _{2}\mathcal{P} ( \psi +\omega ) }{\sigma _{2}\varphi \psi \mathcal{H}_{4}\mathcal{H}_{5}}C_{6}^{Y} \\ &\quad{}+\lambda \mathcal{H}_{3} ( 1-\beta ) \vartheta _{1}S_{6}V_{6}\int _{0}^{\kappa _{1}}\bar{\mathcal{H}}_{1}( \boldsymbol{\ell }) \ln \biggl( \frac{S(t-\boldsymbol{\ell })V(t-\boldsymbol{\ell })}{SV} \biggr) \,d \boldsymbol{\ell }\\ &\quad {}+\lambda \mathcal{H}_{3} ( 1-\beta ) \vartheta _{2}S_{6}I_{6}\int _{0}^{\kappa _{1}}\bar{\mathcal{H}}_{1}( \boldsymbol{\ell })\ln \biggl( \frac{S(t-\boldsymbol{\ell })I(t-\boldsymbol{\ell })}{SI} \biggr) \,d\boldsymbol{\ell } \\ &\quad{} +\beta ( \gamma +\lambda ) \vartheta _{1}S_{6}V_{6}\int _{0}^{\kappa _{2}}\bar{\mathcal{H}}_{2}( \boldsymbol{\ell }) \ln \biggl( \frac{S(t-\boldsymbol{\ell })V(t-\boldsymbol{\ell })}{SV} \biggr) \,d \boldsymbol{\ell } \\ &\quad{}+\beta ( \gamma +\lambda ) \vartheta _{2}S_{6}I_{6} \int _{0}^{\kappa _{2}}\bar{\mathcal{H}}_{2}( \boldsymbol{\ell }) \ln \biggl( \frac{S(t-\boldsymbol{\ell })I(t-\boldsymbol{\ell })}{SI} \biggr) \,d \boldsymbol{\ell }\\ &\quad {}+\lambda ( \gamma +\lambda ) L_{6} \int _{0}^{ \kappa _{3}}\bar{\mathcal{H}}_{3}( \boldsymbol{\ell }) \ln \biggl( \frac{L(t-\boldsymbol{\ell })}{L} \biggr) \,d \boldsymbol{\ell }\\ &\quad {}+\frac{\mathcal{P}\vartheta _{3}S_{6}Y_{6}}{\mathcal{H}_{4}} \int _{0}^{\kappa _{4}}\bar{\mathcal{H}}_{4}(\boldsymbol{\ell })\ln \biggl( \frac{S(t-\boldsymbol{\ell })Y(t-\boldsymbol{\ell })}{SY} \biggr) \,d \boldsymbol{\ell }\\ &\quad {}+ \frac{\mathcal{P} ( \psi +\omega ) E_{6}}{\varphi \mathcal{H}_{4}\mathcal{H}_{5}} \int _{0}^{\kappa _{5}}\bar{\mathcal{H}}_{5}( \boldsymbol{\ell })\ln \biggl( \frac{E(t-\boldsymbol{\ell })}{E} \biggr) \,d \boldsymbol{\ell }\\ &\quad {}+ \frac{b\mathcal{P}\vartheta _{1}S_{6}\mathcal{H}_{6}}{\varepsilon }I+ \frac{b\mathcal{P}\vartheta _{1}S_{6}I_{6}}{\varepsilon } \int _{0}^{\kappa _{6}}\bar{\mathcal{H}}_{6}( \boldsymbol{\ell })\ln \biggl( \frac{I(t-\boldsymbol{\ell })}{I} \biggr) \,d\boldsymbol{\ell }. \end{aligned}$$

Using the steady state conditions for

$$\begin{aligned} & \eta =\varrho S_{6}+\vartheta _{1}S_{6}V_{6}+ \vartheta _{2}S_{6}I_{6}+\vartheta _{3}S_{6}Y_{6}, \qquad \mathcal{H}_{1} ( 1-\beta ) ( \vartheta _{1}S_{6}V_{6}+\vartheta _{2}S_{6}I_{6} ) = ( \lambda +\gamma ) L_{6}, \\ & \beta \mathcal{H}_{2} ( \vartheta _{1}S_{6}V_{6}+ \vartheta _{2}S_{6}I_{6} ) +\lambda \mathcal{H}_{3}L_{6}=aI_{6}, \qquad Y_{6}=\frac{\pi _{2}}{\sigma _{2}}, \qquad V_{6}= \frac{b\mathcal{H}_{6}I_{6}}{\varepsilon }, \\ & \vartheta _{3}S_{6}Y_{6}+ \frac{rY_{6}}{\varphi \mathcal{H}_{4}}=\frac{ ( \psi +\omega ) E_{6}}{\varphi \mathcal{H}_{4}}= \frac{\delta ( \psi +\omega ) }{\varphi \psi \mathcal{H}_{4}\mathcal{H}_{5}}Y_{6}+ \frac{\mu _{2} ( \psi +\omega ) }{\varphi \psi \mathcal{H}_{4}\mathcal{H}_{5}}C_{6}^{Y}Y_{6}, \end{aligned}$$

we get

$$ \mathcal{P} ( \vartheta _{1}S_{6}V_{6}+ \vartheta _{2}S_{6}I_{6} ) =a ( \lambda +\gamma ) I_{6}. $$

Moreover, we get

$$\begin{aligned} \frac{d\Phi _{6}}{dt} & =\mathcal{P} \biggl( 1-\frac{S_{6}}{S} \biggr) ( \varrho S_{6}-\varrho S ) +\mathcal{P} ( \vartheta _{1}S_{6}V_{6}+ \vartheta _{2}S_{6}I_{6}+\vartheta _{3}S_{6}Y_{6} ) \biggl( 1-\frac{S_{6}}{S} \biggr) \\ &\quad{}-\lambda \mathcal{H}_{3} ( 1-\beta ) \vartheta _{1}S_{6}V_{6}\int _{0}^{\kappa _{1}}\bar{\mathcal{H}}_{1}( \boldsymbol{\ell }) \frac{S(t-\boldsymbol{\ell })V(t-\boldsymbol{\ell })L_{6}}{S_{6}V_{6}L}\,d \boldsymbol{\ell }-\lambda \mathcal{H}_{3} ( 1-\beta ) \vartheta _{2}S_{6}I_{6} \\ &\quad{}\times \int _{0}^{\kappa _{1}}\bar{\mathcal{H}}_{1}( \boldsymbol{\ell })\frac{S(t-\boldsymbol{\ell })I(t-\boldsymbol{\ell })L_{6}}{S_{6}I_{6}L}\,d \boldsymbol{\ell }+\lambda \mathcal{H}_{1}\mathcal{H}_{3} ( 1-\beta ) ( \vartheta _{1}S_{6}V_{6}+\vartheta _{2}S_{6}I_{6} ) \\ &\quad{}-\beta ( \gamma +\lambda ) \vartheta _{1}S_{6}V_{6} \int _{0}^{\kappa _{2}}\bar{\mathcal{H}}_{2}( \boldsymbol{\ell }) \frac{S(t-\boldsymbol{\ell })V(t-\boldsymbol{\ell })I_{6}}{S_{6}V_{6}I}\,d \boldsymbol{\ell }\\ &\quad {}-\beta ( \gamma +\lambda ) \vartheta _{2}S_{6}I_{6} \int _{0}^{\kappa _{2}}\bar{\mathcal{H}}_{2}( \boldsymbol{\ell }) \frac{S(t-\boldsymbol{\ell })I(t-\boldsymbol{\ell })}{S_{6}I}\,d \boldsymbol{\ell }\\ &\quad {}-\lambda \mathcal{H}_{1} ( 1-\beta ) ( \vartheta _{1}S_{6}V_{6}+ \vartheta _{2}S_{6}I_{6} ) \int _{0}^{ \kappa _{3}}\bar{\mathcal{H}}_{3}( \boldsymbol{\ell }) \frac{L(t-\boldsymbol{\ell })I_{6}}{L_{6}I}\,d\boldsymbol{\ell } \\ &\quad{}+\mathcal{P} ( \vartheta _{1}S_{6}V_{6}+ \vartheta _{2}S_{6}I_{6} ) -\frac{\mathcal{P}\vartheta _{3}S_{6}Y_{6}}{\mathcal{H}_{4}} \int _{0}^{\kappa _{4}}\bar{\mathcal{H}}_{4}(\boldsymbol{\ell }) \frac{S(t-\boldsymbol{\ell })Y(t-\boldsymbol{\ell })E_{6}}{S_{6}Y_{6}E}\,d \boldsymbol{\ell }\\ &\quad {}-\frac{\mathcal{P}rY_{6}}{\varphi \mathcal{H}_{4}}\frac{YE_{6}}{Y_{6}E}+\mathcal{P}\vartheta _{3}S_{6}Y_{6}+ \frac{\mathcal{P}rY_{6}}{\varphi \mathcal{H}_{4}} \\ &\quad{}- \biggl( \frac{\mathcal{P}\vartheta _{3}S_{6}Y_{6}}{\mathcal{H}_{5}}+ \frac{\mathcal{P}rY_{6}}{\varphi \mathcal{H}_{4}\mathcal{H}_{5}} \biggr) \int _{0}^{\kappa _{5}} \bar{\mathcal{H}}_{5}( \boldsymbol{\ell })\frac{E(t-\boldsymbol{\ell })Y_{6}}{E_{6}Y}\,d \boldsymbol{\ell } \\ &\quad{}+\mathcal{P}\vartheta _{3}S_{6}Y_{6}+ \frac{\mathcal{P}rY_{6}}{\varphi \mathcal{H}_{4}}- \frac{\mathcal{P}\vartheta _{1}S_{6}V_{6}}{\mathcal{H}_{6}} \int _{0}^{\kappa _{6}}\bar{\mathcal{H}}_{6}( \boldsymbol{\ell })\frac{I(t-\boldsymbol{\ell })V_{6}}{I_{6}V}\,d\boldsymbol{\ell }+\mathcal{P} \vartheta _{1}S_{6}V_{6} \\ &\quad{}+\lambda \mathcal{H}_{3} ( 1-\beta ) \vartheta _{1}S_{6}V_{6}\int _{0}^{\kappa _{1}}\bar{\mathcal{H}}_{1}( \boldsymbol{\ell }) \ln \biggl( \frac{S(t-\boldsymbol{\ell })V(t-\boldsymbol{\ell })}{SV} \biggr) \,d \boldsymbol{\ell }\\ &\quad {}+\lambda \mathcal{H}_{3} ( 1-\beta ) \vartheta _{2}S_{6}I_{6} \int _{0}^{\kappa _{1}}\bar{\mathcal{H}}_{1}( \boldsymbol{\ell })\ln \biggl( \frac{S(t-\boldsymbol{\ell })I(t-\boldsymbol{\ell })}{SI} \biggr) \,d \boldsymbol{\ell }\\ &\quad {}+\beta ( \gamma +\lambda ) \vartheta _{1}S_{6}V_{6}\int _{0}^{\kappa _{2}}\bar{\mathcal{H}}_{2}( \boldsymbol{\ell }) \ln \biggl( \frac{S(t-\boldsymbol{\ell })V(t-\boldsymbol{\ell })}{SV} \biggr) \,d\boldsymbol{\ell }\\ &\quad {}+\beta ( \gamma +\lambda ) \vartheta _{2}S_{6}I_{6}\int _{0}^{\kappa _{2}}\bar{\mathcal{H}}_{2}( \boldsymbol{\ell }) \ln \biggl( \frac{S(t-\boldsymbol{\ell })I(t-\boldsymbol{\ell })}{SI} \biggr) \,d \boldsymbol{\ell } \\ &\quad{}+\lambda \mathcal{H}_{1} ( 1-\beta ) ( \vartheta _{1}S_{6}V_{6}+\vartheta _{2}S_{6}I_{6} ) \int _{0}^{ \kappa _{3}}\bar{\mathcal{H}}_{3}( \boldsymbol{\ell })\ln \biggl( \frac{L(t-\boldsymbol{\ell })}{L} \biggr) \,d\boldsymbol{\ell }\\ &\quad {}+ \frac{\mathcal{P}\vartheta _{3}S_{6}Y_{6}}{\mathcal{H}_{4}} \int _{0}^{\kappa _{4}}\bar{\mathcal{H}}_{4}( \boldsymbol{\ell }) \ln \biggl( \frac{S(t-\boldsymbol{\ell })Y(t-\boldsymbol{\ell })}{SY} \biggr) \,d\boldsymbol{\ell }\\ &\quad {}+ \biggl( \frac{\mathcal{P}\vartheta _{3}S_{6}Y_{6}}{\mathcal{H}_{5}}+ \frac{\mathcal{P}rY_{6}}{\varphi \mathcal{H}_{4}\mathcal{H}_{5}} \biggr) \int _{0}^{\kappa _{5}} \bar{\mathcal{H}}_{5}( \boldsymbol{\ell })\ln \biggl( \frac{E(t-\boldsymbol{\ell })}{E} \biggr) \,d \boldsymbol{\ell } \\ &\quad{}+\frac{\mathcal{P}\vartheta _{1}S_{6}V_{6}}{\mathcal{H}_{6}} \int _{0}^{\kappa _{6}}\bar{\mathcal{H}}_{6}( \boldsymbol{\ell })\ln \biggl( \frac{I(t-\boldsymbol{\ell })}{I} \biggr) \,d\boldsymbol{\ell }+\mu _{1} ( \lambda +\gamma ) \biggl( I_{6}- \frac{\pi _{1}}{\sigma _{1}} \biggr) C^{I}. \end{aligned}$$

Using the equalities given by (10) and (11) in case of $n=m=6$, we get

26 $$\begin{aligned} \frac{d\Phi _{6}}{dt} & =-\varrho \mathcal{P} \frac{(S-S_{6})^{2}}{S}-\mathcal{P} ( \vartheta _{1}S_{6}V_{6}+\vartheta _{2}S_{6}I_{6}+\vartheta _{3}S_{6}Y_{6} ) \biggl[ \frac{S_{6}}{S}-1- \ln \biggl( \frac{S_{6}}{S} \biggr) \biggr] \\ &\quad{}-\lambda \mathcal{H}_{3} ( 1-\beta ) \vartheta _{1}S_{6}V_{6} \\ &\quad {}\times\int _{0}^{\kappa _{1}}\bar{\mathcal{H}}_{1}( \boldsymbol{\ell }) \biggl[ \frac{S(t-\boldsymbol{\ell })V(t-\boldsymbol{\ell })L_{6}}{S_{6}V_{6}L}-1- \ln \biggl( \frac{S(t-\boldsymbol{\ell })V(t-\boldsymbol{\ell })L_{6}}{S_{6}V_{6}L} \biggr) \biggr] \,d\boldsymbol{\ell } \\ &\quad{}-\lambda \mathcal{H}_{3} ( 1-\beta ) \vartheta _{2}S_{6}I_{6} \\ &\quad {}\times\int _{0}^{\kappa _{1}}\bar{\mathcal{H}}_{1}( \boldsymbol{\ell }) \biggl[ \frac{S(t-\boldsymbol{\ell })I(t-\boldsymbol{\ell })L_{6}}{S_{6}I_{6}L}-1- \ln \biggl( \frac{S(t-\boldsymbol{\ell })I(t-\boldsymbol{\ell })L_{6}}{S_{6}I_{6}L} \biggr) \biggr] \,d\boldsymbol{\ell } \\ &\quad{}-\beta ( \gamma +\lambda ) \vartheta _{1}S_{6}V_{6} \\ &\quad {}\times\int _{0}^{\kappa _{2}}\bar{\mathcal{H}}_{2}( \boldsymbol{\ell }) \biggl[ \frac{S(t-\boldsymbol{\ell })V(t-\boldsymbol{\ell })I_{6}}{S_{6}V_{6}I}-1- \ln \biggl( \frac{S(t-\boldsymbol{\ell })V(t-\boldsymbol{\ell })I_{6}}{S_{6}V_{6}I} \biggr) \biggr] \,d\boldsymbol{\ell } \\ &\quad{}-\beta ( \gamma +\lambda ) \vartheta _{2}S_{6}I_{6} \int _{0}^{\kappa _{2}}\bar{\mathcal{H}}_{2}( \boldsymbol{\ell }) \biggl[ \frac{S(t-\boldsymbol{\ell })I(t-\boldsymbol{\ell })}{S_{6}I}-1-\ln \biggl( \frac{S(t-\boldsymbol{\ell })I(t-\boldsymbol{\ell })}{S_{6}I} \biggr) \biggr] \,d\boldsymbol{\ell } \\ &\quad{}-\lambda \mathcal{H}_{1} ( 1-\beta ) ( \vartheta _{1}S_{6}V_{6}+\vartheta _{2}S_{6}I_{6} ) \\ &\quad {}\times\int _{0}^{ \kappa _{3}}\bar{\mathcal{H}}_{3}( \boldsymbol{\ell })) \biggl[ \frac{L(t-\boldsymbol{\ell })I_{6}}{L_{6}I}-1-\ln \biggl( \frac{L(t-\boldsymbol{\ell })I_{6}}{L_{6}I} \biggr) \biggr] \,d\boldsymbol{\ell } \\ &\quad{}-\frac{\mathcal{P}\vartheta _{3}S_{6}Y_{6}}{\mathcal{H}_{4}} \int _{0}^{\kappa _{4}}\bar{\mathcal{H}}_{4}( \boldsymbol{\ell }) \biggl[ \frac{S(t-\boldsymbol{\ell })Y(t-\boldsymbol{\ell })E_{6}}{S_{6}Y_{6}E}-1-\ln \biggl( \frac{S(t-\boldsymbol{\ell })Y(t-\boldsymbol{\ell })E_{6}}{S_{6}Y_{6}E} \biggr) \biggr] \,d\boldsymbol{\ell } \\ &\quad{}-\frac{\mathcal{P}rY_{6}}{\varphi \mathcal{H}_{4}} \biggl[ \frac{YE_{6}}{Y_{6}E}-1-\ln \biggl( \frac{YE_{6}}{Y_{6}E} \biggr) \biggr] \\ &\quad{}- \biggl( \frac{\mathcal{P}\vartheta _{3}S_{6}Y_{6}}{\mathcal{H}_{5}}+\frac{\mathcal{P}rY_{6}}{\varphi \mathcal{H}_{4}\mathcal{H}_{5}} \biggr) \int _{0}^{\kappa _{5}}\bar{\mathcal{H}}_{5}( \boldsymbol{\ell }) \biggl[ \frac{E(t-\boldsymbol{\ell })Y_{6}}{E_{6}Y}-1-\ln \biggl( \frac{E(t-\boldsymbol{\ell })Y_{6}}{E_{6}Y} \biggr) \biggr] \,d \boldsymbol{\ell } \\ &\quad{}-\frac{\mathcal{P}\vartheta _{1}S_{6}V_{6}}{\mathcal{H}_{6}} \int _{0}^{\kappa _{6}}\bar{\mathcal{H}}_{6}( \boldsymbol{\ell }) \biggl[ \frac{I(t-\boldsymbol{\ell })V_{6}}{I_{6}V}-1-\ln \biggl( \frac{I(t-\boldsymbol{\ell })V_{6}}{I_{6}V} \biggr) \biggr] \,d \boldsymbol{\ell } \\ &\quad {}+\mu _{1} ( \lambda +\gamma ) \biggl( I_{6}- \frac{\pi _{1}}{\sigma _{1}} \biggr) C^{I}. \end{aligned}$$

Therefore, Eq. (26) becomes

$$\begin{aligned} \frac{d\Phi _{6}}{dt} & =-\varrho \mathcal{P} \frac{(S-S_{6})^{2}}{S}-\mathcal{P} ( \vartheta _{1}S_{6}V_{6}+\vartheta _{2}S_{6}I_{6}+\vartheta _{3}S_{6}Y_{6} ) +\digamma \biggl( \frac{S_{6}}{S} \biggr) \\ &\quad{}-\lambda \mathcal{H}_{3} ( 1-\beta ) \vartheta _{1}S_{6}V_{6}\int _{0}^{\kappa _{1}}\bar{\mathcal{H}}_{1}( \boldsymbol{\ell }) \digamma \biggl( \frac{S(t-\boldsymbol{\ell })V(t-\boldsymbol{\ell })L_{6}}{S_{6}V_{6}L} \biggr) \,d \boldsymbol{ \ell } \\ &\quad{}-\lambda \mathcal{H}_{3} ( 1-\beta ) \vartheta _{2}S_{6}I_{6}\int _{0}^{\kappa _{1}}\bar{\mathcal{H}}_{1}( \boldsymbol{\ell }) \digamma \biggl( \frac{S(t-\boldsymbol{\ell })I(t-\boldsymbol{\ell })L_{6}}{S_{6}I_{6}L} \biggr) \,d \boldsymbol{ \ell } \\ &\quad{}-\beta ( \gamma +\lambda ) \vartheta _{1}S_{6}V_{6} \int _{0}^{\kappa _{2}}\bar{\mathcal{H}}_{2}( \boldsymbol{\ell }) \digamma \biggl( \frac{S(t-\boldsymbol{\ell })V(t-\boldsymbol{\ell })I_{6}}{S_{6}V_{6}I} \biggr) \,d \boldsymbol{ \ell } \\ &\quad{}-\beta ( \gamma +\lambda ) \vartheta _{2}S_{6}I_{6} \int _{0}^{\kappa _{2}}\bar{\mathcal{H}}_{2}( \boldsymbol{\ell }) \digamma \biggl( \frac{S(t-\boldsymbol{\ell })I(t-\boldsymbol{\ell })}{S_{6}I} \biggr) \,d\boldsymbol{\ell } \\ &\quad{}-\lambda \mathcal{H}_{1} ( 1-\beta ) ( \vartheta _{1}S_{6}V_{6}+\vartheta _{2}S_{6}I_{6} ) \int _{0}^{ \kappa _{3}}\bar{\mathcal{H}}_{3}( \boldsymbol{\ell }))\digamma \biggl( \frac{L(t-\boldsymbol{\ell })I_{6}}{L_{6}I} \biggr) \,d\boldsymbol{\ell } \\ &\quad{}-\frac{\mathcal{P}\vartheta _{3}S_{6}Y_{6}}{\mathcal{H}_{4}} \int _{0}^{\kappa _{4}}\bar{\mathcal{H}}_{4}( \boldsymbol{\ell }) \digamma \biggl( \frac{S(t-\boldsymbol{\ell })Y(t-\boldsymbol{\ell })E_{6}}{S_{6}Y_{6}E} \biggr) \,d \boldsymbol{ \ell }-\frac{\mathcal{P}rY_{6}}{\varphi \mathcal{H}_{4}} \digamma \biggl( \frac{YE_{6}}{Y_{6}E} \biggr) \\ &\quad{}- \biggl( \frac{\mathcal{P}\vartheta _{3}S_{6}Y_{6}}{\mathcal{H}_{5}}+\frac{\mathcal{P}rY_{6}}{\varphi \mathcal{H}_{4}\mathcal{H}_{5}} \biggr) \int _{0}^{\kappa _{5}}\bar{\mathcal{H}}_{5}( \boldsymbol{\ell })\digamma \biggl( \frac{E(t-\boldsymbol{\ell })Y_{6}}{E_{6}Y} \biggr) \,d\boldsymbol{\ell } \\ &\quad{}-\frac{\mathcal{P}\vartheta _{1}S_{6}V_{6}}{\mathcal{H}_{6}} \int _{0}^{\kappa _{6}}\bar{\mathcal{H}}_{6}( \boldsymbol{\ell }) \digamma \biggl( \frac{I(t-\boldsymbol{\ell })V_{6}}{I_{6}V} \biggr) \,d \boldsymbol{ \ell } \\ &\quad{}+ \frac{\mu _{1} ( \gamma +\lambda ) [ \pi _{1}\sigma _{2} ( b\vartheta _{1}\mathcal{H}_{6}+\varepsilon \vartheta _{2} ) +\varepsilon \sigma _{1} ( \pi _{2}\vartheta _{3}+\varrho \sigma _{2} ) ] }{\sigma _{1}\sigma _{2} ( b\vartheta _{1}\mathcal{H}_{6}+\varepsilon \vartheta _{2} ) } ( \Re _{7}-1 ) C^{I}. \end{aligned}$$

Hence, if $\Re _{7}\leq 1$, then $\frac{d\Phi _{6}}{dt}\leq 0$ for all $S,L,I,E,Y,V,C^{I}>0$. Similar to the previous theorems, one can show that $\frac{d\Phi _{6}}{dt}=0$ at $(S,L,I,E,Y,V,C^{I})=(S_{6},L_{6},I_{6},E_{6},Y_{6},V_{6},0)$. The solutions of system (5) reach $\Upsilon _{6}^{{\prime }}$ which contains elements with $E(t)=E_{6}$, $Y(t)=Y_{6}$, and then $\frac{dY(t)}{dt}=0$. The fifth equation of system (5) becomes

$$ 0=\frac{dY(t)}{dt}=\psi \mathcal{H}_{5}E_{6}-\delta Y_{6}-\mu _{2}C^{Y}(t)Y_{6}, $$

which yields $C^{Y}(t)=C_{6}^{Y}$ for all *t*, and hence . Applying LLAS theorem, we get  is GAS. □

### Theorem 8

*If* $\Re _{7}>1$ *and* $\Re _{8}>1$, *then*  *is GAS*.

### Proof

Consider

$$\begin{aligned} \Phi _{7} & =\mathcal{P}S_{7}\digamma \biggl( \frac{S}{S_{7}} \biggr) + \lambda \mathcal{H}_{3}L_{7} \digamma \biggl( \frac{L}{L_{7}} \biggr) + ( \gamma +\lambda ) I_{7}\digamma \biggl( \frac{I}{I_{7}} \biggr) + \frac{\mathcal{P}}{\varphi \mathcal{H}_{4}}E_{7} \digamma \biggl( \frac{E}{E_{7}} \biggr) \\ &\quad{}+ \frac{\mathcal{P} ( \psi +\omega ) }{\varphi \psi \mathcal{H}_{4}\mathcal{H}_{5}}Y_{7}\digamma \biggl( \frac{Y}{Y_{7}} \biggr) + \frac{\mathcal{P}\vartheta _{1}S_{7}}{\varepsilon }V_{7}\digamma \biggl( \frac{V}{V_{7}} \biggr) + \frac{\mu _{1} ( \gamma +\lambda ) }{\sigma _{1}}C_{7}^{I} \digamma \biggl( \frac{C^{I}}{C_{7}^{I}} \biggr) \\ &\quad{}+ \frac{\mu _{2}\mathcal{P} ( \psi +\omega ) }{\sigma _{2}\varphi \psi \mathcal{H}_{4}\mathcal{H}_{5}}C_{7}^{Y}\digamma \biggl( \frac{C^{Y}}{C_{7}^{Y}} \biggr)\\ &\quad {} +\vartheta _{1}\lambda \mathcal{H}_{3} ( 1-\beta ) S_{7}V_{7} \int _{0}^{\kappa _{1}} \bar{\mathcal{H}}_{1}( \boldsymbol{\ell }) \int _{t-\boldsymbol{\ell }}^{t}\digamma \biggl( \frac{S(\varkappa )V(\varkappa )}{S_{7}V_{7}} \biggr) \,d\varkappa \,d \boldsymbol{\ell } \\ &\quad{}+\vartheta _{2}\lambda \mathcal{H}_{3} ( 1-\beta ) S_{7}I_{7}\int _{0}^{\kappa _{1}}\bar{\mathcal{H}}_{1}( \boldsymbol{\ell }) \int _{t-\boldsymbol{\ell }}^{t}\digamma \biggl( \frac{S(\varkappa )I(\varkappa )}{S_{7}I_{7}} \biggr) \,d\varkappa \,d \boldsymbol{\ell }\\ &\quad {}+\vartheta _{1}\beta ( \gamma + \lambda ) S_{7}V_{7} \int _{0}^{\kappa _{2}}\bar{\mathcal{H}}_{2}( \boldsymbol{\ell }) \int _{t-\boldsymbol{\ell }}^{t}\digamma \biggl( \frac{S(\varkappa )V(\varkappa )}{S_{7}V_{7}} \biggr) \,d\varkappa \,d \boldsymbol{\ell }\\ &\quad {}+\vartheta _{2}\beta ( \gamma + \lambda ) S_{7}I_{7} \int _{0}^{\kappa _{2}}\bar{\mathcal{H}}_{2}( \boldsymbol{\ell }) \int _{t-\boldsymbol{\ell }}^{t}\digamma \biggl( \frac{S(\varkappa )I(\varkappa )}{S_{7}I_{7}} \biggr) \,d\varkappa \,d\boldsymbol{\ell } \\ &\quad{}+\lambda ( \gamma +\lambda ) L_{7} \int _{0}^{ \kappa _{3}}\bar{\mathcal{H}}_{3}( \boldsymbol{\ell }) \int _{t-\boldsymbol{\ell }}^{t}\digamma \biggl( \frac{L(\varkappa )}{L_{7}} \biggr) \,d\varkappa \,d \boldsymbol{\ell }\\ &\quad {}+ \frac{\mathcal{P}\vartheta _{3}S_{7}Y_{7}}{\mathcal{H}_{4}} \int _{0}^{\kappa _{4}}\bar{\mathcal{H}}_{4}( \boldsymbol{\ell }) \int _{t-\boldsymbol{\ell }}^{t}\digamma \biggl( \frac{S(\varkappa )Y(\varkappa )}{S_{7}Y_{7}} \biggr) \,d\varkappa \,d \boldsymbol{\ell } \\ &\quad{}+ \frac{\mathcal{P} ( \psi +\omega ) E_{7}}{\varphi \mathcal{H}_{4}\mathcal{H}_{5}} \int _{0}^{\kappa _{5}} \bar{\mathcal{H}}_{5}( \boldsymbol{\ell }) \int _{t-\boldsymbol{\ell }}^{t}\digamma \biggl( \frac{E(\varkappa )}{E_{7}} \biggr) \,d\varkappa \,d\boldsymbol{\ell }\\ &\quad {}+ \frac{b\mathcal{P}\vartheta _{1}S_{7}I_{7}}{\varepsilon } \int _{0}^{\kappa _{6}}\bar{\mathcal{H}}_{6}( \boldsymbol{\ell }) \int _{t-\boldsymbol{\ell }}^{t}\digamma \biggl( \frac{I(\varkappa )}{I_{7}} \biggr) \,d\varkappa \,d \boldsymbol{\ell }. \end{aligned}$$

Calculate $\frac{d\Phi _{7}}{dt}$ as follows:

27 $$\begin{aligned} \frac{d\Phi _{7}}{dt} & =\mathcal{P} \biggl( 1-\frac{S_{7}}{S} \biggr) ( \eta -\varrho S-\vartheta _{1}SV-\vartheta _{2}SI-\vartheta _{3}SY ) +\lambda \mathcal{H}_{3} \biggl( 1- \frac{L_{7}}{L} \biggr) \\ &\quad{}\times \biggl[ ( 1-\beta ) \int _{0}^{\kappa _{1}}\bar{\mathcal{H}}_{1}( \boldsymbol{\ell })S(t-\boldsymbol{\ell }) \bigl\{ \vartheta _{1}V(t- \boldsymbol{\ell })+\vartheta _{2}I(t-\boldsymbol{\ell }) \bigr\} \,d \boldsymbol{\ell }- ( \lambda +\gamma ) L \biggr] \\ &\quad {} + ( \gamma +\lambda ) \biggl( 1- \frac{I_{7}}{I} \biggr) \biggl[ \beta \int _{0}^{\kappa _{2}} \bar{\mathcal{H}}_{2}( \boldsymbol{\ell })S(t-\boldsymbol{\ell }) \bigl\{ \vartheta _{1}V(t- \boldsymbol{\ell })+\vartheta _{2}I(t-\boldsymbol{\ell }) \bigr\} \,d \boldsymbol{\ell } \\ &\quad{}+\lambda \int _{0}^{\kappa _{3}} \bar{\mathcal{H}}_{3}( \boldsymbol{\ell })L(t-\boldsymbol{\ell })\,d\boldsymbol{\ell }-aI-\mu _{1}C^{I}I \biggr] \\ &\quad{}+\frac{\mathcal{P}}{\varphi \mathcal{H}_{4}} \biggl( 1- \frac{E_{7}}{E} \biggr) \biggl[ \varphi \vartheta _{3} \int _{0}^{\kappa _{4}}\bar{\mathcal{H}}_{4}( \boldsymbol{\ell })S(t-\boldsymbol{\ell })Y(t- \boldsymbol{\ell })\,d\boldsymbol{ \ell }+rY- ( \psi +\omega ) E \biggr] \\ &\quad {}+ \frac{\mathcal{P} ( \psi +\omega ) }{\varphi \psi \mathcal{H}_{4}\mathcal{H}_{5}} \biggl( 1-\frac{Y_{7}}{Y} \biggr) \biggl[ \psi \int _{0}^{\kappa _{5}}\bar{\mathcal{H}}_{5}(\boldsymbol{\ell })E(t-\boldsymbol{\ell })\,d \boldsymbol{ \ell }-\delta Y-\mu _{2}C^{Y}Y \biggr] \\ &\quad {} + \frac{\mathcal{P}\vartheta _{1}S_{7}}{\varepsilon } \biggl( 1-\frac{V_{7}}{V} \biggr) \biggl[ b \int _{0}^{\kappa _{6}}\bar{\mathcal{H}}_{6}( \boldsymbol{\ell })I(t-\boldsymbol{\ell })\,d\boldsymbol{\ell }-\varepsilon V \biggr] \\ &\quad {}+ \frac{\mu _{1} ( \gamma +\lambda ) }{\sigma _{1}} \biggl( 1- \frac{C_{7}^{I}}{C^{I}} \biggr) \bigl( \sigma _{1}C^{I}I-\pi _{1}C^{I} \bigr) \\ &\quad {} + \frac{\mu _{2}\mathcal{P} ( \psi +\omega ) }{\sigma _{2}\varphi \psi \mathcal{H}_{4}\mathcal{H}_{5}} \biggl( 1-\frac{C_{7}^{Y}}{C^{Y}} \biggr) \bigl( \sigma _{2}C^{Y}Y-\pi _{2}C^{Y} \bigr) \\ &\quad {}+\vartheta _{1}\lambda \mathcal{H}_{3} ( 1-\beta ) S_{7}V_{7} \\ &\quad {}\times\int _{0}^{\kappa _{1}} \bar{\mathcal{H}}_{1}( \boldsymbol{\ell }) \biggl[ \frac{SV}{S_{7}V_{7}}- \frac{S(t-\boldsymbol{\ell })V(t-\boldsymbol{\ell })}{S_{7}V_{7}} +\ln \biggl( \frac{S(t-\boldsymbol{\ell })V(t-\boldsymbol{\ell })}{SV} \biggr) \biggr] \,d \boldsymbol{ \ell } \\ &\quad {}+\vartheta _{2}\lambda \mathcal{H}_{3} ( 1-\beta ) S_{7}I_{7} \\ &\quad {}\times\int _{0}^{\kappa _{1}}\bar{\mathcal{H}}_{1}( \boldsymbol{\ell }) \biggl[ \frac{SI}{S_{7}I_{7}}- \frac{S(t-\boldsymbol{\ell })I(t-\boldsymbol{\ell })}{S_{7}I_{7}} +\ln \biggl( \frac{S(t-\boldsymbol{\ell })I(t-\boldsymbol{\ell })}{SI} \biggr) \biggr] \,d \boldsymbol{ \ell } \\ &\quad {}+\vartheta _{1}\beta ( \gamma +\lambda ) S_{7}V_{7} \\ &\quad {}\times\int _{0}^{\kappa _{2}}\bar{\mathcal{H}}_{2}( \boldsymbol{\ell }) \biggl[ \frac{SV}{S_{7}V_{7}}- \frac{S(t-\boldsymbol{\ell })V(t-\boldsymbol{\ell })}{S_{7}V_{7}} +\ln \biggl( \frac{S(t-\boldsymbol{\ell })V(t-\boldsymbol{\ell })}{SV} \biggr) \biggr] \,d \boldsymbol{ \ell } \\ &\quad {}+\vartheta _{2}\beta ( \gamma +\lambda ) S_{7}I_{7} \\ &\quad {}\times\int _{0}^{\kappa _{2}}\bar{\mathcal{H}}_{2}( \boldsymbol{\ell }) \biggl[ \frac{SI}{S_{7}I_{7}}- \frac{S(t-\boldsymbol{\ell })I(t-\boldsymbol{\ell })}{S_{7}I_{7}} +\ln \biggl( \frac{S(t-\boldsymbol{\ell })I(t-\boldsymbol{\ell })}{SI} \biggr) \biggr] \,d \boldsymbol{ \ell } \\ &\quad {}+\lambda ( \gamma +\lambda ) L_{7}\int _{0}^{\kappa _{3}}\bar{\mathcal{H}}_{3}( \boldsymbol{\ell }) \biggl[ \frac{L}{L_{7}}-\frac{L(t-\boldsymbol{\ell })}{L_{7}}+\ln \biggl( \frac{L(t-\boldsymbol{\ell })}{L} \biggr) \biggr] \,d\boldsymbol{\ell } \\ &\quad{}+\frac{\mathcal{P}\vartheta _{3}S_{7}Y_{7}}{\mathcal{H}_{4}} \int _{0}^{\kappa _{4}}\bar{\mathcal{H}}_{4}( \boldsymbol{\ell }) \biggl[ \frac{SY}{S_{7}Y_{7}}- \frac{S(t-\boldsymbol{\ell })Y(t-\boldsymbol{\ell })}{S_{7}Y_{7}}+ \ln \biggl( \frac{S(t-\boldsymbol{\ell })Y(t-\boldsymbol{\ell })}{SY} \biggr) \biggr] \,d\boldsymbol{\ell } \\ &\quad{}+ \frac{\mathcal{P} ( \psi +\omega ) E_{7}}{\varphi \mathcal{H}_{4}\mathcal{H}_{5}} \int _{0}^{\kappa _{5}} \bar{\mathcal{H}}_{5}( \boldsymbol{\ell }) \biggl[ \frac{E}{E_{7}}- \frac{E(t-\boldsymbol{\ell })}{E_{7}}+\ln \biggl( \frac{E(t-\boldsymbol{\ell })}{E} \biggr) \biggr] \,d \boldsymbol{\ell } \\ &\quad{}+\frac{b\mathcal{P}\vartheta _{1}S_{7}I_{7}}{\varepsilon } \int _{0}^{\kappa _{6}}\bar{\mathcal{H}}_{6}(\boldsymbol{\ell }) \biggl[ \frac{I}{I_{7}}- \frac{I(t-\boldsymbol{\ell })}{I_{7}}+\ln \biggl( \frac{I(t-\boldsymbol{\ell })}{I} \biggr) \biggr] \,d\boldsymbol{ \ell }. \end{aligned}$$

Summing the terms of Eq. (27), we get

$$\begin{aligned} \frac{d\Phi _{7}}{dt} & =\mathcal{P} \biggl( 1-\frac{S_{7}}{S} \biggr) ( \eta -\varrho S ) +\mathcal{P}\vartheta _{2}S_{7}I+ \mathcal{P}\vartheta _{3}S_{7}Y-\vartheta _{1}\lambda \mathcal{H}_{3} ( 1- \beta ) \int _{0}^{\kappa _{1}}\bar{\mathcal{H}}_{1}( \boldsymbol{\ell }) \\ &\quad{}\times \frac{S(t-\boldsymbol{\ell })V(t-\boldsymbol{\ell })L_{7}}{L}\,d \boldsymbol{\ell }-\vartheta _{2} \lambda \mathcal{H}_{3} ( 1-\beta ) \int _{0}^{\kappa _{1}}\bar{\mathcal{H}}_{1}( \boldsymbol{\ell })\frac{S(t-\boldsymbol{\ell })I(t-\boldsymbol{\ell })L_{7}}{L}\,d \boldsymbol{\ell } \\ &\quad{}+\lambda \mathcal{H}_{3} ( \lambda +\gamma ) L_{7}-a ( \lambda +\gamma ) I-\vartheta _{1}\beta ( \gamma +\lambda ) \int _{0}^{\kappa _{2}} \bar{\mathcal{H}}_{2}( \boldsymbol{\ell }) \frac{S(t-\boldsymbol{\ell })V(t-\boldsymbol{\ell })I_{7}}{I}\,d\boldsymbol{\ell } \\ &\quad{}-\vartheta _{2}\beta ( \gamma +\lambda ) \int _{0}^{\kappa _{2}}\bar{\mathcal{H}}_{2}(\boldsymbol{\ell }) \frac{S(t-\boldsymbol{\ell })I(t-\boldsymbol{\ell })I_{7}}{I}\,d\boldsymbol{\ell }\\ &\quad {}- \lambda ( \lambda +\gamma ) \int _{0}^{\kappa _{3}} \bar{\mathcal{H}}_{3}( \boldsymbol{\ell })\frac{L(t-\boldsymbol{\ell })I_{7}}{I}\,d\boldsymbol{\ell }+a ( \lambda +\gamma ) I_{7}+\mu _{1} ( \lambda + \gamma ) C^{I}I_{7} \\ &\quad{}+ \frac{\mathcal{P}r}{\varphi \mathcal{H}_{4}}Y- \frac{\mathcal{P}\vartheta _{3}}{\mathcal{H}_{4}} \int _{0}^{ \kappa _{4}}\bar{\mathcal{H}}_{4}( \boldsymbol{\ell }) \frac{S(t-\boldsymbol{\ell })Y(t-\boldsymbol{\ell })E_{7}}{E}\,d\boldsymbol{\ell }-\frac{\mathcal{P}r}{\varphi \mathcal{H}_{4}}\frac{YE_{7}}{E} \\ &\quad{}+ \frac{\mathcal{P} ( \psi +\omega ) }{\varphi \mathcal{H}_{4}}E_{7}- \frac{\mathcal{P}\delta ( \psi +\omega ) }{\varphi \psi \mathcal{H}_{4}\mathcal{H}_{5}}Y- \frac{\mathcal{P} ( \psi +\omega ) }{\varphi \mathcal{H}_{4}\mathcal{H}_{5}} \int _{0}^{\kappa _{5}}\bar{\mathcal{H}}_{5}( \boldsymbol{\ell }) \frac{E(t-\boldsymbol{\ell })Y_{7}}{Y}\,d\boldsymbol{\ell } \\ &\quad{}+ \frac{\mathcal{P}\delta ( \psi +\omega ) }{\varphi \psi \mathcal{H}_{4}\mathcal{H}_{5}}Y_{7}+ \frac{\mu _{2}\mathcal{P} ( \psi +\omega ) }{\varphi \psi \mathcal{H}_{4}\mathcal{H}_{5}}C^{Y}Y_{7}- \frac{b\mathcal{P}\vartheta _{1}S_{7}}{\varepsilon } \int _{0}^{\kappa _{6}}\bar{\mathcal{H}}_{6}(\boldsymbol{\ell }) \frac{I(t-\boldsymbol{\ell })V_{7}}{V}\,d\boldsymbol{\ell } \\ &\quad{}+\mathcal{P}\vartheta _{1}S_{7}V_{7}- \frac{\mu _{1}\pi _{1} ( \lambda +\gamma ) }{\sigma _{1}}C^{I}-\mu _{1} ( \lambda + \gamma ) C_{7}^{I}I+ \frac{\mu _{1}\pi _{1} ( \lambda +\gamma ) }{\sigma _{1}}C_{7}^{I} \\ &\quad{}- \frac{\mu _{2}\pi _{2}\mathcal{P} ( \psi +\omega ) }{\sigma _{2}\varphi \psi \mathcal{H}_{4}\mathcal{H}_{5}}C^{Y}- \frac{\mu _{2}\mathcal{P} ( \psi +\omega ) }{\varphi \psi \mathcal{H}_{4}\mathcal{H}_{5}}C_{7}^{Y}Y+ \frac{\mu _{2}\pi _{2}\mathcal{P} ( \psi +\omega ) }{\sigma _{2}\varphi \psi \mathcal{H}_{4}\mathcal{H}_{5}}C_{7}^{Y} \\ &\quad{}+\lambda \mathcal{H}_{3} ( 1-\beta ) \vartheta _{1}S_{7}V_{7}\int _{0}^{\kappa _{1}}\bar{\mathcal{H}}_{1}( \boldsymbol{\ell }) \ln \biggl( \frac{S(t-\boldsymbol{\ell })V(t-\boldsymbol{\ell })}{SV} \biggr) \,d \boldsymbol{\ell }\\ &\quad {}+\lambda \mathcal{H}_{3} ( 1-\beta ) \vartheta _{2}S_{7}I_{7}\int _{0}^{\kappa _{1}}\bar{\mathcal{H}}_{1}( \boldsymbol{\ell }) \ln \biggl( \frac{S(t-\boldsymbol{\ell })I(t-\boldsymbol{\ell })}{SI} \biggr) \,d\boldsymbol{\ell }\\ &\quad {}+\beta ( \gamma +\lambda ) \vartheta _{1}S_{7}V_{7}\int _{0}^{\kappa _{2}}\bar{\mathcal{H}}_{2}( \boldsymbol{\ell }) \ln \biggl( \frac{S(t-\boldsymbol{\ell })V(t-\boldsymbol{\ell })}{SV} \biggr) \,d \boldsymbol{\ell } \\ &\quad{}+\beta ( \gamma +\lambda ) \vartheta _{2}S_{7}I_{7} \int _{0}^{\kappa _{2}}\bar{\mathcal{H}}_{2}( \boldsymbol{\ell }) \ln \biggl( \frac{S(t-\boldsymbol{\ell })I(t-\boldsymbol{\ell })}{SI} \biggr) \,d \boldsymbol{\ell }\\ &\quad {}+\lambda ( \gamma +\lambda ) L_{7} \int _{0}^{ \kappa _{3}}\bar{\mathcal{H}}_{3}( \boldsymbol{\ell }) \ln \biggl( \frac{L(t-\boldsymbol{\ell })}{L} \biggr) \,d \boldsymbol{\ell }\\ &\quad {}+\frac{\mathcal{P}\vartheta _{3}S_{7}Y_{7}}{\mathcal{H}_{4}} \int _{0}^{\kappa _{4}}\bar{\mathcal{H}}_{4}(\boldsymbol{\ell })\ln \biggl( \frac{S(t-\boldsymbol{\ell })Y(t-\boldsymbol{\ell })}{SY} \biggr) \,d \boldsymbol{\ell }\\ &\quad {}+ \frac{\mathcal{P} ( \psi +\omega ) E_{7}}{\varphi \mathcal{H}_{4}\mathcal{H}_{5}} \int _{0}^{\kappa _{5}}\bar{\mathcal{H}}_{5}( \boldsymbol{\ell })\ln \biggl( \frac{E(t-\boldsymbol{\ell })}{E} \biggr) \,d \boldsymbol{\ell }+ \frac{b\mathcal{P}\vartheta _{1}S_{7}\mathcal{H}_{6}}{\varepsilon }I\\ &\quad {}+ \frac{b\mathcal{P}\vartheta _{1}S_{7}I_{7}}{\varepsilon } \int _{0}^{\kappa _{6}}\bar{\mathcal{H}}_{6}( \boldsymbol{\ell })\ln \biggl( \frac{I(t-\boldsymbol{\ell })}{I} \biggr) \,d\boldsymbol{\ell }. \end{aligned}$$

Using the steady state conditions for

$$\begin{aligned} & \eta =\varrho S_{7}+\vartheta _{1}S_{7}V_{7}+ \vartheta _{2}S_{7}I_{7}+\vartheta _{3}S_{7}Y_{7}, \\ &\mathcal{H}_{1} ( 1-\beta ) ( \vartheta _{1}S_{7}V_{7}+\vartheta _{2}S_{7}I_{7} ) = ( \lambda +\gamma ) L_{7}, \\ & \beta \mathcal{H}_{2} ( \vartheta _{1}S_{7}V_{7}+ \vartheta _{2}S_{7}I_{7} ) +\lambda \mathcal{H}_{3}L_{7}= \bigl( a+\mu _{1}C_{7}^{I} \bigr) I_{7}, \\ & I_{7}= \frac{\pi _{1}}{\sigma _{1}}, \qquad Y_{7}= \frac{\pi _{2}}{\sigma _{2}}, \qquad V_{7}= \frac{b\mathcal{H}_{6}I_{7}}{\varepsilon }, \\ & \vartheta _{3}S_{7}Y_{7}+ \frac{rY_{7}}{\varphi \mathcal{H}_{4}}=\frac{ ( \psi +\omega ) E_{7}}{\varphi \mathcal{H}_{4}}= \frac{\delta ( \psi +\omega ) }{\varphi \psi \mathcal{H}_{4}\mathcal{H}_{5}}Y_{7}+ \frac{\mu _{2} ( \psi +\omega ) }{\varphi \psi \mathcal{H}_{4}\mathcal{H}_{5}}C_{7}^{Y}Y_{7}, \end{aligned}$$

we get

$$ \mathcal{P} ( \vartheta _{1}S_{7}V_{7}+ \vartheta _{2}S_{7}I_{7} ) = ( \lambda +\gamma ) \bigl( a+\mu _{1}C_{7}^{I} \bigr) I_{7}. $$

Moreover, we get

$$\begin{aligned} \frac{d\Phi _{7}}{dt} & =\mathcal{P} \biggl( 1-\frac{S_{7}}{S} \biggr) ( \varrho S_{7}-\varrho S ) +\mathcal{P} ( \vartheta _{1}S_{7}V_{7}+ \vartheta _{2}S_{7}I_{7}+\vartheta _{3}S_{7}Y_{7} ) \biggl( 1-\frac{S_{7}}{S} \biggr) \\ &\quad{}-\lambda \mathcal{H}_{3} ( 1-\beta ) \vartheta _{1}S_{7}V_{7}\int _{0}^{\kappa _{1}}\bar{\mathcal{H}}_{1}( \boldsymbol{\ell }) \frac{S(t-\boldsymbol{\ell })V(t-\boldsymbol{\ell })L_{7}}{S_{7}V_{7}L}\,d \boldsymbol{\ell }\\ &\quad {}-\lambda \mathcal{H}_{3} ( 1-\beta ) \vartheta _{2}S_{7}I_{7} \int _{0}^{\kappa _{1}}\bar{\mathcal{H}}_{1}( \boldsymbol{\ell })\frac{S(t-\boldsymbol{\ell })I(t-\boldsymbol{\ell })L_{7}}{S_{7}I_{7}L}\,d \boldsymbol{\ell }\\ &\quad {}+\lambda \mathcal{H}_{1}\mathcal{H}_{3} ( 1-\beta ) ( \vartheta _{1}S_{7}V_{7}+\vartheta _{2}S_{7}I_{7} ) \\ &\quad{}-\beta ( \gamma +\lambda ) \vartheta _{1}S_{7}V_{7} \int _{0}^{\kappa _{2}}\bar{\mathcal{H}}_{2}( \boldsymbol{\ell }) \frac{S(t-\boldsymbol{\ell })V(t-\boldsymbol{\ell })I_{7}}{S_{7}V_{7}I}\,d \boldsymbol{\ell }\\ &\quad {}-\beta ( \gamma +\lambda ) \vartheta _{2}S_{7}I_{7} \int _{0}^{\kappa _{2}}\bar{\mathcal{H}}_{2}( \boldsymbol{\ell }) \frac{S(t-\boldsymbol{\ell })I(t-\boldsymbol{\ell })}{S_{7}I}\,d \boldsymbol{\ell }\\ &\quad {}-\lambda \mathcal{H}_{1} ( 1-\beta ) ( \vartheta _{1}S_{7}V_{7}+ \vartheta _{2}S_{7}I_{7} ) \int _{0}^{ \kappa _{3}}\bar{\mathcal{H}}_{3}( \boldsymbol{\ell }) \frac{L(t-\boldsymbol{\ell })I_{7}}{L_{7}I}\,d\boldsymbol{\ell } \\ &\quad{}+\mathcal{P} ( \vartheta _{1}S_{7}V_{7}+ \vartheta _{2}S_{7}I_{7} ) -\frac{\mathcal{P}\vartheta _{3}S_{7}Y_{7}}{\mathcal{H}_{4}} \int _{0}^{\kappa _{4}}\bar{\mathcal{H}}_{4}(\boldsymbol{\ell }) \frac{S(t-\boldsymbol{\ell })Y(t-\boldsymbol{\ell })E_{7}}{S_{7}Y_{7}E}\,d \boldsymbol{\ell }\\ &\quad {}-\frac{\mathcal{P}rY_{7}}{\varphi \mathcal{H}_{4}}\frac{YE_{7}}{Y_{7}E}+\mathcal{P}\vartheta _{3}S_{7}Y_{7}+ \frac{\mathcal{P}rY_{7}}{\varphi \mathcal{H}_{4}} \\ &\quad{}- \biggl( \frac{\mathcal{P}\vartheta _{3}S_{7}Y_{7}}{\mathcal{H}_{5}}+ \frac{\mathcal{P}rY_{7}}{\varphi \mathcal{H}_{4}\mathcal{H}_{5}} \biggr) \int _{0}^{\kappa _{5}} \bar{\mathcal{H}}_{5}( \boldsymbol{\ell })\frac{E(t-\boldsymbol{\ell })Y_{7}}{E_{7}Y}\,d \boldsymbol{\ell } \\ &\quad{}+\mathcal{P}\vartheta _{3}S_{7}Y_{7}+ \frac{\mathcal{P}rY_{7}}{\varphi \mathcal{H}_{4}}Y_{7}- \frac{\mathcal{P}\vartheta _{1}S_{7}V_{7}}{\mathcal{H}_{6}} \int _{0}^{\kappa _{6}}\bar{\mathcal{H}}_{6}( \boldsymbol{\ell })\frac{I(t-\boldsymbol{\ell })V_{7}}{I_{7}V}\,d\boldsymbol{\ell } \\ &\quad{}+\mathcal{P}\vartheta _{1}S_{7}V_{7}+ \lambda \mathcal{H}_{3} ( 1- \beta ) \vartheta _{1}S_{7}V_{7} \int _{0}^{\kappa _{1}}\bar{\mathcal{H}}_{1}( \boldsymbol{\ell })\ln \biggl( \frac{S(t-\boldsymbol{\ell })V(t-\boldsymbol{\ell })}{SV} \biggr) \,d \boldsymbol{\ell } \\ &\quad{}+\lambda \mathcal{H}_{3} ( 1-\beta ) \vartheta _{2}S_{7}I_{7}\int _{0}^{\kappa _{1}}\bar{\mathcal{H}}_{1}( \boldsymbol{\ell }) \ln \biggl( \frac{S(t-\boldsymbol{\ell })I(t-\boldsymbol{\ell })}{SI} \biggr) \,d \boldsymbol{\ell }\\ &\quad {}+\beta ( \gamma +\lambda ) \vartheta _{1}S_{7}V_{7} \int _{0}^{\kappa _{2}}\bar{\mathcal{H}}_{2}( \boldsymbol{\ell }) \ln \biggl( \frac{S(t-\boldsymbol{\ell })V(t-\boldsymbol{\ell })}{SV} \biggr) \,d\boldsymbol{\ell }\\ &\quad {}+\beta ( \gamma +\lambda ) \vartheta _{2}S_{7}I_{7}\int _{0}^{\kappa _{2}}\bar{\mathcal{H}}_{2}( \boldsymbol{\ell }) \ln \biggl( \frac{S(t-\boldsymbol{\ell })I(t-\boldsymbol{\ell })}{SI} \biggr) \,d \boldsymbol{\ell } \\ &\quad{}+\lambda \mathcal{H}_{1} ( 1-\beta ) ( \vartheta _{1}S_{7}V_{7}+\vartheta _{2}S_{7}I_{7} ) \int _{0}^{ \kappa _{3}}\bar{\mathcal{H}}_{3}( \boldsymbol{\ell })\ln \biggl( \frac{L(t-\boldsymbol{\ell })}{L} \biggr) \,d\boldsymbol{\ell }\\ &\quad {}+ \frac{\mathcal{P}\vartheta _{3}S_{7}Y_{7}}{\mathcal{H}_{4}} \int _{0}^{\kappa _{4}}\bar{\mathcal{H}}_{4}( \boldsymbol{\ell }) \ln \biggl( \frac{S(t-\boldsymbol{\ell })Y(t-\boldsymbol{\ell })}{SY} \biggr) \,d\boldsymbol{\ell }\\ &\quad {}+ \biggl( \frac{\mathcal{P}\vartheta _{3}S_{7}Y_{7}}{\mathcal{H}_{5}}+ \frac{\mathcal{P}rY_{7}}{\varphi \mathcal{H}_{4}\mathcal{H}_{5}} \biggr) \int _{0}^{\kappa _{5}} \bar{\mathcal{H}}_{5}( \boldsymbol{\ell })\ln \biggl( \frac{E(t-\boldsymbol{\ell })}{E} \biggr) \,d \boldsymbol{\ell } \\ &\quad{}+\frac{\mathcal{P}\vartheta _{1}S_{7}V_{7}}{\mathcal{H}_{6}} \int _{0}^{\kappa _{6}}\bar{\mathcal{H}}_{6}( \boldsymbol{\ell })\ln \biggl( \frac{I(t-\boldsymbol{\ell })}{I} \biggr) \,d\boldsymbol{\ell }. \end{aligned}$$

Using the equalities given by (10) and (11) in case of $n=m=7$, we get

28 $$\begin{aligned} \frac{d\Phi _{7}}{dt} & =-\varrho \mathcal{P} \frac{(S-S_{7})^{2}}{S}-\mathcal{P} ( \vartheta _{1}S_{7}V_{7}+\vartheta _{2}S_{7}I_{7}+\vartheta _{3}S_{7}Y_{7} ) \biggl[ \frac{S_{7}}{S}-1- \ln \biggl( \frac{S_{7}}{S} \biggr) \biggr] \\ &\quad{}-\lambda \mathcal{H}_{3} ( 1-\beta ) \vartheta _{1}S_{7}V_{7} \\ &\quad {}\times\int _{0}^{\kappa _{1}}\bar{\mathcal{H}}_{1}( \boldsymbol{\ell }) \biggl[ \frac{S(t-\boldsymbol{\ell })V(t-\boldsymbol{\ell })L_{7}}{S_{7}V_{7}L}-1- \ln \biggl( \frac{S(t-\boldsymbol{\ell })V(t-\boldsymbol{\ell })L_{7}}{S_{7}V_{7}L} \biggr) \biggr] \,d\boldsymbol{\ell } \\ &\quad{}-\lambda \mathcal{H}_{3} ( 1-\beta ) \vartheta _{2}S_{7}I_{7} \\ &\quad {}\times\int _{0}^{\kappa _{1}}\bar{\mathcal{H}}_{1}( \boldsymbol{\ell }) \biggl[ \frac{S(t-\boldsymbol{\ell })I(t-\boldsymbol{\ell })L_{7}}{S_{7}I_{7}L}-1- \ln \biggl( \frac{S(t-\boldsymbol{\ell })I(t-\boldsymbol{\ell })L_{7}}{S_{7}I_{7}L} \biggr) \biggr] \,d\boldsymbol{\ell } \\ &\quad{}-\beta ( \gamma +\lambda ) \vartheta _{1}S_{7}V_{7} \\ &\quad {}\times\int _{0}^{\kappa _{2}}\bar{\mathcal{H}}_{2}( \boldsymbol{\ell }) \biggl[ \frac{S(t-\boldsymbol{\ell })V(t-\boldsymbol{\ell })I_{7}}{S_{7}V_{7}I}-1- \ln \biggl( \frac{S(t-\boldsymbol{\ell })V(t-\boldsymbol{\ell })I_{7}}{S_{7}V_{7}I} \biggr) \biggr] \,d\boldsymbol{\ell } \\ &\quad{}-\beta ( \gamma +\lambda ) \vartheta _{2}S_{7}I_{7} \int _{0}^{\kappa _{2}}\bar{\mathcal{H}}_{2}( \boldsymbol{\ell }) \biggl[ \frac{S(t-\boldsymbol{\ell })I(t-\boldsymbol{\ell })}{S_{7}I}-1-\ln \biggl( \frac{S(t-\boldsymbol{\ell })I(t-\boldsymbol{\ell })}{S_{7}I} \biggr) \biggr] \,d\boldsymbol{\ell } \\ &\quad{}-\lambda \mathcal{H}_{1} ( 1-\beta ) ( \vartheta _{1}S_{7}V_{7}+\vartheta _{2}S_{7}I_{7} ) \\ &\quad {}\times\int _{0}^{ \kappa _{3}}\bar{\mathcal{H}}_{3}( \boldsymbol{\ell })) \biggl[ \frac{L(t-\boldsymbol{\ell })I_{7}}{L_{7}I}-1-\ln \biggl( \frac{L(t-\boldsymbol{\ell })I_{7}}{L_{7}I} \biggr) \biggr] \,d\boldsymbol{\ell } \\ &\quad{}-\frac{\mathcal{P}\vartheta _{3}S_{7}Y_{7}}{\mathcal{H}_{4}} \int _{0}^{\kappa _{4}}\bar{\mathcal{H}}_{4}( \boldsymbol{\ell }) \biggl[ \frac{S(t-\boldsymbol{\ell })Y(t-\boldsymbol{\ell })E_{7}}{S_{7}Y_{7}E}-1-\ln \biggl( \frac{S(t-\boldsymbol{\ell })Y(t-\boldsymbol{\ell })E_{7}}{S_{7}Y_{7}E} \biggr) \biggr] \,d\boldsymbol{\ell } \\ &\quad{}-\frac{\mathcal{P}rY_{7}}{\varphi \mathcal{H}_{4}} \biggl[ \frac{YE_{7}}{Y_{7}E}-1-\ln \biggl( \frac{YE_{7}}{Y_{7}E} \biggr) \biggr] \\ &\quad{}- \biggl( \frac{\mathcal{P}\vartheta _{3}S_{7}Y_{7}}{\mathcal{H}_{5}}+\frac{\mathcal{P}rY_{7}}{\varphi \mathcal{H}_{4}\mathcal{H}_{5}} \biggr) \int _{0}^{\kappa _{5}}\bar{\mathcal{H}}_{5}( \boldsymbol{\ell }) \biggl[ \frac{E(t-\boldsymbol{\ell })Y_{7}}{E_{7}Y}-1-\ln \biggl( \frac{E(t-\boldsymbol{\ell })Y_{7}}{E_{7}Y} \biggr) \biggr] \,d \boldsymbol{\ell } \\ &\quad{}-\frac{\mathcal{P}\vartheta _{1}S_{7}V_{7}}{\mathcal{H}_{6}} \int _{0}^{\kappa _{6}}\bar{\mathcal{H}}_{6}( \boldsymbol{\ell }) \biggl[ \frac{I(t-\boldsymbol{\ell })V_{7}}{I_{7}V}-1-\ln \biggl( \frac{I(t-\boldsymbol{\ell })V_{7}}{I_{7}V} \biggr) \biggr] \,d \boldsymbol{\ell }. \end{aligned}$$

Therefore, Eq. (28) becomes

$$\begin{aligned} \frac{d\Phi _{7}}{dt} & =-\varrho \mathcal{P} \frac{(S-S_{7})^{2}}{S}-\mathcal{P} ( \vartheta _{1}S_{7}V_{7}+\vartheta _{2}S_{7}I_{7}+\vartheta _{3}S_{7}Y_{7} ) \digamma \biggl( \frac{S_{7}}{S} \biggr) \\ &\quad{}-\lambda \mathcal{H}_{3} ( 1-\beta ) \vartheta _{1}S_{7}V_{7}\int _{0}^{\kappa _{1}}\bar{\mathcal{H}}_{1}( \boldsymbol{\ell }) \digamma \biggl( \frac{S(t-\boldsymbol{\ell })V(t-\boldsymbol{\ell })L_{7}}{S_{7}V_{7}L} \biggr) \,d \boldsymbol{ \ell } \\ &\quad{}-\lambda \mathcal{H}_{3} ( 1-\beta ) \vartheta _{2}S_{7}I_{7}\int _{0}^{\kappa _{1}}\bar{\mathcal{H}}_{1}( \boldsymbol{\ell }) \digamma \biggl( \frac{S(t-\boldsymbol{\ell })I(t-\boldsymbol{\ell })L_{7}}{S_{7}I_{7}L} \biggr) \,d \boldsymbol{ \ell } \\ &\quad{}-\beta ( \gamma +\lambda ) \vartheta _{1}S_{7}V_{7} \int _{0}^{\kappa _{2}}\bar{\mathcal{H}}_{2}( \boldsymbol{\ell }) \digamma \biggl( \frac{S(t-\boldsymbol{\ell })V(t-\boldsymbol{\ell })I_{7}}{S_{7}V_{7}I} \biggr) \,d \boldsymbol{ \ell } \\ &\quad{}-\beta ( \gamma +\lambda ) \vartheta _{2}S_{7}I_{7} \int _{0}^{\kappa _{2}}\bar{\mathcal{H}}_{2}( \boldsymbol{\ell }) \digamma \biggl( \frac{S(t-\boldsymbol{\ell })I(t-\boldsymbol{\ell })}{S_{7}I} \biggr) \,d\boldsymbol{\ell } \\ &\quad{}-\lambda \mathcal{H}_{1} ( 1-\beta ) ( \vartheta _{1}S_{7}V_{7}+\vartheta _{2}S_{7}I_{7} ) \int _{0}^{ \kappa _{3}}\bar{\mathcal{H}}_{3}( \boldsymbol{\ell }))\digamma \biggl( \frac{L(t-\boldsymbol{\ell })I_{7}}{L_{7}I} \biggr) \,d\boldsymbol{\ell } \\ &\quad{}-\frac{\mathcal{P}\vartheta _{3}S_{7}Y_{7}}{\mathcal{H}_{4}} \int _{0}^{\kappa _{4}}\bar{\mathcal{H}}_{4}( \boldsymbol{\ell }) \digamma \biggl( \frac{S(t-\boldsymbol{\ell })Y(t-\boldsymbol{\ell })E_{7}}{S_{7}Y_{7}E} \biggr) \,d \boldsymbol{ \ell }-\frac{\mathcal{P}rY_{7}}{\varphi \mathcal{H}_{4}} \digamma \biggl( \frac{YE_{7}}{Y_{7}E} \biggr) \\ &\quad{}- \biggl( \frac{\mathcal{P}\vartheta _{3}S_{7}Y_{7}}{\mathcal{H}_{5}}+\frac{\mathcal{P}rY_{7}}{\varphi \mathcal{H}_{4}\mathcal{H}_{5}} \biggr) \int _{0}^{\kappa _{5}}\bar{\mathcal{H}}_{5}( \boldsymbol{\ell })\digamma \biggl( \frac{E(t-\boldsymbol{\ell })Y_{7}}{E_{7}Y} \biggr) \,d\boldsymbol{\ell } \\ &\quad{}-\frac{\mathcal{P}\vartheta _{1}S_{7}V_{7}}{\mathcal{H}_{6}} \int _{0}^{\kappa _{6}}\bar{\mathcal{H}}_{6}( \boldsymbol{\ell }) \digamma \biggl( \frac{I(t-\boldsymbol{\ell })V_{7}}{I_{7}V} \biggr) \,d \boldsymbol{ \ell }. \end{aligned}$$

Hence, $\frac{d\Phi _{7}}{dt}\leq 0$ for all $S,L,I,E,Y,V>0$. Similar to the previous theorems, one can show that $\frac{d\Phi _{7}}{dt}=0$ when $(S,L,I,E,Y,V)=(S_{7},L_{7},I_{7},E_{7},Y_{7},V_{7})$. The solutions of system (5) converge to $\Upsilon _{7}^{{\prime }}$ which includes elements with $(S,L,I,E,Y,V)(t)=(S_{7},L_{7},I_{7},E_{7},Y_{7},V_{7})$. Then $\frac{dI(t)}{dt}=\frac{dY(t)}{dt}=0$. The third and fifth equations of system (5) become

$$\begin{aligned} &0 =\frac{dI(t)}{dt}=\beta \mathcal{H}_{2} ( \vartheta _{1}S_{7}V_{7}+\vartheta _{2}S_{7}I_{7} ) +\lambda \mathcal{H}_{3}L_{7}-aI_{7}- \mu _{1}C^{I}(t)I_{7}, \\ &0 =\frac{dY(t)}{dt}=\psi \mathcal{H}_{5}E_{7}-\delta Y_{7}-\mu _{2}C^{Y}(t)Y_{7}, \end{aligned}$$

which yield $C^{I}(t)=C_{7}^{I}$ and $C^{Y}(t)=C_{7}^{Y}$ for all *t*, and hence . Applying LLAS theorem, we get  is GAS. □

## Numerical simulations

In this section, we perform numerical simulations to illustrate the results of Theorems 1–8. Moreover, we study the influence of time delays on the dynamical behavior of the system. Let us choose a Dirac delta function $D(\cdot)$ as a special form of the kernel $\Lambda _{i}(\cdot)$ as follows:

$$ \Lambda _{i}(x)=D ( x-\boldsymbol{\ell }_{i} ) , \quad \boldsymbol{\ell }_{i}\in [ 0,\kappa _{i} ] , i=1,2,\ldots,6. $$

Let $\kappa _{i}\rightarrow \infty $, then we get

$$\begin{aligned}& \int _{0}^{\infty }\Lambda _{j}(\varsigma )\,d \varsigma =1, \\& \mathcal{H}_{j}= \int _{0}^{\infty }D ( \varsigma -\boldsymbol{\ell }_{j} ) e^{-\hslash _{j}\varsigma }\,d \varsigma =e^{-\hslash _{j}\boldsymbol{\ell }_{j}}, \quad j=1,2,\ldots,6. \end{aligned}$$

Thus, model (4) reduces to

29 $$ \textstyle\begin{cases}\frac{dS(t)}{dt}=\eta -\varrho S(t)-\vartheta _{1}S(t)V(t)-\vartheta _{2}S(t)I(t)- \vartheta _{3}S(t)Y(t), \\ \frac{dL(t)}{dt}= ( 1-\beta ) e^{-\hslash _{1} \boldsymbol{\ell }_{1}}S(t-\boldsymbol{\ell }_{1}) [ \vartheta _{1}V(t-\boldsymbol{\ell }_{1})+\vartheta _{2}I(t-\boldsymbol{\ell }_{1}) ] - ( \lambda + \gamma ) L(t), \\ \frac{dI(t)}{dt}=\beta e^{-\hslash _{2}\boldsymbol{\ell }_{2}}S(t- \boldsymbol{\ell }_{2}) [ \vartheta _{1}V(t-\boldsymbol{\ell }_{2})+\vartheta _{2}I(t- \boldsymbol{\ell }_{2}) ] \\ \hphantom{\frac{dI(t)}{dt}=}{} +\lambda e^{-\hslash _{3}\boldsymbol{\ell }_{3}}L(t-\boldsymbol{\ell }_{3})-aI(t)-\mu _{1}C^{I}(t)I(t), \\ \frac{dE(t)}{dt}=\varphi \vartheta _{3}e^{-\hslash _{4}\boldsymbol{\ell }_{4}}S(t-\boldsymbol{\ell }_{4})Y(t-\boldsymbol{\ell }_{4})+\mathcal{K}r^{\ast }Y(t)- ( \psi +\omega ) E(t), \\ \frac{dY(t)}{dt}=\psi e^{-\hslash _{5}\boldsymbol{\ell }_{5}}E(t- \boldsymbol{\ell }_{5})+ ( 1-\mathcal{K} ) r^{\ast }Y(t)-\delta ^{\ast }Y(t)- \mu _{2}C^{Y}(t)Y(t), \\ \frac{dV(t)}{dt}=be^{-\hslash _{6}\boldsymbol{\ell }_{6}}I(t- \boldsymbol{\ell }_{6})-\varepsilon V(t), \\ \frac{dC^{I}(t)}{dt}=\sigma _{1}C^{I}(t)I(t)-\pi _{1}C^{I}(t), \\ \frac{dC^{Y}(t)}{dt}=\sigma _{2}C^{Y}(t)Y(t)-\pi _{2}C^{Y}(t). \end{cases} $$

For model (29), the threshold parameters are given by

30 $$\begin{aligned} &\Re _{1} = \frac{\mathcal{P}S_{0} ( b\vartheta _{1}e^{-\hslash _{6}\boldsymbol{\ell }_{6}}+\varepsilon \vartheta _{2} ) }{a\varepsilon ( \gamma +\lambda ) }, \qquad \Re _{2}= \frac{\varphi \vartheta _{3}\psi e^{- ( \hslash _{4}\boldsymbol{\ell }_{4}+\hslash _{5}\boldsymbol{\ell }_{5} ) }S_{0}}{ ( \delta -re^{-\hslash _{5}\boldsymbol{\ell }_{5}} ) \psi +\delta \omega }, \\ &\Re _{3} = \frac{\sigma _{1}\eta \mathcal{P} ( b\vartheta _{1}e^{-\hslash _{6}\boldsymbol{\ell }_{6}}+\varepsilon \vartheta _{2} ) }{a ( \gamma +\lambda ) [ \pi _{1} ( b\vartheta _{1}e^{-\hslash _{6}\boldsymbol{\ell }_{6}}+\varepsilon \vartheta _{2} ) +\varrho \varepsilon \sigma _{1} ] }, \\ &\Re _{4}= \frac{\sigma _{2}\eta \varphi \vartheta _{3}\psi e^{- ( \hslash _{4}\boldsymbol{\ell }_{4}+\hslash _{5}\boldsymbol{\ell }_{5} ) }}{ ( \pi _{2}\vartheta _{3}+\varrho \sigma _{2} ) [ (\delta -re^{-\hslash _{5}\boldsymbol{\ell }_{5}})\psi +\delta \omega ] }, \\ &\Re _{5} = \frac{\eta \varphi \varepsilon \vartheta _{3}\sigma _{1}\psi e^{- ( \hslash _{4}\boldsymbol{\ell }_{4}+\hslash _{5}\boldsymbol{\ell }_{5} ) }}{ [ ( \delta -re^{-\hslash _{5}\boldsymbol{\ell }_{5}} ) \psi +\delta \omega ] [ \pi _{1} ( b\vartheta _{1}e^{-\hslash _{6}\boldsymbol{\ell }_{6}}+\varepsilon \vartheta _{2} ) +\varrho \varepsilon \sigma _{1} ] }, \\ &\Re _{6} = \frac{\eta \sigma _{2}\mathcal{P} ( b\vartheta _{1}e^{-\hslash _{6}\boldsymbol{\ell }_{6}}+\varepsilon \vartheta _{2} ) }{a\varepsilon (\gamma +\lambda )(\pi _{2}\vartheta _{3}+\varrho \sigma _{2})}, \\ &\Re _{7}= \frac{\sigma _{1}\sigma _{2}\eta \mathcal{P} ( b\vartheta _{1}e^{-\hslash _{6}\boldsymbol{\ell }_{6}}+\varepsilon \vartheta _{2} ) }{a ( \gamma +\lambda ) [ \pi _{1}\sigma _{2} ( b\vartheta _{1}e^{-\hslash _{6}\boldsymbol{\ell }_{6}}+\varepsilon \vartheta _{2} ) +\varepsilon \sigma _{1} ( \pi _{2}\vartheta _{3}+\varrho \sigma _{2} ) ] }, \\ &\Re _{8} = \frac{\varepsilon \vartheta _{3}\eta \sigma _{1}\sigma _{2}\varphi \psi e^{- ( \hslash _{4}\boldsymbol{\ell }_{4}+\hslash _{5}\boldsymbol{\ell }_{5} ) }}{ [ \pi _{1}\sigma _{2} ( b\vartheta _{1}e^{-\hslash _{6}\boldsymbol{\ell }_{6}}+\varepsilon \vartheta _{2} ) +\varepsilon \sigma _{1} ( \pi _{2}\vartheta _{3}+\varrho \sigma _{2} ) ] [ (\delta -re^{-\hslash _{5}\boldsymbol{\ell }_{5}})\psi +\delta \omega ] }, \end{aligned}$$

where

31 $$ \mathcal{P}=\lambda e^{- ( \hslash _{1}\boldsymbol{\ell }_{1}+ \hslash _{3}\boldsymbol{\ell }_{3} ) } ( 1-\beta ) + \beta e^{-\hslash _{2}\boldsymbol{\ell }_{2}} ( \gamma +\lambda ) . $$

To solve system (29) numerically, we fix the values of some parameters (see Table 1) and the others will be varied.

**Table 1:** The data of model (29)

Parameter	Value	Parameter	Value	Parameter	Value
*η*	10	*b*	5	*ω*	0.03
*ϱ*	0.01	$\pi _{1}$	0.1	*ψ*	0.003
$\vartheta _{i}$, *i* = 1,2,3	Varied	$\pi _{2}$	0.1	$\hslash _{1}$	0.2
*β*	0.7	$\mu _{1}$	0.2	$\hslash _{2}$	0.3
*a*	0.5	$\mu _{2}$	0.2	$\hslash _{3}$	0.4
*φ*	0.2	*ε*	2	$\hslash _{4}$	0.5
$\mathcal{K}$	0.9	*γ*	0.1	$\hslash _{5}$	0.6
$r^{\ast }$	0.008	$\sigma _{i}$, *i* = 1,2	Varied	$\hslash _{6}$	0.9
$\delta ^{\ast }$	0.05	*λ*	0.2	$\xi _{i}$, *i* = 1,2,…,6	Varied

### Stability of the steady states

In this subsection, we select the delay parameters as $\boldsymbol{\ell }_{1}=1$, $\boldsymbol{\ell }_{2}=0.8$, $\boldsymbol{\ell }_{3}=0.6$, $\boldsymbol{\ell }_{4}=0.4$, $\boldsymbol{\ell }_{5}=0.2$, $\boldsymbol{\ell }_{6}=0.1$. Besides, we choose the following three different initial conditions for system (29):

*Initial-1*: $(S(\boldsymbol{\ell }),L(\boldsymbol{\ell }),I(\boldsymbol{\ell }),E(\boldsymbol{\ell }),Y(\boldsymbol{\ell }),V(\boldsymbol{\ell }),C^{I}(\boldsymbol{\ell }),C^{Y}(\boldsymbol{\ell }))= ( 800,0.5,0.7,12,0.51.52.5,0.3 ) $,

*Initial-2*: $(S(\boldsymbol{\ell }),L(\boldsymbol{\ell }),I(\boldsymbol{\ell }),E(\boldsymbol{\ell }),Y(\boldsymbol{\ell }),V(\boldsymbol{\ell }),C^{I}(\boldsymbol{\ell }),C^{Y}(\boldsymbol{\ell }))= ( 700,0.8,1,8,0.3,2.5,1.5,0.2 ) $,

*Initial-3*: $(S(\boldsymbol{\ell }),L(\boldsymbol{\ell }),I(\boldsymbol{\ell }),E(\boldsymbol{\ell }),Y(\boldsymbol{\ell }),V(\boldsymbol{\ell }),C^{I}(\boldsymbol{\ell }),C^{Y}(\boldsymbol{\ell }))= ( 600,1.31.2,10,0.4,3.5,0.5,0.1 ) $, where $\boldsymbol{\ell }\in {}[ -1,0]$.

**Figure 1:**
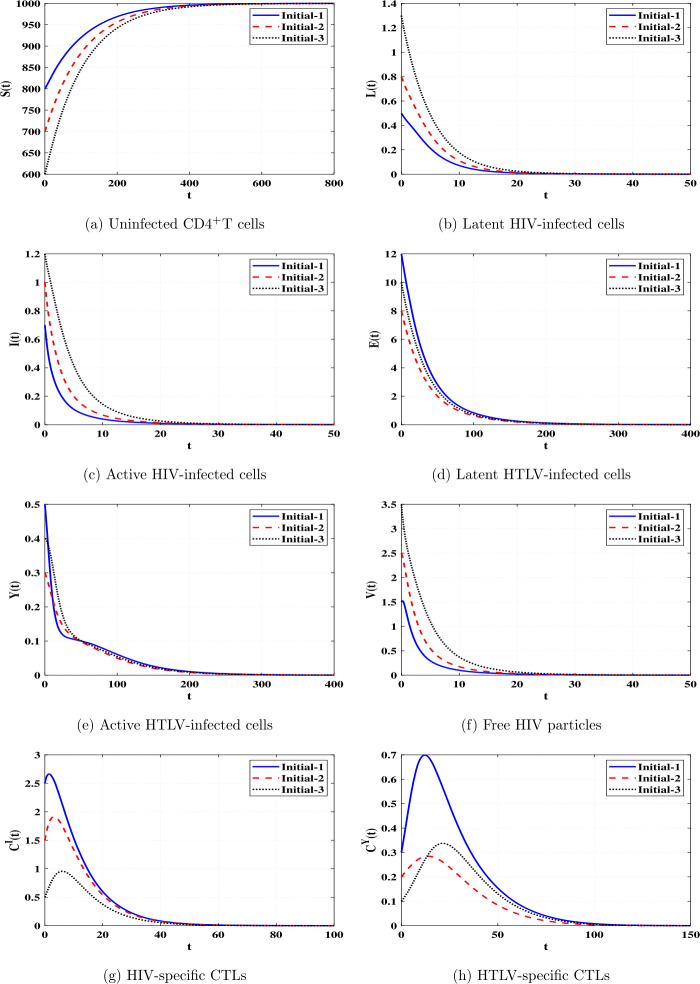
Solutions of system (29) when $\Re _{1}\leq 1$ and $\Re _{2}\leq 1$

Choosing different values of $\vartheta _{1}$, $\vartheta _{2}$, $\vartheta _{3}$, $\vartheta _{4}$, $\sigma _{1}$, and $\sigma _{2}$ under the above initial conditions leads to the following sets:

*Set 1 (Stability of* *)*: $\vartheta _{1}=0.0002$, $\vartheta _{2}=0.0001$, $\vartheta _{3}=0.001$, $\sigma _{1}=0.3$, and $\sigma _{2}=0.5$. For this set of parameters, we have $\Re _{1}=0.76<1$ and $\Re _{2}=0.27<1$. Figure 1 illustrates that the trajectories starting different initials converge to the steady state . This supports the global stability result of Theorem 1. Here, a healthy state will be reached where both viruses are absent.

**Figure 2:**
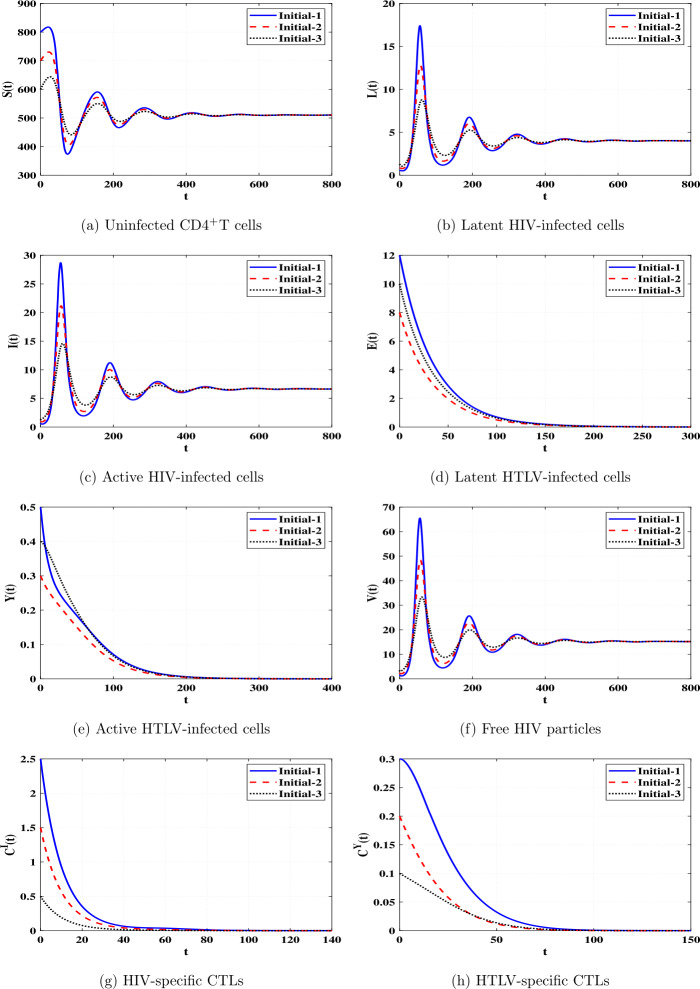
Solutions of system (29) when $\Re _{1}>1$, $\Re _{2}/\Re _{1}\leq 1$, and $\Re _{3}\leq 1$

*Set 2 (Stability of* *)*: $\vartheta _{1}=0.0005$, $\vartheta _{2}=0.0003$, $\vartheta _{3}=0.0007$, $\sigma _{1}=0.003$, and $\sigma _{2}=0.2$. With such a choice we get $\Re _{2}=0.19<1<1.96=\Re _{1}$, $\Re _{3}=0.34<1$, and hence $\Re _{2}/\Re _{1}=0.1<1$. The steady state  exists with . The stability of  given in Theorem 2 is shown in Fig. 2. This leads to the case where HIV monoinfection is chronic but with an ineffective CTL immunity.

**Figure 3:**
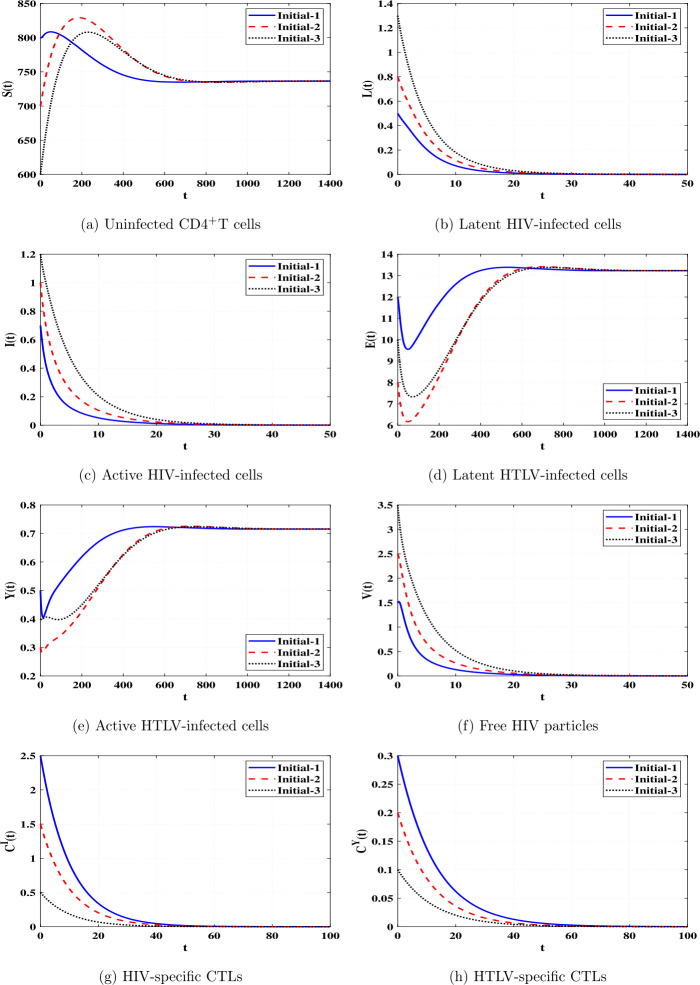
Solutions of system (29) when $\Re _{2}>1$, $\Re _{1}/\Re _{2}\leq 1$, and $\Re _{4}\leq 1$

*Set 3 (Stability of* *)*: $\vartheta _{1}=0.0001$, $\vartheta _{2}=0.0003$, $\vartheta _{3}=0.005$, $\sigma _{1}=0.001$, and $\sigma _{2}=0.05$. Then we calculate $\Re _{1}=0.72<1<1.34=\Re _{2}$, $\Re _{4}=0.67<1$, and then $\Re _{1}/\Re _{2}=0.54<1$ and . We can see from Fig. 3 that the system’s solutions tend to , which is compatible with Theorem 3. This case means that an HTLV monoinfection is chronic with an ineffective CTL immunity.

**Figure 4:**
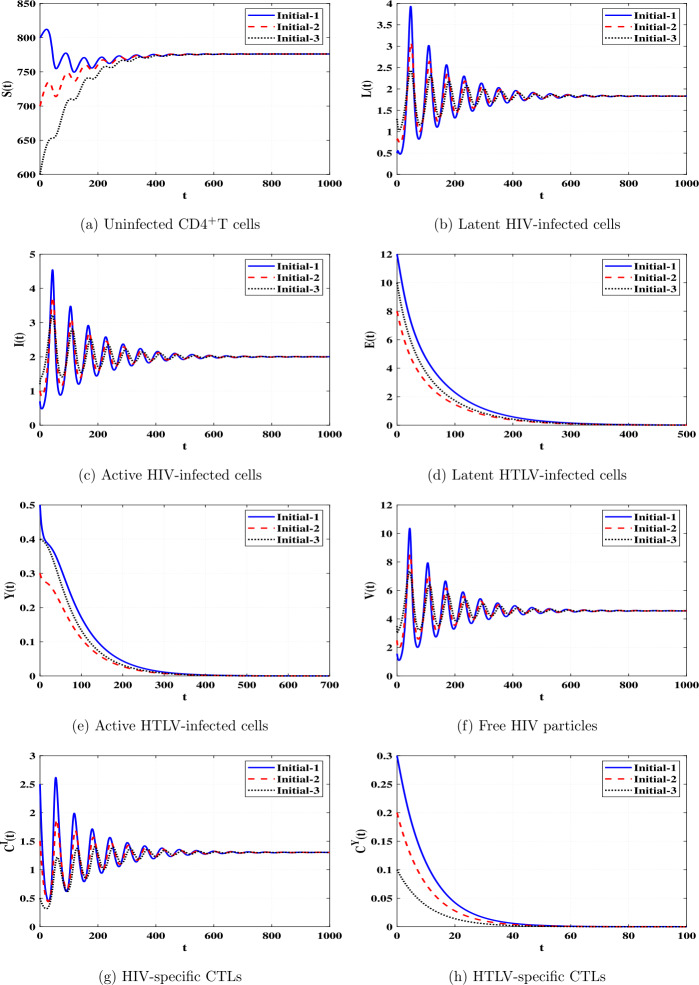
Solutions of system (29) when $\Re _{3}>1$ and $\Re _{5}\leq 1$

*Set 4 (Stability of* *)*: $\vartheta _{1}=0.0005$, $\vartheta _{2}=0.0003$, $\vartheta _{3}=0.002$, $\sigma _{1}=0.05$, and $\sigma _{2}=0.005$. Then we calculate $\Re _{3}=1.52>1$ and $\Re _{5}=0.42<1$. Figure 4 shows that the trajectories starting with different states tend to . Therefore,  is GAS, and this supports Theorem 4. Hence, an HIV monoinfection is chronic with effective HIV-specific CTL immunity.

**Figure 5:**
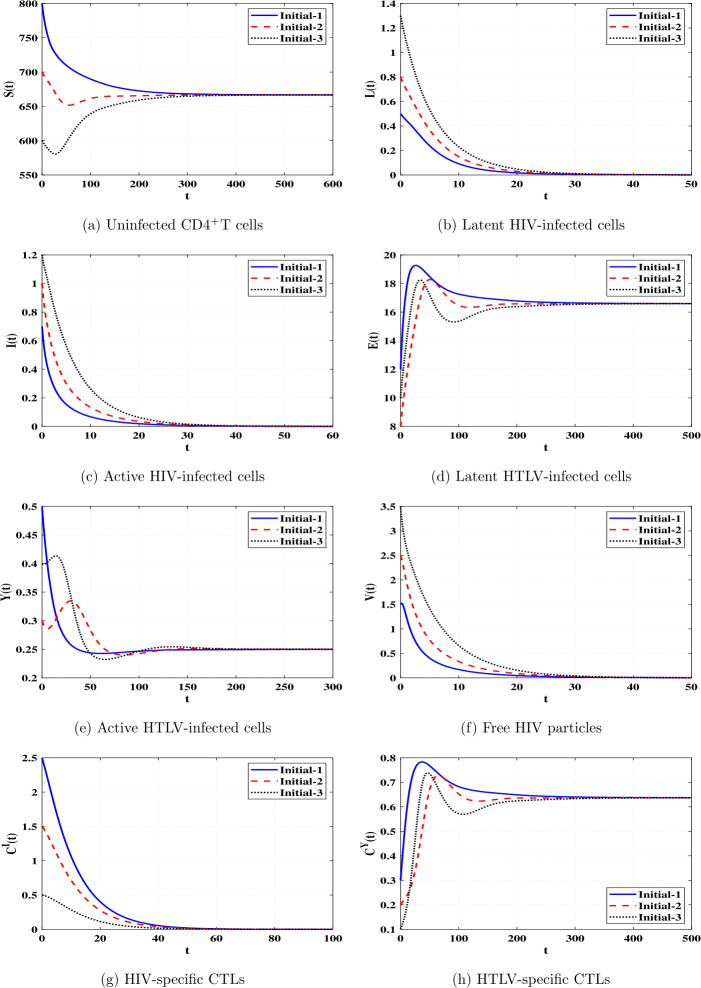
Solutions of system (29) when $\Re _{4}>1$ and $\Re _{6}\leq 1$

*Set 5 (Stability of* *)*: $\vartheta _{1}=0.0002$, $\vartheta _{2}=0.0002$, $\vartheta _{3}=0.02$, $\sigma _{1}=0.07$, and $\sigma _{2}=0.4$. Then we calculate $\Re _{4}=3.57>1$ and $\Re _{6}=0.60<1$, and  exists with . We observe from Fig. 5 that the system’s trajectories tend to  and it is GAS. Here, an HTLV monoinfection is chronic with effective HTLV-specific CTL immunity.

**Figure 6:**
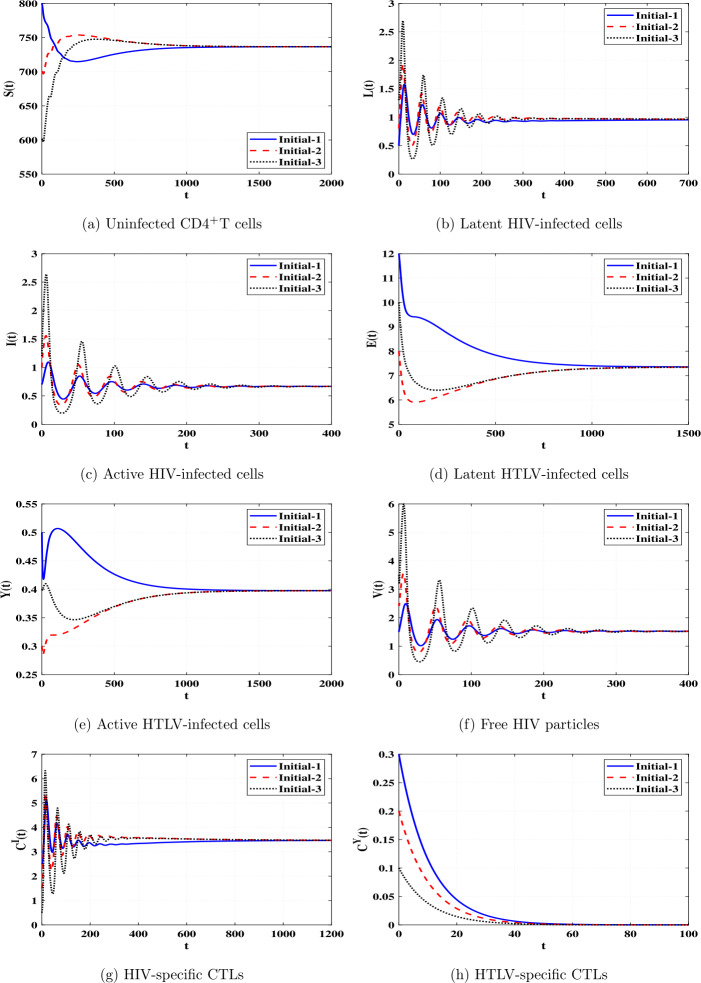
Solutions of system (29) when $\Re _{5}>1$, $\Re _{8}\leq 1$, and $\Re _{1}/\Re _{2}>1$

*Set 6 (Stability of* *)*: $\vartheta _{1}=0.001$, $\vartheta _{2}=0.0001$, $\vartheta _{3}=0.005$, $\sigma _{1}=0.15$, and $\sigma _{2}=0.01$. Then we calculate $\Re _{5}=1.15>1$, $\Re _{8}=0.22<1$, and $\Re _{1}/\Re _{2}=2.42>1$. The numerical solutions of the system drawn in Fig. 6 confirm that  exists and is GAS. This case leads to a chronic coinfection with HTLV and HIV where the HIV-specific CTL immunity is effective while the HTLV-specific CTL immunity is ineffective.

**Figure 7:**
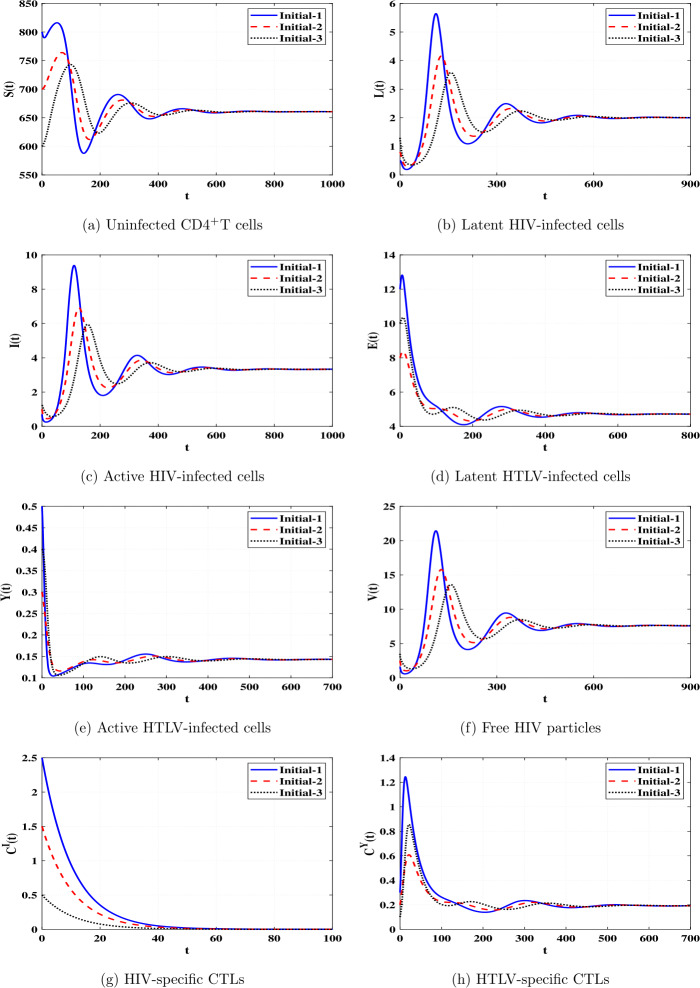
Solutions of system (29) when $\Re _{6}>1$, $\Re _{7}\leq 1$, and $\Re _{2}/\Re _{1}>1$

*Set 7 (Stability of* *)*: $\vartheta _{1}=0.0004$, $\vartheta _{2}=0.0002$, $\vartheta _{3}=0.01$, $\sigma _{1}=0.007$, and $\sigma _{2}=0.7$. We compute $\Re _{6}=1.32>1$, $\Re _{7}=0.55<1$, and $\Re _{2}/\Re _{1}=1.77>1$. According to these values, we obtain that  exists. The numerical solutions of our system plotted in Fig. 7 show that  is GAS (Theorem 7). This case leads to a chronic coinfection with HTLV and HIV where the HTLV-specific CTL immunity is effective and the HIV-specific CTL immunity is not working.

**Figure 8:**
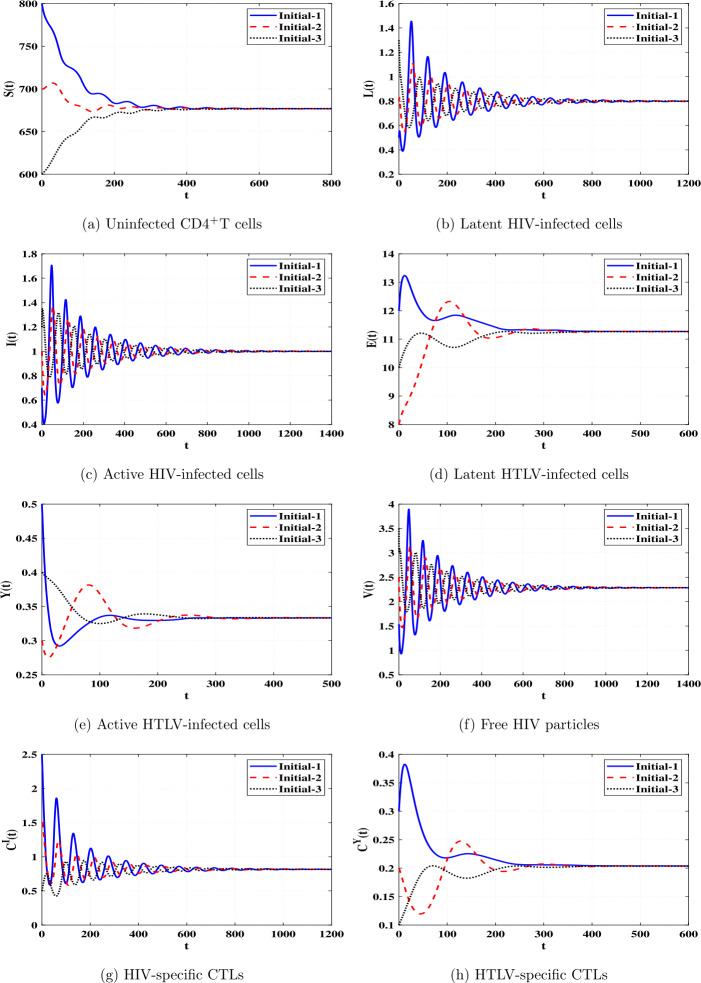
Solutions of system (29) when $\Re _{7}>1$ and $\Re _{8}>1$

*Set 8 (Stability of* *)*: $\vartheta _{1}=0.0005$, $\vartheta _{2}=0.0003$, $\vartheta _{3}=0.01$, $\sigma _{1}=0.1$, and $\sigma _{2}=0.3$. These data give $\Re _{7}=1.33>1$ and $\Re _{8}=1.81>1$. According to these values, the steady state  exists. Figure 8 illustrates that the solutions of the system initiating with three different states tend to . In this case, a chronic coinfection with HTLV and HIV is reached where both immune responses are well working.

### Effect of time delays on the HTLV-HIV dynamics

In this part we vary the delay parameters $\boldsymbol{\ell }_{i}$, $i=1,2,\ldots,6$, and fix the parameters $\vartheta _{1}=0.0005$, $\vartheta _{2}=0.0003$, $\vartheta _{3}=0.01$, $\sigma _{1}=0.04$, and $\sigma _{2}=0.7$. Since $\Re _{1}$ and $\Re _{2}$ given by Eqs. (30) and (31) depend on $\boldsymbol{\ell }_{i}$, $i=1,2,\ldots,6$, then changing the parameters $\boldsymbol{\ell }_{i}$ will change the stability of steady states. Let us consider the following situations:

(D.P.S1): $\boldsymbol{\ell }_{1}=\boldsymbol{\ell }_{2}=\boldsymbol{\ell }_{3}=\boldsymbol{\ell }_{4}=\boldsymbol{\ell }_{5}=\boldsymbol{\ell }_{6}=0$,
(D.P.S2): $\boldsymbol{\ell }_{1}=0.4$, $\boldsymbol{\ell }_{2}=0.5$, $\boldsymbol{\ell }_{3}=0.6$, $\boldsymbol{\ell }_{4}=0.7$, $\boldsymbol{\ell }_{5}=0.8$, and $\boldsymbol{\ell }_{6}=0.9$,
(D.P.S3): $\boldsymbol{\ell }_{1}=0.6$, $\boldsymbol{\ell }_{2}=0.7$, $\boldsymbol{\ell }_{3}=0.8$, $\boldsymbol{\ell }_{4}=0.9$, $\boldsymbol{\ell }_{5}=1$, and $\boldsymbol{\ell }_{6}=1.2$,
(D.P.S4): $\boldsymbol{\ell }_{1}=10$, $\boldsymbol{\ell }_{2}=11$, $\boldsymbol{\ell }_{3}=12$, $\boldsymbol{\ell }_{4}=13$, $\boldsymbol{\ell }_{5}=14$, and $\boldsymbol{\ell }_{6}=15$.

With these values we solve system (29) under the following initial condition:

*Initial-4*: $(S(\boldsymbol{\ell }),L(\boldsymbol{\ell }),I(\boldsymbol{\ell }),E(\boldsymbol{\ell }),Y(\boldsymbol{\ell }),V(\boldsymbol{\ell }),C^{I}(\boldsymbol{\ell }),C^{Y}(\boldsymbol{\ell }))= ( 800,1,2,4,0.14,3,1,0.1 ) $, where $\boldsymbol{\ell }\in {}[ -\max \boldsymbol{\ell }_{i},0]$, $i=1,2,\ldots,6$.

**Figure 9:**
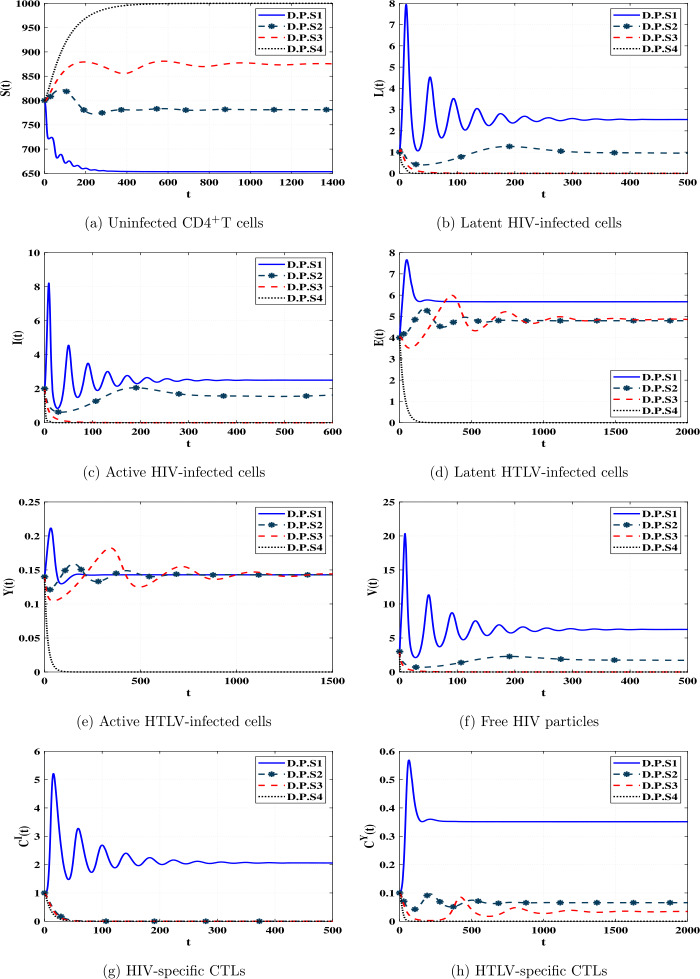
Impact of delay parameters $\ell _{i}$, $i=1,2,\ldots,6$, on the behavior of solution trajectories of system (29)

From Fig. 9 we observe that the presence of time delays can increase the number of uninfected CD4^+^ T cells and decrease the number of other compartments. Table 2 presents the values $\Re _{1}$ and $\Re _{2}$ for selected values of $\boldsymbol{\ell }_{i}$, $i=1,2,\ldots,6$. It is clear that $\Re _{1}$ and $\Re _{2}$ are decreased when $\boldsymbol{\ell }_{i}$ are increased, and thus the stability of  can be changed. Let us calculate the critical value of the time delay that changes the stability of . Without loss of generality, we let the parameters $\boldsymbol{\ell }=\boldsymbol{\ell }_{1}=\boldsymbol{\ell }_{2}=\boldsymbol{\ell }_{3}$ and fix $\boldsymbol{\ell }_{j}$, $j=5,6$, and write $\Re _{1}$ and $\Re _{2}$ as functions of ***ℓ*** and $\boldsymbol{\ell }_{4}$, respectively, as follows:

$$\begin{aligned} &\Re _{1}(\boldsymbol{\ell }) = \frac{ [ \lambda e^{-\boldsymbol{\ell } ( \hslash _{1}+\hslash _{3} ) } ( 1-\beta ) +\beta e^{-\hslash _{2}\boldsymbol{\ell }} ( \gamma +\lambda ) ] S_{0} ( b\vartheta _{1}e^{-\hslash _{6}\boldsymbol{\ell }_{6}}+\varepsilon \vartheta _{2} ) }{a\varepsilon ( \gamma +\lambda ) }, \\ &\Re _{2}(\boldsymbol{\ell }_{4}) = \frac{\varphi \vartheta _{3}\psi e^{- ( \hslash _{4}\boldsymbol{\ell }_{4}+\hslash _{5}\boldsymbol{\ell }_{5} ) }S_{0}}{ ( \delta -re^{-\hslash _{5}\boldsymbol{\ell }_{5}} ) \psi +\delta \omega }. \end{aligned}$$

To force the threshold parameters $\Re _{1}$ and $\Re _{2}$ to satisfy $\Re _{1}(\boldsymbol{\ell })\leq 1$ and $\Re _{2}(\boldsymbol{\ell }_{4}) \leq 1$, we choose $\boldsymbol{\ell }\geq \boldsymbol{\ell }^{\min }$, where $\boldsymbol{\ell }^{\min }$ is the solution of

$$ \frac{ [ \lambda e^{-\boldsymbol{\ell }^{\min } ( \hslash _{1}+\hslash _{3} ) } ( 1-\beta ) +\beta e^{-\hslash _{2}\boldsymbol{\ell }^{\min }} ( \gamma +\lambda ) ] S_{0} ( b\vartheta _{1}e^{-\hslash _{6}\boldsymbol{\ell }_{6}}+\varepsilon \vartheta _{2} ) }{a\varepsilon ( \gamma +\lambda ) }=1, $$

and

$$ \boldsymbol{\ell }_{4}\geq \boldsymbol{\ell }_{4}^{\min }, \quad \text{where } \boldsymbol{\ell }_{4}^{\min }= \max \biggl\{ 0,\frac{1}{\hslash _{4}}\ln \frac{\varphi \vartheta _{3}\psi e^{-\hslash _{5}\boldsymbol{\ell }_{5}}S_{0}}{ ( \delta -re^{-\hslash _{5}\boldsymbol{\ell }_{5}} ) \psi +\delta \omega } \biggr\} . $$

Therefore, if $\boldsymbol{\ell }\geq \boldsymbol{\ell }^{\min }$ and $\boldsymbol{\ell }_{4}\geq \boldsymbol{\ell }_{4}^{\min }$, then  is GAS. Let us choose the value $\boldsymbol{\ell }_{5}=0.2$ and $\boldsymbol{\ell }_{6}=0.1$ and compute $\boldsymbol{\ell }^{\min }$, $\boldsymbol{\ell }_{4}^{\min }$ as $\boldsymbol{\ell }^{\min }=2.73728$, $\boldsymbol{\ell }_{4}^{\min }=2.36794$. It follows that:

**Table 2:** The variation of $\Re _{1}$ and $\Re _{2}$ with respect to the delay parameters

Delay parameters	$\Re _{1}$	$\Re _{2}$
$\ell _{1}=\ell _{2}=\ell _{3}=\ell _{4}=\ell _{5}=\ell _{6}=0$	2.790	3.690
$\ell _{1}=0.3$, $\ell _{2}=0.4$, $\ell _{3}=0.5$, $\ell _{4}=0.6$, $\ell _{5}=0.7$, and $\ell _{6}=0.8$	1.408	1.787
$\ell _{1}=0.4$, $\ell _{2}=0.5$, $\ell _{3}=0.6$, $\ell _{4}=0.7$, $\ell _{5}=0.8$, and $\ell _{6}=0.9$	1.280	1.600
$\ell _{1}=0.6$, $\ell _{2}=0.7$, $\ell _{3}=0.8$, $\ell _{4}=0.9$, $\ell _{5}=1$, and $\ell _{6}=1.2$	1.009	1.283
$\ell _{1}=1$, $\ell _{2}=1.5$, $\ell _{3}=2$, $\ell _{4}=2.5$, $\ell _{5}=3$, and $\ell _{6}=3.5$	0.368	0.173
$\ell _{1}=2$, $\ell _{2}=3$, $\ell _{3}=4$, $\ell _{4}=5$, $\ell _{5}=6$, and $\ell _{6}=7$	0.188	0.008
$\ell _{1}=3$, $\ell _{2}=4$, $\ell _{3}=5$, $\ell _{4}=6$, $\ell _{5}=7$, and $\ell _{6}=8$	0.136	0.003
$\ell _{1}=4$, $\ell _{2}=6$, $\ell _{3}=8$, $\ell _{4}=9$, $\ell _{5}=10$, and $\ell _{6}=11$	0.072	0.1 × 10^−3^
$\ell _{1}=6$, $\ell _{2}=7$, $\ell _{3}=9$, $\ell _{4}=10$, $\ell _{5}=11$, and $\ell _{6}=12$	0.052	0.3 × 10^−4^
$\ell _{1}=10$, $\ell _{2}=11$, $\ell _{3}=12$, $\ell _{4}=13$, $\ell _{5}=14$, and $\ell _{6}=15$	0.016	1.2 × 10^−6^

(i) If $\boldsymbol{\ell }\geq 2.73728$ and $\boldsymbol{\ell }_{4}\geq 2.36794$, then $\Re _{1}(\boldsymbol{\ell })\leq 1$, $\Re _{2}(\boldsymbol{\ell }_{4}) \leq 1$ and  is GAS.

(ii) If $\boldsymbol{\ell }<2.73728$ or $\boldsymbol{\ell }_{4}<2.36794$, then $\Re _{1}(\boldsymbol{\ell })>1$ or $\Re _{2}(\boldsymbol{\ell }_{4})>1$ and  will lose its stability.

## Data Availability

Not applicable.
